# Effects of Graphene Quantum Dots on Renal Fibrosis Through Alleviating Oxidative Stress and Restoring Mitochondrial Membrane Potential

**DOI:** 10.1002/advs.202410747

**Published:** 2024-12-30

**Authors:** Kyu Hong Kim, Jong Bo Park, Jung Nam An, Gaeun Bae, Kyu Hyeon Kim, Seong Joon Park, Youngjin Jung, Yong Chul Kim, Jung Pyo Lee, Jae Wook Lee, Dong Ki Kim, Yon Su Kim, Byung Hee Hong, Seung Hee Yang

**Affiliations:** ^1^ Department of Biomedical Sciences Seoul National University Seoul South Korea; ^2^ Department of Chemistry, College of Natural Sciences Seoul National University Seoul South Korea; ^3^ R&D Center of Graphene Square Chemical Inc. Seoul South Korea; ^4^ Department of Internal Medicine Hallym University Sacred Heart Hospital Anyang South Korea; ^5^ Department of Internal Medicine Seoul National University Hospital Seoul South Korea; ^6^ Department of Internal Medicine Seoul National University Boramae Medical Center Seoul South Korea; ^7^ Nephrology Clinic National Cancer Center Goyang South Korea; ^8^ Department of Kidney Research Institute Seoul National University Medical Research Center Seoul South Korea; ^9^ Graduate School of Convergence Science and Technology Seoul National University Suwon South Korea; ^10^ Graphene Research Center Advanced Institute of Convergence Technology Seoul National University Seoul South Korea; ^11^ Biomedical Research Institute Seoul National University Hospital Seoul South Korea

**Keywords:** TRPC5, graphene quantum dots, mitochondrial membrane potential, oxidative stress, renal fibrosis

## Abstract

Podocyte injury and proteinuria in glomerular disease are critical indicators of acute kidney injury progression to chronic kidney disease. Renal mitochondrial dysfunction, mediated by intracellular calcium levels and oxidative stress, is a major contributor to podocyte complications. Despite various strategies targeting mitochondria to improve kidney function, effective treatments remain lacking. This study investigates the potential of graphene quantum dots (GQDs) in mitigating renal fibrosis and elucidates their underlying mechanisms. In animal models of Adriamycin‐induced nephropathy and 5/6 subtotal nephrectomy, GQDs treatment exhibits anti‐inflammatory, anti‐fibrotic, and anti‐apoptotic effects by restoring podocyte actin structure. These therapeutic benefits are associated with the downregulation of transient receptor potential channel 5 (TRPC5) activity, which is related to kidney fibrosis and mitochondrial dysfunction. In vitro, GQDs suppress TRPC5, enhancing anti‐fibrotic and anti‐apoptotic effects by lowering calcium levels under oxidative stress and mechanical pressure. Anti‐oxidative and anti‐senescent effects are also confirmed. Most significantly, transcriptomics and electron microscopy analyses reveal that GQD treatment enhances mitochondrial respiration‐related gene profiles and improves mitochondrial cristae morphology. These findings suggest that GQDs are a promising therapeutic nanomaterial for renal cell damage, capable of modulating calcium‐dependent apoptosis associated with mitochondrial injury, potentially slowing fibrosis progression.

## Graphical abstract

This study explores the potential of graphene quantum dots (GQDs) as therapeutic agents for kidney diseases. Using Adriamycin‐induced nephropathy and 5/6 nephrectomy models, GQDs significantly improved renal function, reduced inflammation and fibrosis, and protected against mitochondrial dysfunction. The study highlights the role of GQDs in regulating TRPC5 channels and mitigating oxidative stress, suggesting a novel approach to treating chronic kidney conditions.

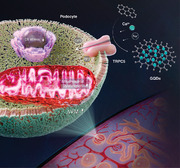

## 1 Introduction

Carbon‐based nanomaterials are widely used in biosensing, tissue engineering, imaging, diagnosis, drug delivery, and cancer therapy. Advancements in the chemistry and engineering of nanomaterials have enabled clinicians to effectively target the glomerulus and tubule due to the small size and electrochemical properties of these materials, which are relevant to kidney functions.^[^ ^1^ ^]^ Among these, graphene quantum dots (GQDs) have emerged as potential therapeutic agents for inflammatory and oxidative stress due to their biocompatibility, nanoscale size, stable fluorescence, water solubility, and low cytotoxicity.^[^ ^2^ ^]^ GQDs have shown efficacy in preventing α‐syn fibrillization, rescuing neuronal death, and mitigating synaptic loss in chronic neurodegenerative conditions.^[^ ^3^ ^]^ GQD‐based drug delivery systems have also been developed.^[^ ^4^ ^]^ Despite these advancements, the mechanisms underlying the role of GQDs as kidney‐specific nano‐pharmaceuticals have not yet been elucidated.

Mitochondria are crucial for energy production and cell survival. Oxidative stress caused by hypoxic damage induces mitochondrial DNA damage and mutations, leading to enzymatic abnormalities and further oxidative stress.^[^ ^5^ ^]^ The resulting mitochondrial dysfunction reduces adenosine triphosphate (ATP) synthesis, disrupts calcium (Ca^2+^) homeostasis, and affects central metabolic pathways. These issues contribute to the development of various kidney diseases, involving disruptions in mitochondrial homeostasis, aberrations, impaired biogenesis, cristae loss, reduced fusion and mitophagy, and subsequent mitochondrial fragmentation.^[^ ^6^ ^]^

Ca^2+^ is an essential secondary messenger that triggers numerous downstream cellular events in different kidney regions, such as vasopressin release in the terminal part of the inner medullary collecting duct and actin dynamics in podocytes and tubular cells. Mitochondria act as intracellular Ca^2+^ stores and Ca^2+^ signaling mediates crosstalk between the endoplasmic reticulum and mitochondria. Alterations in the endoplasmic reticulum Ca^2+^ channel rapidly increase mitochondrial and cytosolic Ca^2+^ levels, thereby contributing to apoptotic cell death.^[^ ^7^ ^]^ Disruption of intracellular Ca^2+^ homeostasis has been associated with the overexpression of transient receptor potential cation (TRPC) channels.^[^ ^8^ ^]^ Among these channels, TRPC5 is widely distributed in the plasma membrane of podocytes, serving as an important mediator of cytoskeletal changes. Increased Ca^2+^ influx through this channel plays a key role in the mitochondrial damage in podocytes and the progression of kidney injury, leading to glomerulosclerosis.^[^ ^9^ ^]^ TRPC5 inhibition has been shown to improve glomerular damage in proteinuric rodent models.^[^ ^10^ ^]^

Adriamycin‐induced nephropathy (ADN) is a representative model of chronic proteinuric renal disease, characterized by glomerulosclerosis, tubulointerstitial inflammation, and fibrosis after podocyte injury. The 5/6 subtotal nephrectomy (5/6Nx) model, on the other hand, is characterized by persistent decreases in the glomerular filtration rate, proteinuria, glomerular sclerosis, and hypertension. GQDs with oxygenated functional groups exhibit high aqueous solubility and can effectively incorporate various cations in the human body.^[^ ^11^ ^]^ The photoluminescence quenching in GQDs is a well‐documented physicochemical phenomenon for metals or lanthanides such as Cu^2+^, Cr^6+^, Ni^2+^, Co^2+^, Mn^2+^, Al^3+^, and Ce^3+^.^[^ ^12^ ^]^ Recent evidence indicates that graphene oxides or GQDs can bind to Ca^2+^ in aqueous solution, with ‐COOH groups playing an important role in this binding.^[^ ^13^ ^]^ Utilizing GQDs in the renal system can help balance intracellular Ca^2+^ levels and prevent kidney disease development by enhancing mitochondrial membrane potential.

In this study, we aimed to explore the potential of GQDs in mitigating inflammation, fibrosis, aging, and apoptosis by addressing mitochondrial dysfunction and aberrations through the regulation of Ca^2^⁺ channel activity in ADN and 5/6Nx models.

## 2 Results

### 2.1 GQDs Properties

The average diameter of the GQDs was 2.5 ± 0.5 nm, as determined from HR‐TEM images. The d‐spacing of the lattice in the GQDs, measured using Cs‐TEM images, was 0.33 nm (Figure S1A,B, Supporting Information). Additionally, the chemical components and molecular interactions of the GQDs were evaluated using FT‐IR, Raman, and XPS C1 spectra, with results shown in Figure S1C–E (Supporting Information).

### 2.2 GQDs Improve Renal Function and Ameliorate Renal Inflammation and Fibrosis

**Figure 1:**
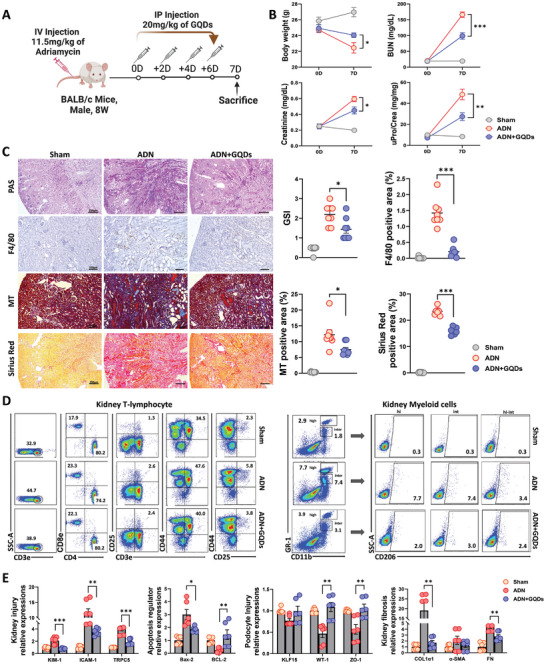
Ameliorating effect of GQDs on renal injury in an ADN mouse model. A) A schematic of the ADN+GQDs mouse model, AD (11.5 mg kg^−1^, I.V) and GQDs (10 or 20 mg kg^−1^, I.P.) were injected into BALB/c (Male, 8 W) mice (*n* = 8). B) The body weights, BUN, creatinine level, and urine protein/Cr ratio of mice after AD administration (*n* = 8 per group) are shown. C) Immunohistochemistry images and the quantification results of kidney sections from AD+GQDs treated mice at 7 days. GSI, glomerulosclerosis index. Sham (*n* = 6), ADN (*n* = 8), and ADN+GQDs (*n* = 8). Scale bars, 250 µm (40×) and 100 µm (100×). D) Kidney‐resident immune cell types in the ADN mouse model, representing 2 populations [kidney T‐cells, CD3e^+^CD8e^+^CD4^+^CD25^+^CD44^+^; kidney myeloid cells, CD11b^+^GR‐1^+^CD206^+^], were determined by flow cytometry. E) Band intensity of western blot results was determined by densitometric quantification and normalized to GAPDH (*n* = 6 per group). Experiments were repeated at least 3 times, and the data are shown as the mean ± standard error of the mean. *p < 0.05, **p < 0.01, ***p < 0.001.

To explore the roles of GQDs in the ADN mice, we first evaluated kidney function and the pathological characteristics. The schematic diagram of the ADN mouse model is illustrated in **Figure** **1**A. The treatment with GQDs led to an increase in body weight, a decrease in BUN and serum Cr levels, as well as a reduction in the urinary protein‐to‐Cr ratio (Figure 1B), suggesting that GQDs have a reno‐protective effect. The immunohistochemistry (IHC) results revealed that glomerular sclerosis/tubular injury (GSI), F4/80, MT, and Sirius Red positive stained areas significantly decreased in GQDs‐treated mice (Figure 1C). We determined the frequency of T‐cell recruitment and myeloid cells using FACS analysis. The FACS results showed that the CD3^+^CD25^+^CD44^+^ T lymphocyte content in the GQDs‐treated group decreased from 5.8% to 3.8%, and the distribution pattern of 3 myeloid subpopulations of CD11b^+^Gr1^+^CD206^+^ (HI, INT, HI‐INT) returned to normal: 7.7% to 2.0% for CD11b^+^Gr1^+^CD206^+^ (HI), 7.4% to 3.0% for CD11b^+^Gr1^+^CD206^+^ (INT), and 3.4% to 2.4% for CD11b^+^Gr1^+^CD206^+^ (HI‐INT) (Figure 1D; Figure S2, Supporting Information). These results indicate that GQDs prevent the infiltration of myeloid cells with a phenotype like myeloid‐derived suppressor cells (CD11b^+^GR1^+^ cells) and reduce their crosstalk with activated T‐cells, thereby inhibiting further kidney injury. The protein expression of ICAM‐1 (intercellular adhesion molecule 1), KIM‐1 (kidney injury molecule‐1), Bax‐2 (BCL‐2 associated X), Col1α1 (collagen type 1 alpha 1), FN (Fibronectin), and α‐SMA (alpha‐smooth muscle actin) were significantly downregulated in the GQDs‐treated group (Figure 1E; Figure S3, Supporting Information).

Next, to determine whether GQDs have anti‐apoptotic role and rescue podocytes, the protein levels of BCL‐2 (B‐cell lymphoma 2), WT‐1 (Wilm's tumor gene 1), ZO‐1 (Zonula occludens‐1), and KLF‐15 (Kruppel‐like transcription factor 15) were assessed through western blotting. The BCL‐2, WT‐1, ZO‐1, and KLF‐15 protein expression levels were restored in the GQDs‐treated group compared with that in the ADN group. To determine which TRPC channel is a therapeutic target in the ADN mouse model, we measured the protein expression of both channels. Interestingly, TRPC5 expression was significantly decreased compared with TRPC6 expression following treatment with GQDs (Figure 1E; Figures S3 and S10A, Supporting Information). We also observed the specific interaction between GQDs and Ca^2+^ ions. The negatively charged surface of GQDs facilitates their binding with Ca^2+^ ions (Figure S1F,G, Supporting Information), leading to a significant decrease in PL intensity (Figure S1H–L, Supporting Information), and inducing chemical crosslinking of GQDs (Figure S1M, Supporting Information).

To further explore specific regulatory genes, mRNA‐seq was performed using these samples. 3D principal component analysis and scatter plots demonstrated clear separation between the groups and distribution of DEGs based on expression intensity (Figure S4A,B, Supporting Information). Our analysis identified 1841 genes (*p < 0.05*) altered by GQDs treatment in AD‐administered mice: 677 upregulated and 1164 downregulated genes. GO enrichment analysis revealed that the top 10 upregulated gene clusters were associated with cellular metabolism and ion transport, while downregulated gene clusters were associated with inflammation‐related pathways (Figure S5A–D, Supporting Information).

**Figure 2:**
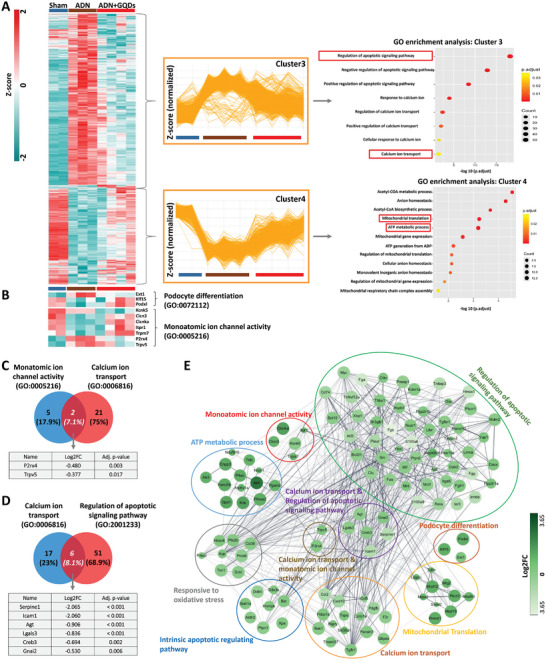
A) A global heatmap of DEGs shows the number of genes, orientated by their Z‐scores (*p < 0.05*). Two clusters based on DEG patterning are identified, and dot plots constructed from GO enrichment analysis are presented. Dot plots are displayed with ‐log 10 (p.adjust) values and the number of genes involved. Sham (*n* = 2), ADN (*n* = 3), and ADN+GQDs (*n* = 4). B) A heatmap represents podocyte differentiation and Monoatomic ion channel. C, D) Common genes between monoatomic ion channel activity and Ca^2+^ ion transport (C) and between Ca^2+^ ion transport and regulation of apoptotic signaling pathway (D), respectively. E) A protein interaction network analysis highlighting strong interactions and intricate connections among genes, clustered by biological process description from GO analysis. Experiments were repeated at least 3 times, and the data are shown as the mean ± standard error of the mean. *p < 0.05, **p < 0.01, ***p < 0.001.

**Figure 3:**
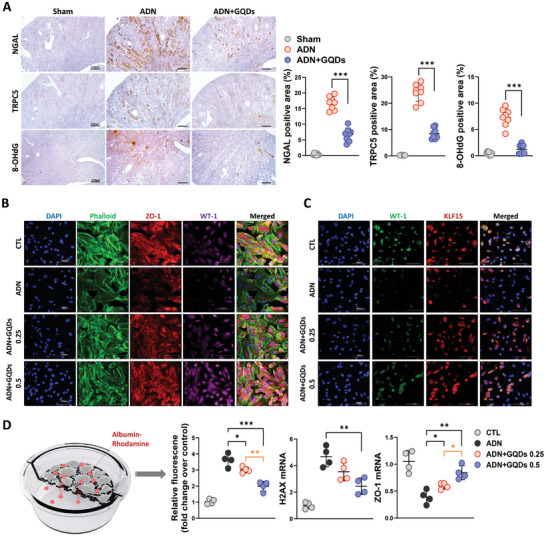
A) Representative images and quantification of NGAL, TRPC5, and 8‐OHdG. Sham (*n* = 8), ADN (*n* = 8), and ADN+GQDs (*n* = 8). B, C) Immunofluorescence imaging for human podocytes after exposure to AD (10 ng mL^−1^) and GQDs (0.25 or 0.5 µg mL^−1^). Scale bar, 100 µm. (CTL = Control) D) Relative fluorescence of albumin‐rhodamine diffusion (left) and mRNA profiles (right) of human podocytes are shown. Scale bar, 100 µm. AD (10 ng mL^−1^) and GQDs (0.25 or 0.5 µg mL^−1^). Control (CTL) (*n* = 4), ADN (*n* = 4), ADN+GQDs 0.25 (*n* = 4), ADN+GQDs 0.5 (*n* = 4). Experiments were repeated at least 3 times, and the data are shown as the mean ± standard error of the mean. *p < 0.05, **p < 0.01, ***p < 0.001.

Further analysis of global gene expression patterns across the 3 groups: sham, ADN, and ADN+GQDs, revealed 4 clusters of gene sets (Figure S6, Supporting Information). GO enrichment analysis indicated significant inhibition of genes related to Ca^2+^ ion transport (GO:0 006816) and regulation of the apoptotic signaling pathway (GO:2 001 233) in the GQDs‐treated mice compared with those treated with AD alone. Conversely, genes involved in mitochondrial translation (GO:00 32543) and the ATP metabolic process (GO:00 46034) were significantly upregulated (**Figure** **2**A; Figures S7 and S8, Supporting Information). Both the intrinsic apoptotic signaling pathway (GO:00 97193) and response to oxidative stress (GO:0 006979) were downregulated in the GQDs‐treated group compared with those in the other groups (Figure S7, Supporting Information). Our mRNA‐seq and western blot data show that GQDs suppress the TRPC5, thereby promoting podocyte differentiation (Figure 2B) and decreasing apoptosis through enhanced mitochondrial biogenesis and membrane potential. Specific genes regulating Ca^2+^ ion transport and apoptosis were identified, including *P2rx4* and *Trpv5*, which encode TRPV5 (transient receptor potential cation channel subfamily V member 5) and P2X4R (P2X purinoceptor 4) (Figure 2C). These proteins are involved in T‐cell proliferation and immune cell activation,^[^ ^14^ ^]^ as supported by our FACS data, which indicated a reduction in CD3^+^CD25^+^CD44^+^ T‐cells and CD11b^+^GR‐1^+^ CD206^+^ myeloid cells. Additionally, genes implicated in Ca^2+^ ion transport‐induced apoptotic pathways were identified, including *Serpine 1*, *Icam‐1*, *Agt*, *Lgals3*, *Creb3*, and *Gnai2* (Figure 2D). In the kidney, TRPC5 predominantly modulates the TRPV5 transporter, along with other G‐protein‐coupled receptors, leading to increased intracellular Ca^2+^ influx^[^ ^15^ ^]^ and impaired mitochondrial function. Our findings suggest that reduced expression of the *Trpv5* gene by GQDs can disrupt the TRPC5 signaling pathway. To elucidate inter‐ and intra‐class gene correlations, we constructed a molecular network based on the GO enrichment analysis in the ADN model (Figure 2E). Collectively, our results indicate that GQDs mitigate renal apoptosis and prevent podocytopathies by suppressing Ca^2+^ ion transport through TRPC5 and enhancing mitochondrial membrane potential in the ADN mouse model. According to the IHC results, the areas positively stained for Neutrophil gelatinase‐associated lipocalin (NGAL), TRPC5, and 8‐OHdG (an oxidative DNA damage marker) in regions with glomerular sclerosis were decreased after GQDs administration compared with that observed in the AD‐injected group (**Figure** **3**A). Immunofluorescence co‐staining of phalloidin (a marker of the cytoskeleton), ZO‐1, WT‐1, and KLF15 showed enhanced expression of podocyte differentiation activity in response to GQDs treatment. This was accompanied by restoring podocyte morphology, number, and cytoskeleton integrity (Figure 3B,C). The relative fluorescence of albumin‐rhodamine was reduced by 2‐fold in the group treated with 0.5 µg mL^−1^ GQDs compared with the AD‐treated group. Additionally, the mRNA expression levels of H2AX (a DNA damage marker) decreased, while the podocyte junction marker ZO‐1 increased in the GQDs‐treated group (Figure 3D). In agreement with these observations, GQDs restore podocyte foot processes by activating podocyte cytoskeleton differential genes and mediating the expression of TRPC5 under ADN model.

### 2.3 GQDs Attenuate Kidney Fibrosis Associated with TRPC5 Activation

**Figure 4:**
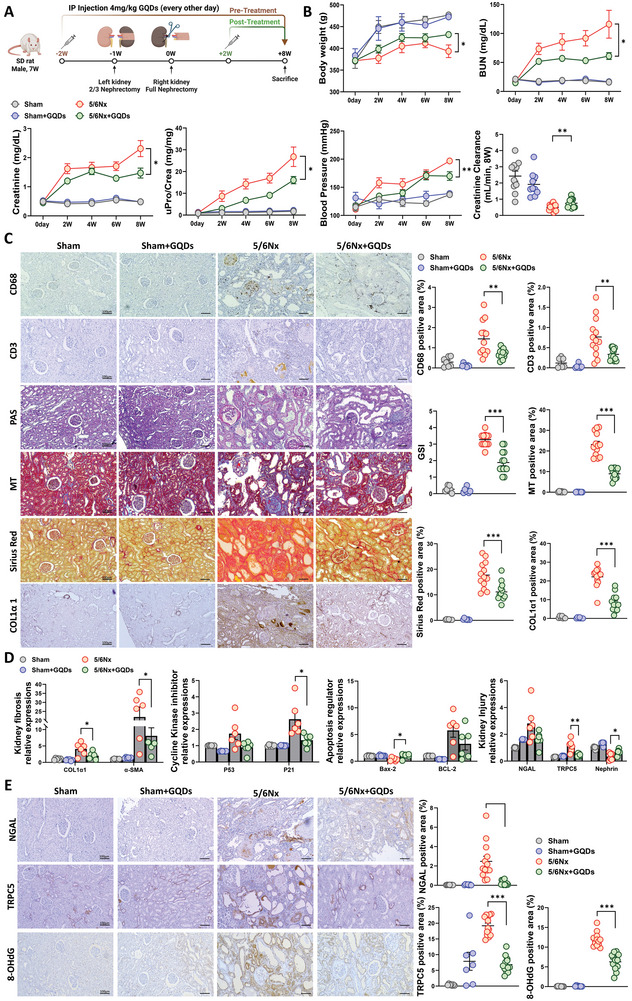
GQDs attenuated kidney fibrosis and contributed to kidney function recovery in the 5/6Nx model (pre‐treatment group). A) A schematic of the 5/6Nx+GQDs rat model B) Body weights, kidney function indicators, blood pressure, and Cr clearance in the GQDs pre‐treated group after 5/6Nx. Sham (*n* = 6), Sham+GQDs (*n* = 3), 5/6Nx (*n* = 9), and 5/6Nx+GQDs (*n* = 9). Cr clearance; Sham (*n* = 10), Sham+GQDs (*n* = 10), 5/6Nx (*n* = 13), and 5/6Nx+GQDs (*n* = 13). C) Representative images from immunohistochemical staining (right) and corresponding measurements (left) in the GQDs pre‐treated group. Sham (*n* = 7), Sham+GQDs (*n* = 7), 5/6Nx (*n* = 13), and 5/6Nx+GQDs (*n* = 13). Scale bar, 100 µm. D) Immunoblot analysis of samples from 5/6Nx rats that received GQDs. Band intensities are determined by densitometric quantification and normalized to β‐actin (*n* = 6 per group). E) Scanned images of kidney sections show NGAL, TRPC5, and 8‐OHdG, along with their corresponding measurements. Sham (*n* = 7), Sham+GQDs (*n* = 7), 5/6Nx (*n* = 13), and 5/6Nx+GQDs (*n* = 13). Scale bar, 100 µm. Experiments are repeated at least 3 times, and the data are shown as the mean ± standard error of the mean. *p < 0.05, **p < 0.01, ***p < 0.001.

Based on evidence of GQDs’ protective effects in the ADN mouse model, we expanded our study to explore the potential therapeutic role of GQDs in the 5/6Nx rat model (**Figure** **4**A). In the model, pre‐treatment with GQDs resulted in improvements, including increased body weight, reduced blood pressure, and decreased levels of BUN, serum Cr, and proteinuria (Figure 4B). Cr clearance was significantly lower in the 5/6Nx group but improved following GQDs pre‐treatment. The areas stained for glomerulus and tubular damage (tubular atrophy, interstitial inflammation, glomerular sclerosis, interstitial fibrosis), CD3e, CD68, and COL1α1 were significantly reduced after GQDs pre‐treatment (Figure 4C). Western blotting data indicated decreased COL1α1, α‐SMA, P53, P21, Bax‐2, NGAL, and TRPC5 expression in the GQDs pre‐treated group. Conversely, levels of Nephrin and BCL‐2 were increased (Figure 4D; Figures S9 and S10B, Supporting Information). Further, IHC results revealed increased levels of NGAL, TRPC5, and 8‐OHdG in the kidney tissue of the 5/6Nx group, with decreased levels following GQDs pre‐treatment (Figure 4E). Collectively, GQDs prolong the therapeutic effect by suppressing TRPC5 and ameliorating kidney injury during the development of kidney fibrosis.

### 2.4 GQDs Rescue Cell Viability in an Oxidative Stress Model

**Figure 5:**
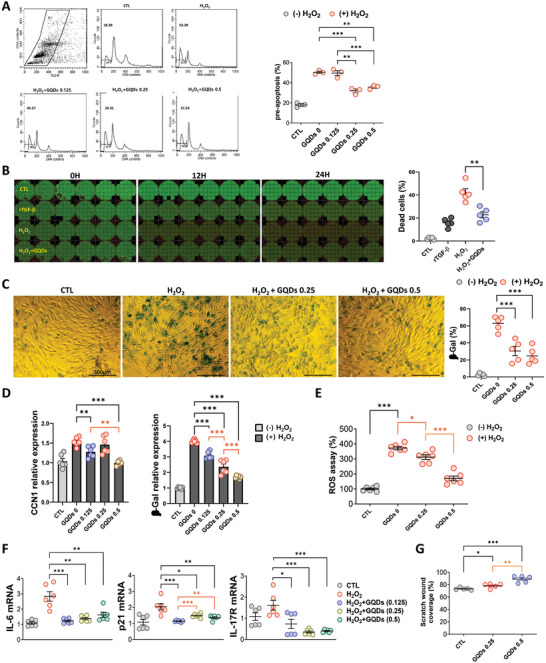
GQDs inhibited podocyte apoptosis in vitro. A) Cell cycle analysis in podocytes after incubation with H_2_O_2_ (0.5 mM) and GQDs (0.125, 0.25, or 0.5 µg mL^−1^). Representative flow cytometry plots and quantitative data are shown (*n* = 3 per group). B) Representative live and dead cell images (left) and quantitative data (right) show podocyte death under recombinant transforming growth factor (rTGF‐β) (2 ng mL^−1^) and H_2_O_2_ (1 mM)‐induced kidney injury with GQDs (0.5 µg mL^−1^) treatment (*n* = 5 per group). C) The β‐galactosidase assay shows the anti‐senescence effect on human podocytes after incubation with H_2_O_2_ (1 mM) and GQDs (0.25 or 0.5 µg mL^−1^) (*n* = 5 per group). Scale bar, 250 µm. D) Western blot analysis shows the level of β‐Gal and CCN1 in different groups (*n* = 6 per group). H_2_O_2_ (1 mM) and GQDs (0.125, 0.25, or 0.5 µg mL^−1^). E) Quantitative colorimetric ROS assay shows decreased ROS production of podocytes after incubation with H_2_O_2_ (1 mM) and GQDs (0.25 or 0.5 µg mL^−1^) (*n* = 4 per group). F) The mRNA expression of proinflammatory cytokine and kinase activity (*n* = 6 per group). H_2_O_2_ (1 mM) and GQDs (0.125, 0.25, or 0.5 µg mL^−1^) (G) Wound healing assay shows maximum improvement of injury in H_2_O_2_‐induced oxidative stress condition. Cells are pre‐treated with GQDs (0.25 or 0.5 µg mL^−1^) for 2 h before removing cells (*n* = 5 per group). Figures show the individual data from the different groups, with each experiment repeated at least 3 times. Data are shown as the mean ± standard error of the mean. *p < 0.05, **p < 0.01, ***p < 0.001.

Oxidative stress, characterized by excess production of ROS (Reactive oxygen species), is a significant factor contributing to chronic inflammation in kidney fibrosis. We evaluated the antioxidant and anti‐senescence roles of GQDs in podocytes. The proportion of pre‐apoptotic cells induced by H_2_O_2_ was significantly reduced following GQDs treatment. The effect appeared at 0.25 µg mL^−1^ concentration, indicating a ≈50% decrease (**Figure** **5**A). In the H_2_O_2_ treatment group, the number of dead cells increased after 12 h, indicating approximately twice that of the rhTGF‐β‐treated group. However, the co‐administration of GQDs reduced cell death by 50% (Figure 5B). Senescence shown through β‐galactosidase (β‐Gal) protein expression, also significantly decreased following co‐treatment with H_2_O_2_ and GQDs compared with those in the H_2_O_2_ treatment group (Figure 5C). The antioxidant effect of GQDs was also demonstrated through the CCN1 (Cellular communication network factor 1), β‐Gal, and ROS assays (Figure 5D,E; Figure S11, Supporting Information). Additionally, mRNA expression levels of inflammatory cytokines, cytokine receptors, and apoptosis markers increased in the H_2_O_2_ group but were significantly mitigated in the H_2_O_2_ and GQDs co‐treated group. This effect was observed even at low doses of GQDs (Figure 5F). Finally, the wound scratch assay revealed that cell migration and proliferation were significantly increased in a dose‐dependent manner in the GQDs‐treated group (Figure 5G; Figure S12, Supporting Information).

### 2.5 GQDs Restore Mitochondrial Function in ADN and 5/6Nx Models

**Figure 6:**
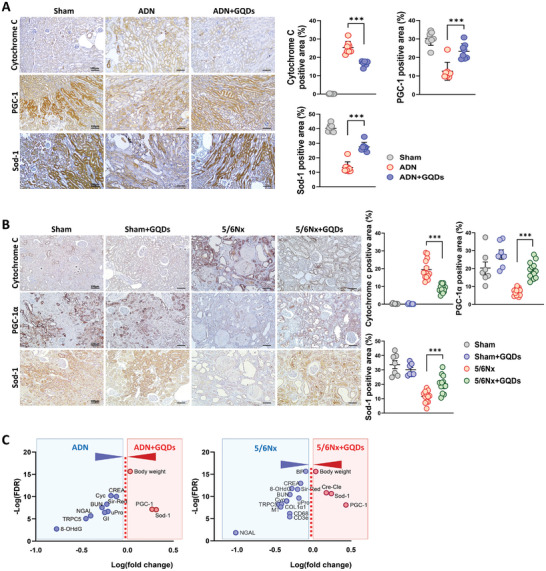
GQDs targeted mitochondria in renal injuries. A and B) Representative immunohistochemistry images and quantitative data of cytochrome C, PGC‐1α, and Sod‐1. (A), ADN model (*n* = 8 per group); (B), 5/6Nx model, Sham (*n* = 7), Sham+GQDs (*n* = 7), 5/6Nx (*n* = 13), and 5/6Nx+GQDs (*n* = 13). Scale bar, 100 µm. C) The plots illustrate IHC markers with kidney function measurements from the indicated study showing different hierarchical ranks based on –log(FDR) and log(fold‐change) values. FDR is calculated using the formula: FDR = *p*‐value (one‐sample t‐test) × (number of categories/rank). Three independent experiments with similar results were performed. Data are shown as the mean ± standard error of the mean. *p < 0.05, **p < 0.01, ***p < 0.001.

Chronic exposure to ROS can lead to oxidative damage in mitochondria, a common response to kidney injury and a key factor in the development of fibrosis. In the 5/6Nx and ADN groups, the expression of cytochrome C was increased, while the expressions of peroxisome proliferator‐activated receptor‐gamma coactivator 1‐alpha (PGC‐1α; an important regulator of mitochondrial biogenesis and energy metabolism) and superoxide dismutase (Sod‐1; an antioxidant enzyme) were decreased. In contrast, in the GQDs‐treated groups, the expression of cytochrome C was significantly decreased, and the expressions of PGC‐1α and Sod‐1 were increased (**Figure** **6**A,B). Figure 6C showed that in the 5/6Nx group, TRPC5 protein levels exhibited substantial changes and were significantly decreased with GQDs treatment. Moreover, treatment with GQDs decreased 8‐OHdG and cytochrome C expressions, increased Sod‐1 expression, and improved renal function, blood pressure, fibrosis, and inflammation. In the ADN model, the increase in PGC‐1α and Sod‐1 levels and the decrease in TRPC5 and 8‐OHdG levels after GQDs treatment were significant.

**Figure 7:**
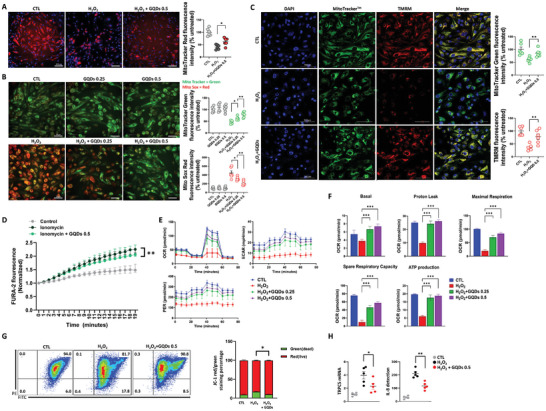
A) Representative images of podocytes labeled with MitoTracker Deep Red (mito T) and DAPI after 24 h of incubation with H_2_O_2_ (1 mM) and GQDs. Scale bar, 100 µm (*n* = 6 per group). B and C) Representative images of human podocytes loaded with MitoTracker (green) and MitoSOX (red) (B) or TMRM (red) C). (*n* = 6 per group). Scale bars, 50 µm and 500 µm. D) Intracellular Ca^2+^ responses measured by Fura‐2 AM radiometric fluorescence to ionomycin (5 mM) and GQDs (0.5 µg mL^−1^) (*n* = 3 per group). E) Cellular respiration and glycolysis in podocytes after exposure to H_2_O_2_ (1 mM) and GQDs (0.25 or 0.5 µg mL^−1^). OCR, oxygen consumption rate; ECAR, extracellular acidification rate; PER, proton efflux rate (*n* = 8 per group). F) Measurements of basal, proton leak, maximal respiration, spare respiratory capacity, and ATP production in each group (*n* = 8 per group). G) Mitochondrial membrane potential by JC‐1 dye staining and density plot. Dead (*n* = 9), Live (*n* = 9). H) The mRNA expression of TRPC5 and IL‐8 cytokine concentration (pg/mL) measured in response to H_2_O_2_‐induced mitochondrial dysfunction (*n* = 5 per group**)**. Three independent experiments with similar results were performed. Data are shown as the mean ± standard error of the mean. *p < 0.05, **p < 0.01, ***p < 0.001.

Our mRNA‐seq data indicated that AD‐associated podocyte injury is associated with decreased mitochondrial translation‐related gene profiles (Figure S7, Supporting Information). Podocytes treated with H_2_O_2_ (1 mM) and GQDs (0.25 or 0.5 µg mL^−1^) exhibited a significant increase in MitoTracker fluorescence intensity, indicating enhanced mitochondrial activity. Additionally, mitochondrial morphology underwent a transformation from short rods to a more filamentous shape (**Figure** **7**A; Figure S13, Supporting Information). Furthermore, co‐staining with MitoSOX (red) and MitoTracker (green) indicated that mitochondrial superoxide production in podocytes decreased after GQDs treatment under H_2_O_2_ exposure (Figure 7B). TMRM staining highlighted that GQDs enhance bioelectric activity in mitochondria (Figure 7C). Furthermore, GQDs significantly reduced the intracellular Ca^2+^ influx by restoring the increased cytosolic Ca^2+^ permeability after ionomycin treatment in podocytes (Figure 7D). The GQDs‐treated group had a higher extracellular acidification rate (ECAR) than the H_2_O_2_‐damaged group, implying that the glycolysis and glycolytic capacity of podocytes were reversed by the administration of GQDs (Figure 7E). OCR measurements displayed distinct bioenergetic profiles as the OCR was recovered in the GQDs‐treated group. Under H_2_O_2_‐induced oxidative stress in cultured podocytes, GQDs treatment led to a significant increase in basal OCR, proton leak, maximal respiration, spare respiratory capacity, and ATP production in a dose‐dependent manner (Figure 7F, Table S23, Supporting Information). Even at low doses, GQDs exhibited significant effects, with recovery comparable to that of the control. Proton leaks associated with uncoupled respiration also increased promptly in the GQDs‐treated group. The JC‐1 assay also demonstrated the recovery and improvement of the mitochondrial membrane potential in the GQDs‐treated group compared with that in the H_2_O_2_‐injured group (Figure 7G). Lastly, the decrease in mRNA expression of TRPC5 and IL‐8 cytokine concentration demonstrated that the restoration of renal cell mitochondria abated inflammation in response to oxidative stress after enhancing oxidative phosphorylation and balancing intracellular Ca^2+^ levels (Figure 7H). Based on the results for the ADN (Figure S14A, Supporting Information) and 5/6Nx (Figure S14B, Supporting Information) models, a significant correlation was observed between the expression of TRPC5 and fibrosis, tubular injury, inflammation, oxidative DNA damage, and mitochondrial damage.

**Figure 8:**
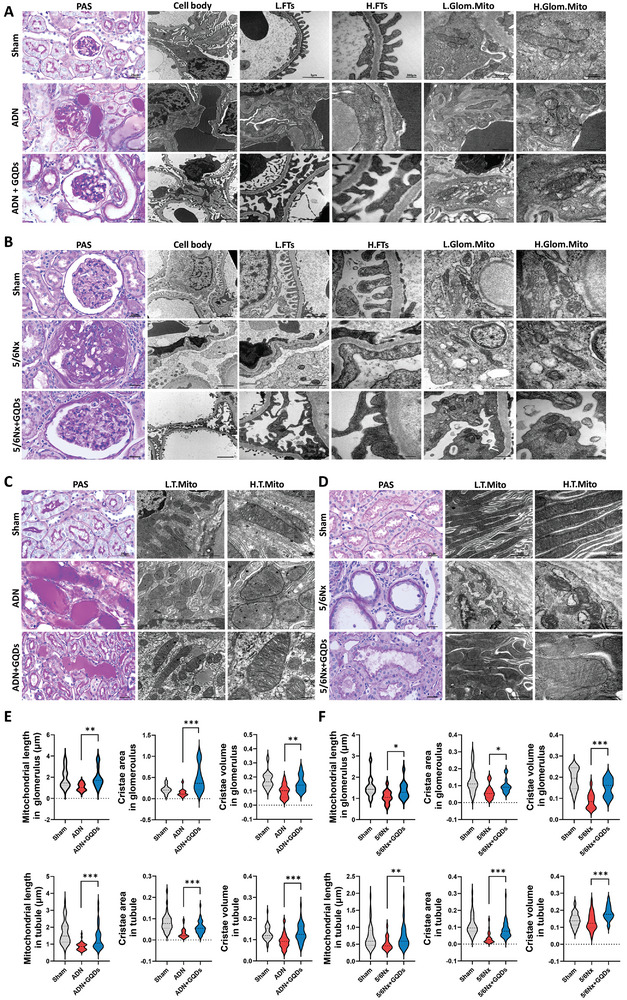
A and B) Representative PAS and TEM images of kidney sections, showing podocyte foot process (FT), mitochondria in the glomerulus (Glom. Mito), and C and D) mitochondria in tubule (T. Mito). E and F) Representative violin plots illustrate the changes in mitochondrial lengths, cristae area, and volume density following GQDs injection, with imaging conducted at 50000× for the glomerulus (*n* = 14) and 12000× for the tubule (*n* = 40) in both ADN and 5/6Nx models. Scale bar, 2 µm (12000×; cell body), 1 µm (30000×; FT), 200 nm (80000×; FT or 100000×; Glom.Mito), and 500 nm (50000×; Glom. Mito). Three independent experiments with similar results were performed. Data is shown as the mean ± standard error of the mean. *p < 0.05, **p < 0.01, ***p < 0.001.

Finally, we examined changes in the microstructure and mitochondrial morphology in vivo using an electron microscope. Glomerular sclerosis and foot process diffuse effacement observed in the ADN and 5/6Nx models were ameliorated by GQDs treatment. Considering the glomerular filtration barrier, the slit pore diaphragm improved after GQDs treatment. The morphology of mitochondria in the glomerulus was also observed (**Figure** **8**A,B). Renal tubules in both models contained numerous elongated mitochondria with densely stacked cristae membranes after GQDs treatment. Additionally, the hyaline cast in the tubule, tubular dilatation, architecture collapse, and brush border loss were recovered by treatment with GQDs (Figure 8C,D). AD stimulation or 5/6Nx operation was accompanied by mitochondrial swelling, fragmentation, and aberrant cristae morphogenesis (fusion and loss). Following GQDs treatment, we observed an uptick in mitochondrial lengths, cristae area, and cristae volume density in both glomerulus and tubule sections (Figure 8E,F). This structural enhancement paralleled the observed boost in mitochondrial respiration and the elevated cristae density within the mitochondria.

### 2.6 GQDs Inhibit Fibroblast Activation and Protect Renal Cells from Damage during Fibrosis

The rTGF‐β‐induced expression of FN and TRPC5 were suppressed in a dose‐dependent manner after GQDs treatment in podocytes, hTECs, and NIH3T3 (**Figure** **9**A–C). Under oxidative stress conditions, the apoptotic cell count was reduced by more than half, whether hTECs were pre‐treated with GQDs or treated simultaneously. Moreover, cell viability was restored by GQDs treatment to a level comparable to that of the control. Even when treatment was initiated after 5 and 20 min, the effect was maintained and increased (Figure 9D). Collectively, GQDs alleviate renal fibrosis by inhibiting TRPC5 activation and enhancing cell viability.

Figure 9GQDs ameliorated kidney injury and fibrosis by suppressing fibroblast activation. A–C) Western blot analysis shows the effects of GQDs on human podocytes (A), hTECs (B), and NIH3T3 (C) after 48 h of incubation with recombinant transforming growth factor (rTGF‐β) (2 ng mL^−1^) and GQDs (0.25 and 0.5 µg mL^−1^) (*n* = 6 per group). D) hTECs are treated with H_2_O_2_ (0.5 mM) and GQDs (0.5 µg mL^−1^) for indicated periods (−1 h, 0, +5 min, +20 min). Annexin V/PI staining revealed the proportion of early apoptosis, late apoptosis, necrosis, and apoptotic cells. Flow cytometry density plot (top) and quantification (bottom) are shown (*n* = 5 per group). E) Summary diagram of the mechanism of the effects after GQDs treatment. Data are shown as the mean ± standard error of the mean. The experiments were repeated at least 3 times. *p < 0.05, **p < 0.01, ***p < 0.001.

**Figure:**
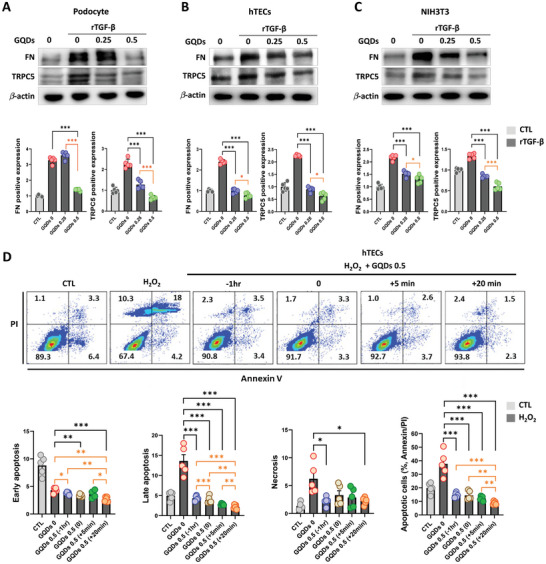

**Figure:**
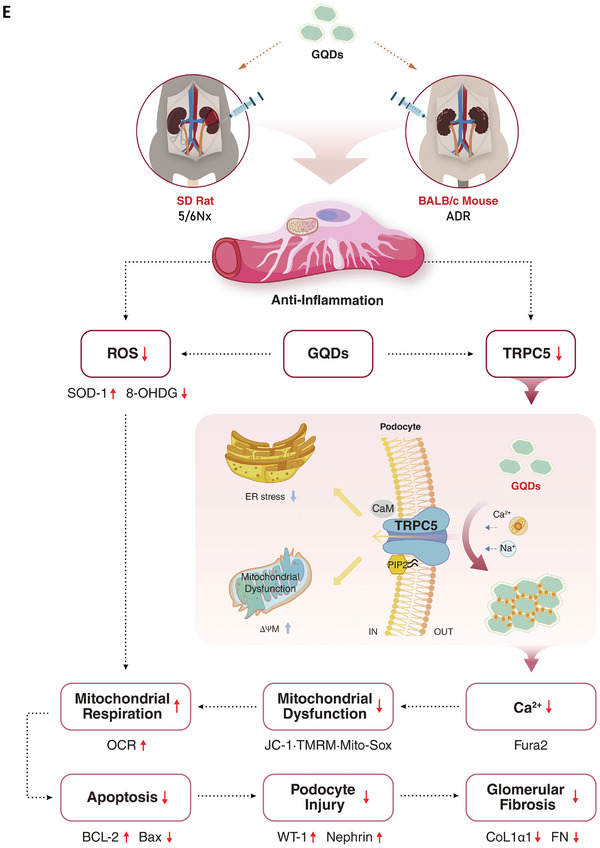

### 2.7 Pre‐ and Post‐Exposure with GQDs Effectively Delayed Kidney Fibrosis and Preserved Renal Cells in Hypertensive Conditions

As described earlier, our results show that GQDs attenuate kidney fibrosis and suppress TRPC5 activation. Drug administration schedules are critical and can alter effectiveness in certain disease models. In this model, we assessed the timing and efficacy of GQDs administration by comparing outcomes in pre‐treatment with post‐treatment (Figure 4A). Both kidney function and Cr clearance were significantly improved following GQDs treatment at both time points compared with the 5/6Nx alone (Figure S15A, Supporting Information). In kidney sections from the 5/6Nx group, collagen fiber deposition and TRPC5 expression decreased after GQDs treatment. Notably, the area positively stained for TRPC5 decreased more in the pre‐treatment group compared with the post‐treatment group (Figure S15B, Supporting Information).

Next, we induced hypertensive injury by applying a rotational force of 4 mmHg to human podocytes for 48 h and confirmed the therapeutic effects of pre‐treatment or post‐treatment with GQDs (Figure S15C, Supporting Information). As a result, the mRNA expression of podocyte cytoskeleton markers decreased, while FN expression increased. When GQDs were administered before the injury, the increase in WT‐1 and ZO‐1 expressions was more pronounced compared with post‐treatment, and FN expression decreased more significantly than post‐treatment. Additionally, the relative fluorescence of albumin‐rhodamine under hypertensive conditions was significantly reduced in the pre‐treatment group (Figure S15D, Supporting Information). Furthermore, the number of apoptotic and necrotic cells decreased proportionally to the dose, regardless of the timing of GQDs administration (Figure S15E, Supporting Information). This effect was more pronounced in the pre‐treatment group than in the post‐treatment group. Collectively, GQDs attenuate kidney injury and fibrosis by deactivating the TRPC5 ion channel, enhancing mitochondrial membrane potential in podocytes and preventing apoptosis (Figure 9E).

## 3 Discussion

To the best of our knowledge, this study presents the first comprehensive use of carbon‐based nanoparticles, specifically GQDs, in the ADN and 5/6Nx models. The findings demonstrated that GQDs significantly improved renal function and reduced proteinuria. Previous research has shown the efficacy of GQDs in preventing α‐synucleinopathy in Parkinson's disease^[^ ^3^ ^]^ and treating colitis through anti‐inflammatory mechanisms.^[^ ^16^ ^]^ However, their potential in kidney disease has not been explored to date. Before investigating the biological role of GQDs in kidney disease, we observed that Ca^2+^ ions bind to GQDs, resulting in a decrease in PL intensity and a blue shift in the maximum emission wavelength. Our findings revealed that GQDs exert anti‐inflammatory, anti‐apoptotic, and anti‐fibrotic effects in both in vivo and in vitro studies. These nanoparticles also mediate oxidative stress, aligning with the findings of Chong et al.^[^ ^17^ ^]^ GQDs are known to exhibit peroxidase‐like activity, catalyzing reactions with hydrogen peroxide (H_2_O_2_) due to their oxygen functional groups.^[^ ^18^ ^]^ Previous studies have highlighted that TRPC5 channel activation and the resultant intracellular Ca^2+^ influx are associated with podocyte injury in proteinuric kidney disease.^[^ ^19^ ^]^ This study reveals that GQDs administration promotes podocyte differentiation and restores podocyte morphology, cytoskeleton integrity, and function. Consistent with these results, transcriptomic analyses showed that GQDs treatment alters genes associated with Ca^2+^ transport and restores the expression of genes related to podocyte differentiation. Additionally, GQDs optimized mitochondrial function by increasing the levels of anti‐oxidative proteins and improving mitochondrial membrane potential. Moreover, the mitochondrial cristae architecture^[^ ^20^ ^]^ became more organized and denser. Collectively, these results have 3 important implications:

First, GQDs act as Ca^2+^‐binding agents,^[^ ^21^ ^]^ regulating intracellular Ca^2+^ influx and mediating the activation of Ca^2+^‐selective channels such as TRPC5. TRPC5, a novel target in podocyte disorders,^[^ ^10^ ^]^ when inhibited, stimulates the regeneration of the podocyte foot process and restores kidney filter function.^[^ ^22^ ^]^ However, no previous research has identified a potential candidate targeting Ca^2+^ signaling in podocytes via TRPC5, influencing mitochondrial respiration and cell viability.^[^ ^6^ ^]^ In the ADN mouse model, GQDs enhanced podocyte function and mitochondrial efficiency, offering a novel approach to addressing glomerular disorders. Furthermore, GQDs prevented kidney fibrosis and ameliorated hypertension under a 5/6Nx rat model. GQDs might successfully mediate high blood pressure by regulating TRPC5 activity with low cytotoxicity after long‐term administration. This indicates that GQDs have a role like that of Ca^2+^‐binding proteins and that their anti‐ROS properties may reduce podocyte hypertrophy.^[^ ^23^ ^]^ This aligns with the findings of several studies showing that TRPC5 is pathogenic in proteinuric kidney disease by contributing to Ca^2+^ entry and consequent podocyte injury.^[^ ^24^ ^]^

Second, GQDs restore mitochondrial membrane potential and increase kidney cell viability. Mitochondrial dysfunction plays an important role in various kidney diseases,^[^ ^25^ ^]^ and studies related to mitochondrial‐targeted therapeutics to protect and improve its function are being actively conducted.^[^ ^26^ ^]^ In this study, mitochondrial morphological abnormalities, such as swelling, fragmentation, and aberrant cristae morphogenesis, were resolved after GQDs administration.^[^ ^27^ ^]^ Additionally, intracellular Ca^2+^ influx decreased and increased cytosolic Ca^2+^ permeability was restored. The improvements in mitochondrial respiration are associated with changes in TRPC5 expression, with GQDs administration accompanied by a decrease in TRPC5 expression.

Third, the effect of GQDs was significant in both pre‐treatment and post‐treatment groups. Even under oxidative stress, this effect was observed when GQDs were administered simultaneously. These findings support further studies on different administration methods and dosing frequencies. We determined optimal doses of GQDs in both in vitro and in vivo models. GQDs have unique properties, such as high biocompatibility and a size of less than 100 nm,^[^ ^28^ ^]^ allowing them to penetrate the kidney without disrupting molecular structures and exhibiting low cytotoxicity. Interestingly, in vitro treatment with low‐dose GQDs demonstrated sufficient improvement in mitochondrial respiration rate and rescue of renal cells.

In addition to these compelling findings, our results demonstrate that GQDs not only affect podocytes but also exhibit remarkable efficacy in fibroblasts and hTECs, highlighted by the notable decrease in TRPC5 protein expression. This is significant for addressing kidney fibrosis, as the fibroblast‐to‐myofibroblast transition exacerbates fibrosis and induces changes in renal cells.^[^ ^29^ ^]^

This study has some limitations. The first drawback of this study is the lack of comparability with previous, related work. To address concerns regarding the relative efficacy of GQDs treatment compared to current standard drugs, we used calcium channel blocker (CCB; amlodipine)^[^ ^30^ ^]^ and vitamin C. Comparable to that in the CCB or vitamin C‐treated group, there was a marked decrease in BUN/Cr, NGAL, FN, F4/80, and cytochrome C, and an increase of SOD‐1 and WT‐1 in the GQDs‐treated group (Figure S16, Supporting Information). However, the expression of TRPC5 showed a different direction from that of GQDs, in the CCB‐treated group. This suggests that the mechanism by which CCB exerts its effect is independent of TRPC5, which is different from the mechanism of GQDs. In the experiments on primary cultured human podocytes (Figure S17A, Supporting Information), both GQDs, CCB, and vitamin C treatments restored the podocyte injury and decreased FN and TRPC5 levels, while simultaneously increasing WT‐1 and ZO‐1 expression (Figure S17C–E, Supporting Information). Notably, GQDs demonstrated a more potent reduction in FN expression than CCB, highlighting their potential superiority in anti‐fibrotic effects. Furthermore, GQDs targeted TRPC5 over CCB, underscoring their potential as a novel nanomedicine addressing TRPC5 overexpression, a key contributor to podocyte‐related kidney injuries. Furthermore, the hydroxyl radical scavenging rate also increased significantly in the GQDs treatment groups, comparable to that in the CCB or vitamin C‐treated group (Figure S18, Supporting Information). These results suggest that GQDs can mitigate kidney fibrosis through hydroxyl radical scavenging.

Second, information on the long‐term effects, toxicity, or potential off‐target effects of GQD treatment was not reported. However, in the MTS assay using 2 cell types, both cell types maintained their viability across GQDs concentrations (0.125, 0.25, and 0.5 µg mL^−1^) (Figure S17B, Supporting Information), suggesting the optimal doses of GQDs used in this study and safety of GQDs application in kidney cells.

In animal studies, especially in the 5/6Nx model, GQDs were injected several times, suggesting a need to optimize the dosing interval or reduce the dose. Changes in the early or acute phase were not observed, indicating the need for further studies to confirm time‐sequential changes, including the initial response. In mRNA‐seq analysis, DEGs for TRPC5 were either not detected or had weak signals. TRPC5 in podocytes interacts with signals from various channels, such as G protein‐coupled receptors, including angiotensin II receptor type 1 and receptor tyrosine kinases.^[^ ^31^ ^]^ Based on our GO enrichment analysis, we expected GQDs to suppress the *Trpv5* gene, which encodes a Ca^2+^‐sensitive channel with different N‐terminal and C‐terminal regions compared with the TRPC family.^[^ ^32^ ^]^ mRNA‐sequencing further revealed that GQDs mediates the expression of S100A8 (713.71‐fold reduction) and S100A9 (226.83‐fold reduction), collectively known as calprotectin. The impaired activity of TRPC5 was linked to a reduction in calprotectin levels, likely because calprotectin interacts with Toll‐like receptor 4,^[^ ^33^ ^]^ which in turn stimulates diacylglycerol production.^[^ ^34^ ^]^ Whether other mechanisms contribute to TRPC5 hyperactivation after AD injection remains to be established and requires further study. Lastly, more evidence is required to understand how GQDs modulate TRPC5 activation and Ca^2+^‐signaling‐induced mitochondrial dysfunction through proteome and metabolome analyses.

## 4 Conclusion

This study demonstrated a novel application for GQDs in treating kidney disease. Our findings suggest that GQDs can mitigate oxidative stress‐induced mitochondrial damage, inflammation, apoptosis, and fibrosis. Specifically, we explored the potential of GQDs as a nanomaterial to target the calcium transporter TRPC5. This appears to be mediated by the calcium‐binding ability of GQDs attributed to their negatively charged surfaces and subsequent effects on reduction in intracellular Ca^2^⁺ influx. In the future, follow‐up studies are necessary to prove long‐term effects and safety and to prove their efficacy and effectiveness through large‐animal experiments. Additional research on dosage, formulation, and usage is necessary when administering them to actual patients. We believe GQDs hold potential as an intervention for various kidney diseases by mitigating the onset and progression of fibrosis.

## 5 Experimental Section

### Synthesis of GQDs

GQDs were synthesized via thermo‐oxidative cleavage in a strong acid solution, in which 0.9 g of carbon fibers were heated in a mixture of nitric and sulfuric acid at 80–100 °C for 24 h. The solution was then dialyzed, vacuum‐filtered to remove large particles, and subjected to rotary evaporation to obtain the GQDs in powder form. The final product was stored in a desiccator for further analysis.

### Characterization of GQDs

For transmission electron microscopy (TEM) imaging, a GQDs solution (10 µg mL^−1^) was applied to 300 mesh lacey carbon‐coated TEM grids and analyzed using high‐resolution TEM (HR‐TEM) and spherical aberration‐corrected transmission electron microscopy (Cs‐TEM) (Figure S1, Supporting Information). For Raman spectroscopy, 10 mg of GQDs powder was placed on a silicon substrate and measured with an Ar laser‐based Raman spectrometer. Additionally, the GQDs were characterized by FT‐IR spectroscopy using the KBr pellet method and X‐ray photoelectron spectroscopy (XPS) after drying the samples on a SiO_2_ wafer.

### Zeta Potential of GQDs

For zeta potential analysis, a 50 µg mL^−1^ GQDs solution was prepared in DI water and PBS (pH 7.4, 10 mM). The GQDs solution was also mixed with 10 mM of different salt solutions, NaCl, KCl, MgCl_2,_ and CaCl_2_ (Table S1, Supporting Information), for analyzing zeta potential changes in response to each cation. The Zeta potential of each GQDs solution was then measured using Zetasizer Nano ZS (Malvern Instruments Ltd, Malvern, UK).

### Photoluminescence of GQDs Under Ionic Solution

For photoluminescence (PL) analysis, a 0.1 mg mL^−1^ GQDs solution was sonicated and measured using a spectrofluorometer with 365 nm excitation. CaSO_4_ solutions (Table S1, Supporting Information) at varying concentrations were mixed with the GQDs, and the PL emission was measured; a trend line was then plotted, and the coefficient of determination (R^2^) was calculated. Also, 200 µM EDTA solution was further added to 400 µM calcium‐GQDs solution to observe recovery of PL, and 1 mM of different salt solutions (NaCl, KCl, MgCl_2,_ and CaCl_2_) were mixed with GQDs solution for comparing the PL quenching effect of each cation.

### Animals

All animal studies were reviewed and approved by the Institutional Animal Care and Use Committee (IACUC) of the Clinical Research Institute of Seoul National University Hospital: ADN mouse model (N. 21‐0282‐S1A0(2)) and 5/6Nx rat model (N. 20‐0009‐S1A0(1)). All animals were maintained at 22 °C and 50%–60% humidity under a 12 h light/dark cycle.

### In Vivo Models

For ADN experiments, eight‐week‐old male BALB/c mice (KOATECH, South Korea) were intravenously injected with 11.5 mg kg^−1^ of Adriamycin (AD; TCI, Tokyo, Japan, Table S2, Supporting Information). AD‐treated mice received GQDs (10 or 20 mg kg^−1^; Graphene Square, South Korea) intraperitoneally 4 times at 2‐day intervals (day 0, 2, 4, and 6). To evaluate the therapeutic effects of GQDs in comparison to standard agents, vitamin C (VitC) (200 mg kg^−1^, I.P.) and the calcium channel blocker (CCB) amlodipine (10 mg kg^−1^, P.O.) were administered daily for up to 7 days (Table S2, Supporting Information). The kidneys and spleen of mice were extracted after 7 days. For 5/6Nx experiments, eight‐week‐old male Sprague‐Dawley rats underwent a ventral midline incision to expose and partially constrict the left kidney, with the right kidney excised a week later. GQDs (4 mg kg^−1^) were administered intraperitoneally every 2 days either starting 2 weeks before the secondary surgery (pre‐treatment group) or from week 2 to 8 post‐nephrectomy (post‐treatment group). All rats were sacrificed at 8 weeks after the 5/6Nx surgery.

### In Vitro Model and Treatments

Human podocytes and human tubular epithelial cells (hTECs) were isolated and cultured according to a previously described protocol^[^ ^35^ ^]^ (Table S3, Supporting Information). Isolated podocytes were cultured in DMEM/F12 (Biowest, Nuaille, France, Table S4, Supporting Information), supplemented with 20% fetal bovine serum (FBS, Gibco, Grand Island, NY, USA), 1× insulin‐transferrin‐selenium (Gibco), 200 µM L‐glutamine (Gibco), 1% penicillin/streptomycin (Gibco), and 50 nM hydrocortisone (Sigma‐Aldrich, St. Louis, MO, USA). Next, the hTECs were cultured in REGM (Lonza, Switzerland), while HK‐2 cells (ATCC: CRL‐2190) were cultured in DMEM/F12 (Biowest) supplemented with 10% FBS (Gibco) and 1% penicillin/streptomycin (Gibco). For in vitro stimulation under H_2_O_2_ (0.5 or 1 mM; Supelco, Bellefonte, PA, Table S4, Supporting Information) and recombinant TGF‐β (2 ng mL^−1^; R&D systems, Minneapolis, MN, Table S4, Supporting Information), cells were treated with GQDs (0.25 or 0.5 µg mL^−1^), VitC (0.5 or 1 mM), and CCB (0.01 or 1 µM) in a 2% FBS‐containing medium. Additionally, to stimulate the ADN in vitro model, podocytes were co‐treated with Adriamycin (10 ng mL^−1^) and GQDs (0.25 or 0.5 µg mL^−1^). NIH3T3 (ATCC: CRL‐1658) mouse fibroblasts were cultured in DMEM high glucose media supplemented with 10% FBS and 1% streptomycin. NIH3T3 fibroblasts were exposed to recombinant mouse TGF‐β (2 ng mL^−1^; R&D systems, Table S4, Supporting Information) and GQDs (0.25 or 0.5 µg mL^−1^) for 48 h.

### Assessment of Renal Function

After 24 h urine collection, urine protein and creatinine (Cr) concentrations were measured, and the urine protein/Cr ratio (mg mg^−1^) was calculated. Blood urea nitrogen (BUN) (mg dL^−1^) and serum Cr (mg dL^−1^) concentrations were determined by measuring the rate of the modified Jaffe reaction, and lactate dehydrogenase (LDH) (U L^−1^) levels were assessed in plasma 7 days post‐AD injection to evaluate kidney infection and injury. All measurements were performed using an autoanalyzer (Hitachi Chemical Industries, Japan). Cr clearance was calculated as described in the Supplementary methods. Blood pressure in the 5/6Nx model was measured using the tail‐cuff method (Kent Scientific Corporation, USA).

### Histologic Analysis

Kidney tissues were fixed in 10% formalin embedded overnight in paraffin and sectioned in 4 µm thickness. Sections were then stained with primary antibodies (Table S5, Supporting Information). Masson's trichrome (MT) and periodic Acid‐Schiff (PAS) staining were used to assess kidney fibrosis and glomerular damage. Sirius red staining (Abcam, Cambridge, UK, Table S6, Supporting Information) was used to measure collagen accumulation during kidney fibrosis. Glomerular sections were scored according to the glomerular sclerosis index.^[^ ^36^ ^]^ Stained slides were imaged using a Leica inverted microscope (Leica Camera, Wetzlar, Germany). The LAS‐4000 program (Leica Camera) was used to quantify the positive areas (%).

### Western Blotting

Total kidney proteins were isolated using RIPA buffer (150 mM NaCl; 100 mM Na3VO4; 50 mM Tris; HCL, pH 7.3; 0.1 mM EDTA 1% (vol/vol) sodium deoxycholate; 1% (vol/vol) Triton X‐100; and 0.2% NaF; Biosesang, South Korea) with a protease inhibitor (GenDEPOT, Altair, TX, USA). Protein extracts were electrophoresed using glycine‐sodium dodecyl sulfate buffer and transferred to polyvinylidene difluoride membranes (Amersham plc, Amersham, UK) on ice. The membranes were blocked for 1 h using a blocking solution (5% skimmed milk; Biosesang). Subsequently, the membranes were incubated with appropriate primary antibodies overnight at 4 °C with shaking (Table S5, Supporting Information). After incubation with horseradish peroxidase‐conjugated secondary antibodies (Cell Signaling Technology, Danvers, MA, USA, Table S7, Supporting Information), membranes were visualized using ImageQuantTM LAS 4000 mini (Amersham plc) with optimal exposure time. Densitometry was performed using the gel analysis procedure in ImageJ (National Institutes of Health, Bethesda, MD, USA).

### Quantitative Real‐Time Reverse Transcription PCR (RT‐qPCR)

Total RNA was extracted using TRIzol RNA isolation reagent (Invitrogen, Waltham, MA, USA). cDNA synthesis was performed using AMV Reverse Transcriptase (Promega, Madison, WI, USA, TableS8, Supporting Information) and a C1000 Touch Thermal Cycler (Bio‐Rad, Hercules, CA, USA). SYBR‐green dye‐based RT‐qPCR was performed using a 7500 Real‐time PCR system (Applied Biosystems, Foster City, CA, USA).^[^ ^37^ ^]^ mRNA expression was calculated using the comparative Ct method (2^−ΔΔCt^) after normalization to glyceraldehyde 3‐phosphate dehydrogenase (*GAPDH*) or 𝛽‐actin. The primer sequences are listed in Table S9 (Supporting Information).

### Confocal Microscopic Examination

4′‐6‐Diamidino‐2‐phenylindole (DAPI) (Thermo Fisher Scientific) was used to stain nuclei, with excitation provided by a 405 nm violet laser. Podocytes‐specific primary antibodies were employed (Tables S5 and S10, Supporting Information), and Alexa Fluor 488‐, 555‐, and 647‐conjugated secondary antibodies (Invitrogen, Table S11, Supporting Information) were excited using an argon 488 laser, resulting in emission spectra for green, red, and deep red or magenta fluorescence. The signals were amplified using a photomultiplier detector, and DAPI's electromagnetic spectrum was detected using a hybrid sensor. Confocal microscopy was performed with a scanner set to 400 Hz, a 4x line average, and an image pixel size of 1024×1024 in a sequential scanning mode of the LAS‐X program (Leica).

Human podocytes were cultured on coverslips for 2 days and stained with MitoTracker (50 nM; Invitrogen, Table S12, Supporting Information) following a standard protocol. After washing with PBS, the cells were fixed with 4% paraformaldehyde for 15 min, followed by additional washes and treatment with Triton X‐100 and 10% normal goat serum. Confocal microscopy was then performed using a Leica DMI 6000 inverted microscope equipped with a TCS SP8 STED CW system and a 20×/0.7 NA objective lens.

### Flow Cytometry Analysis

FACS experiments assessed phenotypic changes in CD3^+^ kidney‐infiltrating T lymphocytes and CD11b^+^ macrophages following GQDs treatment in the ADN mouse model. Kidney tissues were harvested one week after AD administration, and single cells were homogenized and isolated using a Percoll gradient. The cells were then stained with fluorescence‐conjugated antibodies specific to T‐cell and macrophage markers (Table S13, Supporting Information) and analyzed using a BD Canto flow cytometer, with data processed in FlowJo (version 10.0.7; FlowJo LLC, Ashland, OR, USA).

### Annexin V/PI Staining

The percentage of apoptotic and necrotic cells was measured using an Annexin V/PI FITC apoptosis kit (BD Biosciences, Table S14, Supporting Information). Podocytes were stained with Annexin V and PI, incubated for 15 min at 25 °C in the dark, and analyzed using BD FACSDiva software (version 8.0; BD Biosciences).

### Intracellular Calcium Measurements

Ca^2+^ fluctuations were monitored using the Fura‐2AM Ca^2+^ influx assay kit (Abcam, Table S15, Supporting Information) following the manufacturer's protocol. Cells were seeded in a 96‐well plate and treated with ionomycin (Sigma‐Aldrich, Table S15, Supporting Information) and GQDs for 1 h in DMEM/F12 medium. Before measurement, cells were incubated with a Fura‐2AM and Pluronic mixture, and the microplate was read at Ex/Em 340/510 nm and Ex/Em 380/510 nm using an Operetta CLS (PerkinElmer, Waltham, MA, USA).

### Mitochondrial Membrane Potential

Mitochondrial voltage potential and ATP yield, closely tied to oxidative phosphorylation, were assessed in human primary cultured podocytes and hTECs by staining with MitoTracker Green (100 nM, Invitrogen, Table S12, Supporting Information) and tetramethylrhodamine, ethyl ester, and perchlorate (TMRM; 100 nM, Invitrogen, Table S12, Supporting Information) for 15 min. Fluorescence was excited at 488 and 548 nm to observe mitochondrial membrane potential under oxidative stress. To detect mitochondrial reactive oxygen species (ROS) production, cells were further incubated with MitoSOX Red (5 µM, Invitrogen, Table S12, Supporting Information), followed by counterstaining with DAPI (Invitrogen, Table S10, Supporting Information), and images were captured using an Operetta CLS (PerkinElmer).

### Mitochondrial Respiratory Assay

Mitochondrial respiration parameters, including basal, ATP production‐linked, maximal, and proton leak‐linked OCR, were measured using the Seahorse XF ATP Real‐Time rate assay (Agilent Technologies, Santa Clara, CA, USA, Table S16, Supporting Information). Podocytes treated with H_2_O_2_ (0.5 mM) and GQDs (0.25 or 0.5 µg mL^−1^) were seeded onto a Seahorse plate, incubated for 2 h, washed, and resuspended in Seahorse XF medium before a final incubation and centrifugation. Data were analyzed using the Seahorse Wave software (Agilent Technologies).

### JC‐1 Assay

To analyze mitochondrial membrane potential under oxidative stress induced by H_2_O_2_, JC‐1 fluorescence detection was performed using the MitoProbe JC‐1 Assay kit (Invitrogen, Table S16, Supporting Information) with modifications. Samples, including 2 positive controls (50 mM CCCP and 1 mM H_2_O_2_), were incubated with JC‐1 (10 µL of 200 µM) for 30 min, and fluorescence was detected at 488 and 633 nm using a BD FACS Canto (FACSDiva version 80).

### Permeability Assays in Human Podocytes

The permeability of slit diaphragms in the glomerular filtration barrier is regulated by slit diaphragm proteins and podocyte‐related proteins.^[^ ^38^ ^]^ The rate of albumin transport across human podocytes in a Transwell system was measured using an albumin‐rhodamine transit assay in 24‐well plates with 0.4 µm pore membranes. DMEM/F12 containing albumin‐rhodamine (Abcam, Table S17, Supporting Information) was added to a monolayer of podocytes, and fluorescence intensity was quantified after 24 h of treatment with AD (2 ng mL^−1^), and GQDs (0.25 or 0.5 µg mL^−1^) under fibrotic conditions induced by rotational force. A standard curve was used to determine the albumin‐rhodamine concentration at Ex/Em 550/570 nm using a spectrophotometer.

### Induction of Fibrosis with Mechanical Stress Device

Our previous report demonstrated that hypertensive‐mimicking devices effectively induce endothelial dysfunction in response to podocyte injury in hypertensive nephrosclerosis patients.^[^ ^39^ ^]^ In this study, human primary podocytes, with or without GQDs, were cultured in 6‐well plates and subjected to mechanical stress using a rotational force device (4 mmHg) for 48 h. Podocytes were either pre‐treated with GQDs (0.5 µg mL^−1^) 3 h before device placement or post‐treated 6 h after, and subsequent molecular analyses were conducted to assess oxidative stress and apoptosis.

### ROS Assay

Intracellular ROS levels were evaluated by monitoring the increase in DCF fluorescence intensity. Human podocytes treated with H_2_O_2_ (1 mM) and GQDs (0.25 or 0.5 µg mL^−1^) were incubated with DCFH‐DA (Cell Biolabs, San Diego, CA, USA, Table S17, Supporting Information) for 1 h at 37 °C. The resulting fluorescence intensity was measured to determine ROS levels using a fluorescence spectrometer (Tecan, Research Triangle Park, NC, USA).

### β‐galactosidase Staining and Wound Healing Scratch Assay

Senescence β‐Galactosidase Staining Kit (Cell Signaling, Table S18, Supporting Information) was used to detect cellular senescence and evaluate the therapeutic effect of GQDs under ROS‐induced stress, with imaging performed via Leica inverted microscope (Leica camera). Live/Dead assay (Invitrogen, Table S18, Supporting Information) was used to determine the number of dead cells following exposure to H_2_O_2_. Wound healing ability was evaluated using the Operetta CLS system (PerkinElmer) and analyzed with digital phase contrast (DPC).

### Singleplex Protein Analysis

The concentration of IL‐8 in cell culture media was measured using a Bio‐plex 200 system (Bio‐Rad) according to the manufacturer's protocol.

### Electron Microscopy

Kidney sections were fixed, post‐fixed, and embedded before TEM sections were stained with lead citrate and uranyl acetate, then were examined using a JEM‐1400 electron microscope (JEOL Ltd., Akishima, Tokyo, Japan). Random areas from 1–2 glomeruli per animal were photographed at magnifications of 12000× and 30000×, with additional images captured at 80000× to study podocyte foot processes and at 50000× and 100000× for mitochondrial analysis in glomeruli and tubules, respectively. Mitochondrial lengths, cristae area, and volume density were quantitatively assessed using ImageJ software.^[^ ^40^ ^]^

### Hydroxyl Radical Antioxidant Capacity (HORAC) Assay

The antioxidant capacity of GQDs was evaluated in comparison to established antioxidants, specifically ascorbic acid (Sigma‐Aldrich, Table S2, Supporting Information) and amlodipine (Sigma‐Aldrich, Table S2, Supporting Information), utilizing the OxiSelect HORAC Activity Assay (Cell Biolabs, Table S19, Supporting Information) according to the provided protocol (Table S19, Supporting Information). Briefly, gallic acid standards and podocyte lysates were diluted and mixed with 1X fluorescein solution in a 96‐well microtiter plate. Subsequently, a hydroxyl radical initiator and a Fenton reagent were added to initiate the reaction. The fluorescence intensity was monitored using BioTek Synergy Neo2 (Agilent Technologies) at an excitation wavelength of 480 nm and an emission wavelength of 530 nm in increments of 1 min over a total duration of 60 min.

### MTS Assay

HK‐2 cells and podocytes were treated with GQDs at concentrations of 0, 0.125, 0.25, 0.5, 1, and 2 µg mL^−1^ to assess cell viability using a colorimetric MTS cell proliferation assay kit (DoGenBio, Table S17, Supporting Information). Following the manufacturer's protocol, cells were incubated with MTS reagents for 1 h at 37 °C. Absorbance was then measured at 450 nm using the Versamax microplate reader (Molecular Devices, CA, USA).

### mRNA‐Sequencing Analysis

Total kidney RNA was extracted using TRIzol reagent (Invitrogen), and RNA quality was assessed with an Agilent 2100 Bioanalyzer (Agilent Technologies). RNA sequencing and data analysis were performed by EBIOGEN, Inc. (Seoul, South Korea) with high‐throughput sequencing conducted using HiSeq X10 (Illumina, San Diego, CA, USA). Differentially expressed genes (DEGs) were analyzed using the ExDEGA package (Ebiogen), with Gene Ontology (GO) and KEGG pathway enrichment analyses (Tables S20–S22, Supporting Information) conducted in R, and network analysis performed using Cytoscape (version 3.10.0).

### Statistical Analyses

All data are expressed as the mean ± standard error of the mean. Two groups were compared using a two‐tailed unpaired Student's t‐test, conducted with GraphPad Prism 10.2.3 (GraphPad, USA). The R packages of enrichGO and enrichKEGG were used to calculate p‐values for mRNA‐seq analysis. A one‐sample t‐test was performed using R, and the false discovery rate (FDR) value was calculated using the following equation: [p‐value (one‐sample t‐test)] × [(total number of ranks) / (number of ranks)]. The correlation coefficient (r) indicates the relationship between specified targets and TRPC5. For all statistical tests, p‐values of <0.05 were considered significant.

### Detailed Methodology

Detailed methodology is described in the supporting information.

### Ethics Approval Statement

All animal studies were reviewed and approved by the Institutional Animal Care and Use Committee (IACUC) of the Clinical Research Institute of Seoul National University Hospital: ADN mouse model (IACUC: 21‐0282‐S1A0(2)) and 5/6Nx model rat model (IACUC: 20‐0009‐S1A0(1)). Human biopsy kidney was collected with approval from the Institutional Review Board of Seoul National University Hospital (approval number: 2203‐053‐1303), and informed consent was obtained from all patients.

## Conflict of Interest

The authors declare that they have no competing interests.

## Author Contributions

K.H.K., J.B.P., and J.N.A. contributed equally to this work. K.H.K., J.B.P., J.N.A., B.H.H., Y.S.K., and S.H.Y. performed conceptualization. S.H.Y. performed funding acquisition. K.H.K., G.B., Y.J., and S.H.Y. performed investigation. K.H.K., K.H.K., G.B., S.J.P., Y.J., and S.H.Y. performed methodology. B.H.H. and S.H.Y. performed project administration. J.B.P. and B.H.H. brought resources. Y.C.K., J.P.L., J.W.L., D.K.K., Y.S.K., B.H.H., and S.H.Y. performed supervision. K.H.K., J.N.A., and S.H.Y. performed visualization. K.H.K., J.N.A. wrote the original draft. J.B.P., Y.S.K., B.H.H., and S.H.Y. wrote, reviewed and edited the original draft.

## Supporting information



Supporting Information

## Data Availability

The data that support the findings of this study are available in the supplementary material of this article.
