# Supplementary material for: Effects of Graphene Quantum Dots on Renal Fibrosis Through Alleviating Oxidative Stress and Restoring Mitochondrial Membrane Potential
Source: Adv Sci (Weinh). 2024 Dec 30;12(10):2410747. doi: 10.1002/advs.202410747 (PMC11904958; doi:10.1002/advs.202410747)
Supplement: Supplementary file 1 — Supporting Information [file ADVS-12-2410747-s001.pdf]

ADVANCED SCIENCE
Open Access

Supporting Information

for *Adv. Sci.*, DOI 10.1002/advs.202410747

Effects of Graphene Quantum Dots on Renal Fibrosis Through Alleviating Oxidative Stress and Restoring Mitochondrial Membrane Potential

Kyu Hong Kim, Jong Bo Park, Jung Nam An, Gaeun Bae, Kyu Hyeon Kim, Seong Joon Park, Youngjin Jung, Yong Chul Kim, Jung Pyo Lee, Jae Wook Lee, Dong Ki Kim, Yon Su Kim, Byung Hee Hong* and Seung Hee Yang**

Supporting Information

Title: Effects of Graphene Quantum Dots on Renal Fibrosis Through Alleviating Oxidative Stress and Restoring Mitochondrial Membrane Potential

Kyu Hong Kim, BS, Jong Bo Park, PhD, Jung Nam An, MD, PhD, Gaeun Bae, BS, Kyu Hyeon Kim, BS, Seong Joon Park, BS, Youngjin Jung, PhD, Yong Chul Kim, MD, PhD, Jung Pyo Lee, MD, PhD, Jae Wook Lee, MD, PhD, Dong Ki Kim, MD, PhD, Yon Su Kim, MD, PhD, Byung Hee Hong, PhD* and Seung Hee Yang, PhD**

2. Detailed Methodology

2.1 Synthesis of GQDs

GQDs were synthesized through a thermo-oxidative cleavage reaction in an acid solution. Briefly, 0.9 g carbon fibers were added to a mixture of strong acid (100 mL nitric acid and 300 mL sulfuric acid; Samchun Chemical, South Korea), followed by heating at 80–100°C for 24 h. The solution was dialyzed in tertiary deionized water to remove acid residues and then vacuum-filtered through an inorganic membrane filter with 20-nm-sized pores (Whatman-Anodisc 47; GE Healthcare, Chicago, IL, USA) to remove undesirable large particles. Subsequently, the GQDs solution was subjected to rotary evaporation to yield the final product in powder form. The samples were placed in a desiccator for further analysis.

2.2 Characterization of GQDs

For transmission electron microscopy (TEM) imaging, the GQDs solution (10 µg/mL) was added to 300 mech lacey carbon-coated TEM grids (Ted Pella Inc., Redding, CA, USA) (**Figure S1).** The samples were analyzed using a high-resolution TEM (HR-TEM, JEM-3010, JEOL, Ltd.) with a Gatan digital camera (MSC-794, Gatan, Pleasanton, CA, USA) at 300 kV and TEM with a spherical aberration corrector (Cs-TEM, JEM-ARM200F, JEOL, Ltd.) at 80 kV. For Raman spectrum, 10 mg GQDs powder was prepared on a silicon substrate and measured with an Ar laser (514 nm)-based Raman spectrometer (RENISHAW, Wotton-under-

Edge, UK). For Fourier Transform Infrared (FT-IR) spectroscopy, the powder samples were prepared by conventional KBr pellet method and measured with a vacuum FT-IR spectrometer (Vertex-80V, Bruker, Billerica, MA, USA). For X-ray photoelectron spectroscopy (XPS), the samples were dried on SiO_2 wafer and measured by XPS (AXIS-HSi, KRATOS, Manchester, UK).

2.3 Zeta potential of GQDs

For zeta potential analysis, a 50 μg/mL GQDs solution was prepared in DI water and PBS (pH 7.4, 10 mM). GQDs solution was also mixed with 10 mM of different salt solutions, NaCl, KCl, MgCl2 and CaCl2 (**Table S1**), for analyzing zeta potential changes in response to each cation. Zeta potential of each GQDs solution was then measured using Zetasizer Nano ZS (Malvern Instruments Ltd, Malvern, UK).

2.4 Photoluminescence of GQDs under Calcium Ions

For photoluminescence (PL) analysis, a 0.1 mg/mL GQDs solution was sonicated for 10 min and then measured using a spectrofluorometer (FP-8300, Jasco, Tokyo, Japan) with 350 nm excitation. $CaSO_4$ solutions (**Table S1**) at varying concentrations (40–400 μM) were prepared and mixed with the GQDs solutions by sonication for 5 min, followed by measuring PL emission of GQDs (n = 3) under 350 nm excitation. After measuring PL with different Ca^{2+} concentrations, a trend line was plotted, and the coefficient of determination (R^2) was calculated.

2.5 Animal Models

All animal studies were reviewed and approved by the Institutional Animal Care and Use Committee (IACUC) of the Clinical Research Institute of Seoul National University Hospital: ADR mouse model (N. 21-0282-S1A0(2)) and 5/6Nx rat model (N. 20-0009-S1A0(1)). All animals were maintained at 22°C and 50–60% humidity under a 12 h light/dark cycle.

2.6 Establishment of the Adriamycin-induced Nephropathy Mouse Model

Eight-week-old male BALB/c mice (KOATECH, South Korea) were intravenously injected

with 11.5 mg kg^{-1} of Adriamycin (AD; TCI, Tokyo, Japan, **Table S2**). AD-treated mice received GQDs (10 or 20 mg kg^{-1}; Graphene Square, South Korea) intraperitoneally four times at 2-day intervals (days 0, 2, 4, and 6). To evaluate the therapeutic effects of GQDs in comparison to standard agents, vitamin C (VitC, 200 mg kg^{-1}, I.P.) and amlodipine (Calcium Channel Blocker; CCB, 10 mg kg^{-1}, P.O.) were administered daily for up to 7 days (**Table S2**). The kidneys and spleens of mice were extracted after 7 days.

2.7 Establishment of the 5/6 Subtotal Nephrectomy Rat Model

Eight-week-old male Sprague-Dawley rats underwent a ventral midline incision to the abdomen to expose the left kidney. In the sham group, the left kidney was briefly relocated within the core. The upper and lower poles of the left kidney were tied with 3-0 sutures to ensure that the diameter was approximately half the size of the kidney. The right kidney was exercised 1 week after the first surgery, and the renal blood vessel was ligated. GQDs (4 mg kg^{-1}) were administered intraperitoneally beginning 2 weeks before the second surgery and continued at 2-day intervals until sacrifice during week 8, forming the pre-treatment group. Conversely, the post-treatment group received GQDs (4 mg kg^{-1}) every 2 days from weeks 2 to 8 following the right nephrectomy.

2.8 In vitro Model of ADN and 5/6Nx

Human podocytes and tubular epithelial cells were isolated and cultured according to a previously described protocol [1]. Kidney tissue was obtained from surgical specimens of patients with renal cell carcinoma. This study was approved by the Institutional Review Board of Seoul National University Hospital (approval number: 2203-053-1303), and informed consent was obtained from all patients. Kidney tissues were smashed using a stainless-steel mesh and cultured for 8 days. The outgrowing total renal cells were trypsinized and passed through sieves with a 50 to 70 μm pore size to isolate human podocytes and tubular epithelial cells. Fc receptor blocking reagent (1 μg mL^{-1}, BD Biosciences, Franklin Lakes, NJ, USA) was added to 1×10^6 cells. Podocyte-specific biomarkers, including anti-podocalyxin (Fischer Scientific, Waltham, MA, USA, **Table S3**) was used to identify podocytes. The cells were sorted and analyzed using the FACS Calibur Flow Cytometer (BD Biosciences). (95% purification) In the case of human tubular epithelial cells (hTECs), aquaporin-1 (R&D systems,

Minneapolis, MN, USA, **Table S3**) antibodies were used for flow cytometry. Isolated tubular cells were cultured in a renal epithelial cell growth medium (Lonza, Basel, Switzerland, **Table S4**). Dulbecco's modified Eagle's medium DMEM)/F12 (Biowest, Nuaille, France, **Table S4**) supplemented with 20% fetal bovine serum (FBS, Gibco, Grand Island, NY, USA), 1× insulin-transferrin-selenium (Gibco), 200-μM L-glutamine (Gibco), 1% penicillin/streptomycin (Gibco), and 50 nM hydrocortisone (Sigma-Aldrich, St. Louis, MO, USA) was used as the podocyte-selective medium. HK-2 cells (ATCC, CRL-2190; Manassas, VA, USA) were cultured in DMEM/F12 (Biowest) supplemented with 10% FBS (Gibco) and 1% penicillin/streptomycin (Gibco). To maximize renal cell growth, the culture plate was coated with 10 g mL^{-1} fibronectin (Sigma-Aldrich). For *in vitro* stimulation under H_2O_2 (0.5 or 1 mM; Supelco, Bellefonte, PA, **Table S4**) and recombinant human TGF-β (2 ng mL^{-1}; R&D systems, **Table S4**), cells were treated with GQDs (0.25 or 0.5 μg mL^{-1}), VitC (0.5 or 1 mM), and CCB (0.01 or 1 μM) in a 2% FBS-containing medium. Additionally, to stimulate the ADN *in vitro* model, podocytes were co-treated with AD (10 ng mL^{-1}) and GQDs (0.25 or 0.5 μg mL^{-1}).

Lastly, NIH3T3 fibroblasts (ATCC, CRL-1658, USA) were cultured in DMEM high glucose media (Biowest) supplemented with 10% FBS and 1% streptomycin. NIH3T3 fibroblasts were exposed to recombinant mouse TGF-β (2 ng mL^{-1}; R&D systems, **Table S4**) and GQDs (0.25 or 0.5 μg mL^{-1}) for 48 h to evaluate the anti-fibrotic role of GQDs in fibroblast-myofibroblast transition.

2.9 Assessment of Renal Function

Body weight, blood, and urine samples were obtained at baseline and at 2, 4, 6, and 8 weeks after 5/6 subtotal nephrectomy or 7 days after AD injection. After 24 h urine collection, urine protein and creatinine (Cr) concentrations were measured, and the urine protein/Cr ratio (mg mg^{-1}) was calculated. Blood urea nitrogen (BUN, mg dL^{-1}) and serum Cr (mg dL^{-1}) levels were determined by measuring the rate of the modified Jaffe reaction, and lactate dehydrogenase (LDH, U L^{-1}) levels were assessed in plasma 7 days post-AD injection to evaluate kidney infection and injury. All measurements were performed using an autoanalyzer (Hitachi Chemical Industries, Japan). Cr clearance was obtained using the following equation: clearance (mL min^{-1}) = [concentration of urine Cr (mg dL^{-1}) × urine volume in 24 h (mL)] / [concentration of plasma Cr (mg dL^{-1}) × 1440 min]. Blood pressure in the 5/6Nx model was measured using

the tail-cuff method (Kent Scientific Corporation, Torrington, CT, USA).

2.10 Histologic Analysis

Kidney tissues were fixed in 10% formalin and embedded overnight in paraffin. Four-micrometer-thick sections of the kidneys were deparaffinized and rehydrated with xylene and ethanol. Kidney sections were microwaved with a sodium citrate buffer for antigen retrieval. Endogenous peroxidase activity was blocked using a 3% hydrogen peroxide solution in methyl alcohol and incubated with primary antibodies (**Table S5**). Universal-specific antibodies (Agilent Technologies, Santa Clara, CA, USA, **Table S6**) were used to amplify the primary antibodies according to the manufacturer's instructions. Mayer's hematoxylin (Sigma-Aldrich) was used to counterstain cell nuclei. Kidney fibrosis and glomerular damage were assessed using Masson's trichrome and Periodic acid–Schiff staining. Sirius Red staining (Abcam, Cambridge, UK, **Table S6**) was used to assess collagen accumulation during kidney fibrosis. Stained slides were imaged using a Leica inverted microscope (Leica Camera, Wetzlar, Germany). After 5–8 fields (magnification 100× and 200×) were randomly selected, the LAS-4000 program (Leica Camera) was used to quantify the positive areas (%). All analyses were reviewed and confirmed by a renal pathologist who was blinded to the experimental groups. Glomerular sections (100 glomeruli at a magnification of 400×) were assessed by semi-quantitative analysis and scored according to the glomerular sclerosis index [2]: 0, normal glomeruli; 1, mesangial thickening of < 25% of the tuft; 2, moderate glomerular sclerosis with mesangial proliferation and thickening up to 50%; 3, severe glomerular sclerosis with obliteration of capillaries and diffuse sclerosis up to 75%; and 4, complete capillary obliteration and thrombosis with global sclerosis up to 100%.

2.11 Western Blotting

Total kidney proteins were isolated using RIPA buffer (150 mM NaCl; 100 mM Na3VO4; 50 mM Tris; HCL, pH 7.3; 0.1 mM EDTA 1% (vol/vol) sodium deoxycholate; 1% (vol/vol) Triton X-100; and 0.2% NaF; Biosesang, Gyeonggi-do, South Korea) with a protease inhibitor (GenDEPOT, Altair, TX, USA). Kidney tissues were crushed using steel beads in RIPA buffer using a TissueLyser instrument (Qiagen, Hilden, Germany) set at 30 strokes/s for 5 min. Unknown protein concentrations were determined using the bicinchoninic acid (BCA) assay.

The lysates were electrophoresed using glycine-sodium dodecyl sulfate buffer and transferred to polyvinylidene difluoride membranes (Amersham plc, Amersham, UK) on ice. The membranes were blocked for 1 h using a blocking solution (5% skimmed milk; Biosesang). Subsequently, the membranes were incubated with appropriate primary antibodies overnight at 4℃ with shaking (**Table S5**). Horseradish peroxidase-conjugated anti-mouse and anti-rabbit antibodies (Cell Signaling Technology, Danvers, MA, USA, **Table S7**) were mixed with 5% skimmed milk (Biosesang) in TBST and incubated for 1 h at 25℃. A mixture of ECL or SuperSignal West Femto Maximum Sensitivity Substrate (solutions A and B; Thermo Scientific, Waltham, MA, USA) was prepared and covered the top and bottom of the membrane. The membrane was visualized using ImageQuantTM LAS 4000 mini (Amersham plc) with optimal exposure time. Densitometry was performed using the gel analysis procedure in ImageJ (National Institutes of Health, Bethesda, MD, USA).

2.12 Quantitative Real-Time Reverse Transcription PCR (RT-qPCR)

TRIzol RNA isolation reagent (Invitrogen, Waltham, MA, USA) was used to isolate total RNA from the primary cultured podocytes. Complementary DNA synthesis was performed using AMV Reverse Transcriptase (Promega, Madison, WI, USA, **Table S8**) and a C1000 Touch Thermal Cycler (Bio-Rad, Hercules, CA, USA). SYBR-green dye-based RT-qPCR was performed using a 7500 Real-time PCR system (Applied Biosystems, Foster City, CA, USA). Thermocycler conditions were set up and operated according to a previously described protocol [3]. The PCR primers used are listed in **Table S9**. mRNA expression was calculated using the comparative Ct method ($2^{-\Delta\Delta Ct}$) after normalization to glyceraldehyde 3-phosphate dehydrogenase (*GAPDH*) or β-actin.

2.13 Confocal Microscopic Examination

4'-6-Diamidino-2-phenylindole (DAPI) (Thermo Fisher Scientific) was exposed by a violet (405 nm) laser line to stain nuclei, and podocyte-specific primary antibodies (WT-1, Zo-1, KLF15, and phalloidin) were used (**Table S5 and S10**). Alexa Fluor® 488-, 555-, and 647-conjugated antibodies (Invitrogen, **Table S11**) were excited using an argon 488 laser with 5% excitation power and 30% output. Consequently, 420–480-nm emission spectra were obtained for DAPI. Furthermore, 493-518 nm, 553–568 nm, and 650-671 nm emission spectra were

formed for green, red, and deep red or magenta fluorescence using an Acousto-Optical beam splitter. A photomultiplier detector platform was used to amplify the signal for an 800-gain setting, without interfering with the background frame for green, red, or deep red fluorescence. A hybrid sensor (HyD, Hamamatsu, Japan) was used to detect the electromagnetic spectrum of the DAPI signal after obtaining 100 detectors. The scanner specifications for confocal microscopy were 400 Hz using a 4X line average with 1 Airy unit (66.57 μm). The image pixel size was 1024 × 1024 XY in the sequential scanning mode of the LAS-X program (Leica).

Human podocytes were cultured on coverslips for 2 days and stained with MitoTracker (50 nM, Invitrogen, **Table S12**) following a standard protocol. After washing with PBS, the cells were fixed with 4% paraformaldehyde for 15 min, followed by additional washing and treatment with Triton X-100 and 10% normal goat serum. Confocal microscopy was then performed using a Leica DMI 6000 inverted microscope equipped with a TCS SP8 STED CW system and a 20×/0.7 NA objective lens.

2.14 Flow Cytometry Analysis

FACs experiments were conducted to identify changes in the phenotype of $CD3^+$ kidney infiltrating T lymphocytes and $CD11b^+$ macrophages according to the GQDs treatment in the ADR mouse model. Kidney tissues were harvested 1 week after AD administration to analyze T-cells and myeloid cells. Tissue homogenates were prepared using a Stomacher 80 Biomaster Laboratory Paddle Blender (Seward Ltd., Worthing, Sussex, UK). Single cells were isolated by passing the homogenate through a 40 μm cell strainer (BD Pharmingen, Franklin Lakes, NJ, USA). The cells were resuspended in 40% Percoll (Amersham Pharmacia Biotech, Piscataway, NJ, USA) and overlaid onto 80% Percoll. Subsequently, the Percoll gradient was separated by centrifugation at 1000 × g at 25°C for 30 min and incubated with fluorescence-conjugated mouse antibodies: CD3e (BD Pharmingen), CD4 (Invitrogen), CD8e (BD Pharmingen), CD25 (BD Horizon), and CD44 (eBioscience, San Diego, CA, USA) for the T-cell population, and CD11b (Invitrogen), CD206 (Invitrogen), and GR-1 (eBioscience) for kidney macrophages (**Table S13**). T-cells were permeabilized with the BD Cytofix/Cytoperm solution for intracellular staining, according to the manufacturer's instructions (BD Pharmingen), then stained with antibodies and fixed with 1% PFA. Fluorescence signals were detected using BD Canto (FACSDiva version 80; BD Biosciences), and FlowJo (version 10.0.7; FlowJo LLC,

Ashland, OR, USA) was used for FACs analysis.

2.15 Annexin V/PI Staining

The percentage of apoptotic and necrotic cells was measured using an Annexin V/PI fluorescein isothiocyanate (FITC) apoptosis kit (BD Biosciences, **Table S14**). Podocytes were resuspended in 100 μL binding buffer. Next, 5 μL FITC-conjugated Annexin V (10 mg mL^{-1}) and 10 μL PI (50 mg mL^{-1}) were added and incubated for 15 min at 25°C in the dark. Data was acquired and analyzed using BD FACSDiva^{TM} (version 8.0; BD Biosciences).

2.16 Intracellular Calcium Measurements

Calcium fluctuations were monitored using the Fura-2AM calcium influx assay kit (Abcam, **Table S15**), according to the manufacturer's protocol. Cells (2×10^4) were seeded in a 96-well black/clear bottom plate (ThermoFisher Scientific) and treated with ionomycin (5 mM; Sigma-Aldrich, **Table S15**) and GQDs (0.5 μg mL^{-1}) for 1 h in DMEM/F12 (2% FBS, Gibco). Before calcium measurement, 100 μL of a 1× Pluronic mixture with Fura-2AM was added and incubated for 1 h at 37°C, followed by 20 min at room temperature. The microplate was read at Ex/Em 340 nm/510 nm and Ex/Em 380 nm/510 nm using an Operetta CLS (PerkinElmer, Waltham, MA, USA).

2.17 Mitochondrial Membrane Potential

Mitochondrial voltage potential and ATP yield are highly correlated with oxidative phosphorylation, especially in the electron transport chain of the inner mitochondrial membrane. To observe the plasticity of mitochondrial membrane potential under oxidative stress conditions, human primary cultured podocytes and hTECs were stained with 100 nM MitoTracker Green (Invitrogen, **Table S12**), 100 nM tetramethylrhodamine, ethyl ester, and perchlorate (TMRM, Invitrogen, **Table S12**) in an incubator for 15 min. Next, the fluorescence was excited at 488 nm (MitoTracker Green) and 548 nm (TMRM). To detect mitochondrial reactive oxygen species (ROS) production, incubator-stained renal cells were incubated for 10 min with 5 μM MitoSOX Red (Invitrogen, **Table S12**). The plate was then washed three times

with warm buffer. For counterstaining, DAPI (Invitrogen, **Table S12**) was stained, and images were acquired using an Operetta CLS (PerkinElmer).

2.18 Mitochondrial Respiratory Assay

Mitochondrial respiration parameters, including the basal, ATP production-linked, maximal, and proton leak-linked oxygen consumption rate (OCR), were measured using the Seahorse XF ATP Real-Time rate assay (Agilent Technologies, Santa Clara, CA, USA, **Table S16**). Briefly, podocytes treated with H_2O_2 (0.5 mM) and GQDs (0.25 or 0.5 μg mL^{-1}) were seeded onto a Seahorse plate designed to run on a Seahorse Machine and placed in the hood for 2 h. On the day of the experiment, cells were washed with DMEM/F12 (2% FBS, Gibco) and resuspended in Seahorse XF medium at a concentration of 100,000 cells/180 μL. The plate was then incubated for 1 h and centrifuged at 1500 rpm for 5 min. Data analysis was performed using "wave" software from Seahorse (Agilent Technologies).

2.19 JC-1 Assay

To analyze mitochondrial membrane potential under oxidative stress conditions induced by H_2O_2, JC-1 fluorescence detection for flow cytometry was performed using the MitoProbe JC-1 Assay kit (Invitrogen, **Table S16**) with a few modifications. Two positive controls were used: 1 μL of 50 mM CCCP (supplied with the kit) and 1 mM H_2O_2. Both were incubated at 37°C for 5 min. Next, JC-1 (10 μL of 200 μM) was added to each sample and incubated at 37°C with 5% CO_2 for 30 min. After JC-1 staining, JC-1 was detected at 488 nm and 633 nm using a BD FACS Canto (FACSDiva version 80).

2.20 Permeability Assays in Human Podocytes

Using an albumin-rhodamine transit assay, the rate of transport of human podocytes across the Transwell system was measured in 24-well plates (NINC A/S, Roskilde, Denmark) with 0.4 μm pore membranes. DMEM/F12 (Biowest) containing albumin-rhodamine (Abcam, **Table S17**) media was added to a monolayer of podocytes. Albumin-rhodamine fluorescence intensity was quantified once the albumin-rhodamine in the lower well had increased after 24 h, after

being treated with 2 ng/mL of AD and 0.25 or 0.5 μg mL^{-1} of GQDs or under fibrotic conditions induced by rotational force. A standard curve was used to calculate the unknown albumin-rhodamine concentration at Ex/Em 550 nm/570 nm using a spectrophotometer.

2.21 Induction of Fibrosis with a Mechanical Stress Device

Our previous report demonstrated that hypertensive-mimicking devices successfully induce endothelial dysfunction in response to podocyte injury in patients with hypertensive nephrosclerosis [4]. Human primary podocytes with or without GQDs were cultured in 6-well culture plates (1×10^5 cells/well) and inserted into dishes attached to a mechanical stress device (rotational force device). The mechanical stress device was placed in an incubator containing 5% CO_2 at 37°C. The operating time of the rotational force device (4 mmHg) was set to 48 h, and the group treated with GQDs (0.5 μg mL^{-1}) 3 h before device placement was defined as the pre-treatment group. Conversely, the group treated with GQDs (0.5 μg mL^{-1}) 6 h after the device placement was defined as the post-treatment group. Subsequently, molecular approaches were used to assess oxidative stress in podocytes, and permeability assays were used to investigate apoptosis.

2.22 ROS Assay

Intracellular ROS levels were evaluated by monitoring the increase in DCF fluorescence intensity. Human podocytes were seeded in multi-well plates and treated with H_2O_2 (1mM) and GQDs (0.25 μg mL^{-1} and 0.5 μg mL^{-1}) in a dose-dependent manner. After treatment, the cells were incubated with 10-μM DCFH-DA (Cell Biolabs, San Diego, CA, USA, **Table S17)** for 1 h at 37°C. Fluorescence intensity in the cells was measured using a fluorescence spectrometer (Tecan, Research Triangle Park, NC, USA).

2.23 β-galactosidase Staining and Wound Healing Scratch Assay

The Senescence β-Galactosidase Staining Kit (Cell Signaling, **Table S18**) was used to detect cellular senescence and evaluate the therapeutic effect of GQDs under ROS-induced stress, with imaging performed via a Leica inverted microscope (Leica camera). The Live/Dead assay

(Invitrogen, **Table S18**) was conducted to quantify the number of dead cells following exposure to H_2O_2. The assay employs calcein-AM to stain live cells (Ex/Em = 494/517 nm) and ethidium homodimer-1 (Ex/Em = 528/617 nm) to label dead cells. The Live/Dead assay and the wound healing capability evaluation were visualized and analyzed using the Operetta CLS high-content analysis system (PerkinElmer). Cell migration from the wound edge into the unoccupied area was examined for wound healing analysis. The z-stack function ensured optimal focus, and each well was evaluated, producing multi-field stitched images accompanied by bar graphs and integrated realistic plate visuals. Cell counts and migration tracking were performed using digital phase contrast (DPC) for detailed analysis.

2.24 Singleplex Protein Analysis

The supernatants collected after exposure to H_2O_2 were analyzed using a singleplex cytokine bead array system (Bio-Plex cytokine assay, Bio-Rad) according to the manufacturer's instructions. The reaction mixture was then measured using the Bio-Plex protein array reader, and the data were analyzed with the Bio-Plex Manager software program.

2.25 Electron Microscopy

Kidney sections were fixed with 2.5% glutaraldehyde solution (Sigma-Aldrich), post-fixed in osmium tetroxide, and embedded (LADD Research Industries Inc., Williston, VT, USA). TEM sections were stained with lead citrate and uranyl acetate and were examined using a JEM-1400 electron microscope (JEOL Ltd., Akishima, Tokyo, Japan). Here, five areas from 1–2 glomeruli in each animal were photographed in random spots, with a final magnification of 12,000× and 30,000×. Random photographs were obtained at a final magnification of 80,000× to study the podocyte foot process. Mitochondria were photographed at a final magnification of 50,000× and 100,000× for the glomerulus and tubule, respectively. Mitochondrial lengths, cristae area, and volume density were quantitatively assessed using ImageJ software, following the protocol [5]. Images were captured at magnifications of 12,000× for tubular mitochondrial cristae and 50,000× for glomeruli mitochondrial cristae.

2.26 Hydroxyl Radical Antioxidant Capacity (HORAC) Assay

The antioxidant capacity of GQDs was evaluated in comparison to established antioxidants, specifically ascorbic acid (Sigma-Aldrich, **Table S2**) and amlodipine (Sigma-Aldrich, **Table S2**), utilizing the OxiSelect HORAC Activity Assay (Cell Biolabs, **Table S19**) according to the provided protocol (**Table S19**). Briefly, gallic acid standards and podocyte lysates were diluted and mixed with 1X fluorescein solution in a 96-well microtiter plate (SPL Life Sciences, 30096 Gyonggi-do, South Korea). Subsequently, a hydroxyl radical initiator and a Fenton reagent were added to initiate the reaction. The fluorescence intensity was monitored using BioTek Synergy Neo2 (Agilent Technologies) at an excitation wavelength of 480 nm and an emission wavelength of 530 nm in increments of 1 minute over a total duration of 60 minutes.

2.26 3-(4,5-dimethythiazol-2-yl)-5-(3-carboxymethoxyphenyl)-2-(4-sulfophentyl)-2H-tetrazolium (MTS) assay

Human primary cultured podocytes and HK-2 cells (a human proximal tubular cell line) were seeded on a 96-well clear-bottom plate (SPL Life Sciences) and incubated overnight. GQDs were then added in a dose-dependent manner (0, 0.125, 0.25, 0.5, 1, and 2 μg mL^{-1}) to assess cell viability in response to GQDs cytotoxicity. Ez-Cytox (DOGenBio, **Table S17**) was added to the 96-well cell culture plate and incubated for 1 hour at 37°C. The absorbance was measured at 450 nm, and the results were visualized and analyzed using GraphPad Prism 10.2.3 (GraphPad Software, Inc., San Diego, CA, USA).

2.28 mRNA-sequencing Analysis

Total kidney RNA was extracted using the TRIzol reagent (Invitrogen). EBIOGEN, Inc. (Seoul, South Korea) conducted RNA sequencing and data analysis. An Agilent 2100 Bioanalyzer (Agilent Technologies) was used to assess RNA quality. A library was constructed using the NEBNext Ultra II Directional RNA-seq kit (New England BioLabs, Ipswich, MA, USA) according to the manufacturer's protocol. mRNA was isolated using a poly (A) RNA selection kit (LEXOGEN, Vienna Biocenter, Austria). High-throughput sequencing was conducted as paired-end 100 sequencing using HiSeq X10 (Illumina, San Diego, CA, USA). DEGs and gene ontologies were determined and analyzed using the ExDEGA package provided by EBIOGEN Inc. (Seoul, South Korea). Kyoto Encyclopedia of Genes and Genomes (KEGG) pathway and

Gene Ontology (GO) enrichment analyses (**Tables S20–S22**) were conducted using the R packages enrichGO and enrichKEGG. Network analysis for DEGs was performed using Cytoscape (version 3.10.0). The MeV program was used to generate and analyze a heatmap of differentially expressed genes (DEGs) based on the results of GO enrichment and KEGG pathway analyses.

2.29 Illustrations

All schematic illustrations or diagrams *in vivo* and *in vitro* model were created with BioRender.com.

2.30 Statistical Analyses

All data are expressed as the mean ± standard error of the mean. Two groups were compared using a two-tailed unpaired Student's t-test, conducted with GraphPad Prism 10.2.3 (GraphPad, USA). The R packages of enrichGO and enrichKEGG were used to calculate p-values for mRNA-seq analysis. A one-sample t-test was performed using R, and the false discovery rate (FDR) value was calculated using the following equation: [p-value (one-sample t-test)] × [(total number of ranks) / (number of rank)]. The correlation coefficient (r) indicates the relationship between specified targets and TRPC5. For all statistical tests, p-values of < 0.05 were considered significant.

References

[1] H. A. Jo, J. H. Seo, S. Lee, M. Y. Yu, E. Bae, D. K. Kim, Y. S. Kim, D. J. Kim, S. H. Yang, *Sci Rep* **2023**, *13* (1), 2026, https://doi.org/10.1038/s41598-023-28850-3.

[2] S. H. Yang, S. J. Shin, J. E. Oh, J. Z. Jin, N. H. Chung, C. S. Lim, S. Kim, Y. S. Kim, *Nephrol Dial Transplant* **2008**, *23* (11), 3437, https://doi.org/10.1093/ndt/gfn268.

[3] S. S. Han, M. Y. Yu, K. D. Yoo, J. P. Lee, D. K. Kim, Y. S. Kim, S. H. Yang, *Int J Mol Med* **2018**, *42* (3), 1593, https://doi.org/10.3892/ijmm.2018.3726.

[4] M. Y. Yu, J. E. Kim, S. Lee, J. W. Choi, Y. C. Kim, S. S. Han, H. Lee, R. H. Cha, J. P. Lee, J. W. Lee, D. K. Kim, Y. S. Kim, S. H. Yang, *Exp Cell Res* **2020**, *386* (1), 111706,

https://doi.org/10.1016/j.yexcr.2019.111706.
[5] J. Lam, P. Katti, M. Biete, M. Mungai, S. AshShareef, K. Neikirk, E. Garza Lopez, Z. Vue, T. A. Christensen, H. K. Beasley, T. A. Rodman, S. A. Murray, J. L. Salisbury, B. Glancy, J. Shao, R. O. Pereira, E. D. Abel, A. Hinton, Jr., *Cells* **2021**, *10* (9), https://doi.org/10.3390/cells10092177.

Supplementary Materials for
Effects of Graphene Quantum Dots on Renal Fibrosis Through Alleviating Oxidative Stress and Restoring Mitochondrial Membrane Potential

Figure S1. Characterization of graphene quantum dots
Figure S2. Quantification of the results of flow cytometry
Figure S3. Western immunoblot data for total kidney in GQDs-treated ADN mice
Figure S4. 3D PCA analysis and scatter plot of mRNA-seq in ADN mouse model
Figure S5. Top 10 KEGG and GO enrichment analysis from ADN+GQDs mouse model
Figure S6. Clusters of mRNA-seq in the ADN+GQDs mouse model
Figure S7. Analysis of gene expression clustering and associated biological process in GQDs-treated ADN mice
Figure S8. Heatmap of GO enrichment for Apoptotic signaling pathway regulation
Figure S9. Western immunoblot data for total kidney in a 5/6Nx rat model
Figure S10. Western blotting result of TRPC6 in animal models
Figure S11. GQDs prevent senescence in podocytes
Figure S12. GQDs promote the wound-healing in podocytes
Figure S13. GQDs promote mitochondrial renewal in human podocytes
Figure S14. Relationship between the IHC results of certain targets in GQDs-treated ADN mice (A), and GQDS-treated 5/6Nx rat (B) with TRPC5
Figure S15. Pre-treatment with GQDs demonstrates greater therapeutic effectiveness in the 5/6Nx model, rescuing cell viability in a device-based hypertension condition
Figure S16. GQDs exhibit therapeutic effects comparable to standard agents, vitamin C and CCB
Figure S17. GQDs attenuate kidney fibrosis after targeting TRPC5 channel in podocytes
Figure S18. GQDs exhibit antioxidative effect in podocyte.

Table S1. Reagents for GQDs quality control experiments
Table S2. Agents with antifibrotic and antioxidant properties
Table S3. Antibodies used for identifying human primary cells
Table S4. Media and manufactured details
Table S5. Primary antibodies
Table S6. Staining kit and secondary antibodies for IHC experiments
Table S7. HRP-conjugated secondary antibodies for western blotting
Table S8. RT-PCR kit
Table S9. Primer sequences
Table S10. Chemicals for immunofluorescence staining
Table S11. Secondary antibodies for immunofluorescence staining
Table S12. Mitochondria tracers
Table S13. Antibodies for Flow Cytometry
Table S14. Annexin V/PI staining kit
Table S15. Intracellular calcium indicator kit
Table S16. Mitochondrial respiratory assay
Table S17. ROS assay
Table S18. Assay for senescence
Table S19. Assay for hydroxyl radical antioxidant capacity
Table S20 - 22. Analysis for gene expression clustering and associated biological process in GQDs-treated ADN mice
Table S23. Real-time measurements of OCR in podocytes

Figure S1

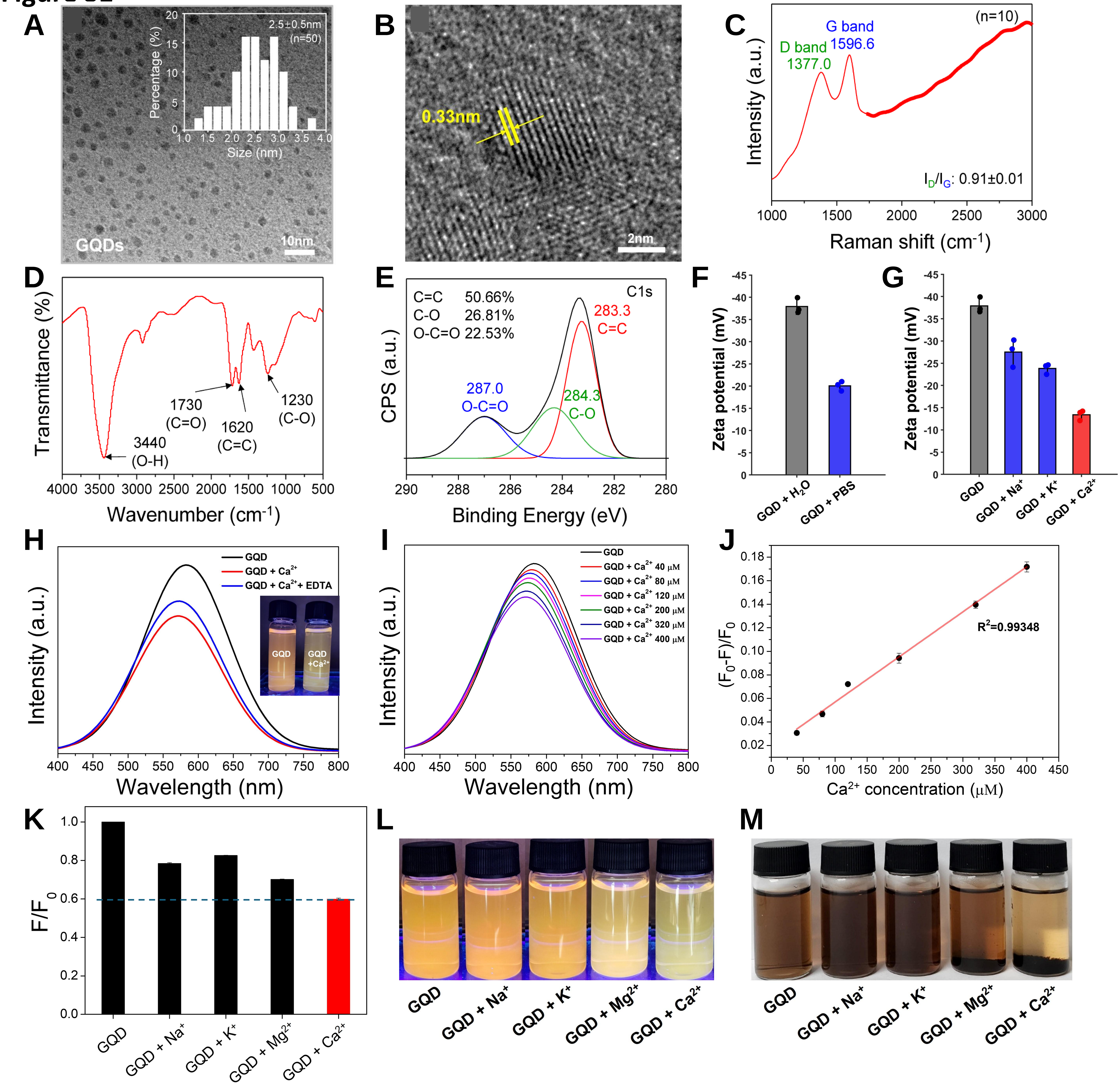

Figure S1. Characterization of graphene quantum dots (A) HR-TEM image of GQDs measuring 2–5 nanometers in diameter and the size distribution of GQDs (average diameter, 2.5 ± 0.5 nm, n = 50) Scale bar, 10 nm. (B) Cs-TEM image of GQDs with atomic resolution. The d-spacing of lattice in GQDs is approximately 0.33 nm. Scale bar, 2 nm. (C) Raman spectrum of GQDs showing D band (1377 cm^{-1}) and G band (1596 cm^{-1}). (I_D/I_G ratio = 0.91 ± 0.01, n = 10). (D) FT-IR spectrum of GQDs showing O-H bond, C=O bond, C=C bond, and C-O bond at 3440 cm^{-1}, 1730 cm^{-1}, 1620 cm^{-1}, and 1230 cm^{-1}, respectively. This indicates that GQDs are highly oxidized and have oxygen-rich functional groups such as hydroxyl and carboxyl groups. (E) XPS C1s spectrum of GQDs showing O-C=O bond, C-O bond, and C=C bond at 287.0 eV, 284.3 eV, and 283.3 eV, respectively. (F) Zeta potential of GQDs in DI water (pH = 7, -37.9±1.8 mV) and 10 mM PBS (pH = 7.4, -20.1±1.1 mV). GQDs shows negative surface charges in both solvent. (G) Zeta potential of GQDs in different ion solutions. Zeta potential of GQDs (-37.9±1.8 mV) is more increased with Ca^{2+} ions (-13.4±1.1 mV) than that with monovalent ions Na^{+} (-27.5±3.1 mV) and K^{+} (-23.8±1.2 mV), indicating their strong absorption toward Ca^{2+} ions. (H) Photoluminescence (PL) spectrum of GQDs with or without calcium ions (at Ex =365 nm). Decreasing PL intensity and blue-shift of emission at maximum intensity under Ca^{2+} condition and recovering PL by addition of competitive Ca^{2+} chelator EDTA. (I) The PL intensity changes of GQDs solution depends on different Ca^{2+} concentration (at Ex = 365 nm). (J) The PL quenching ratio of GQDs depends on Ca^{2+} concentration. (F_0: PL intensity of GQDs without Ca^{2+}, F: PL intensity of GQDs with Ca^{2+}, n = 3) This indicates that GQDs closely interact with Ca^{2+} which enable to transition of the photo-induced charges from GQDs (R^2 = 0.99348). (K) PL quenching of GQDs under different ions (at Ex = 365 nm, F_0: PL intensity of GQDs without ion, F: PL intensity of GQDs with ion, n = 3). Ca^{2+} ions lead to significant PL quenching of GQDs compared to other cations. (L) Photograph illustrating the light emission of the GQDs solution depending on the added ions under UV 365 nm. The color of GQDs changes from orange to yellow upon the addition of divalent cations. (M) Photograph illustrating the aggregation behavior of GQDs after 24 h of excessive ions addition (10 mM). The aggregation efficiency of GQDs is particularly high with Ca^{2+} ions.

TEM, transmission electron microscopy; HR-TEM, high-resolution TEM; Cs-TEM, corrected scanning TEM; GQDs, graphene quantum dots

Figure S2

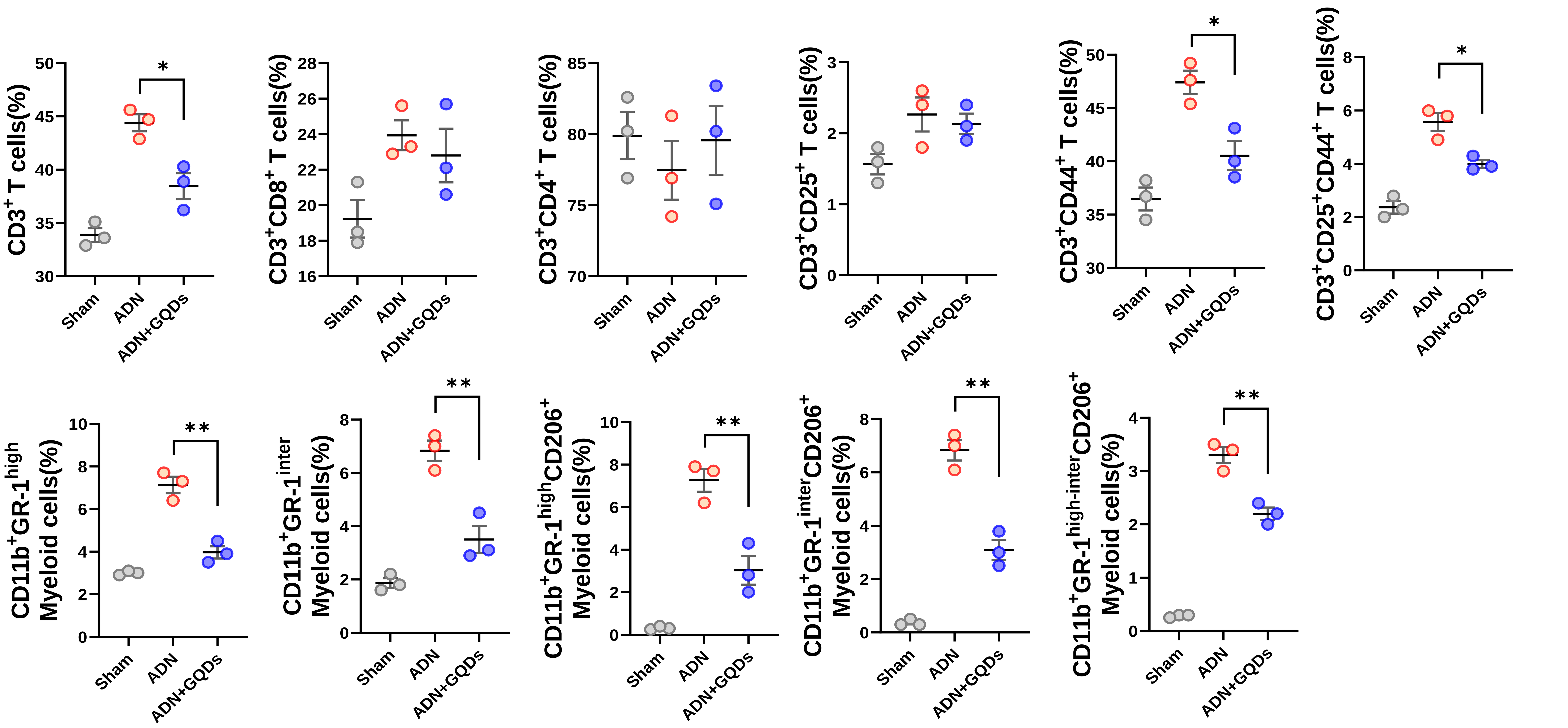

Figure S2. Quantification of the results of flow cytometry. Kidney-resident immune cell types in the ADN mouse model represented two populations [kidney T-cells, $CD3e^+CD8e^+CD4^+CD25^+CD44^+$; kidney myeloid cells, $CD11b^+GR\text{-}1^+CD206^+$] (n = 3 per group). ADN, Adriamycin-induced nephropathy; GQDs, graphene quantum dots

Figure S3

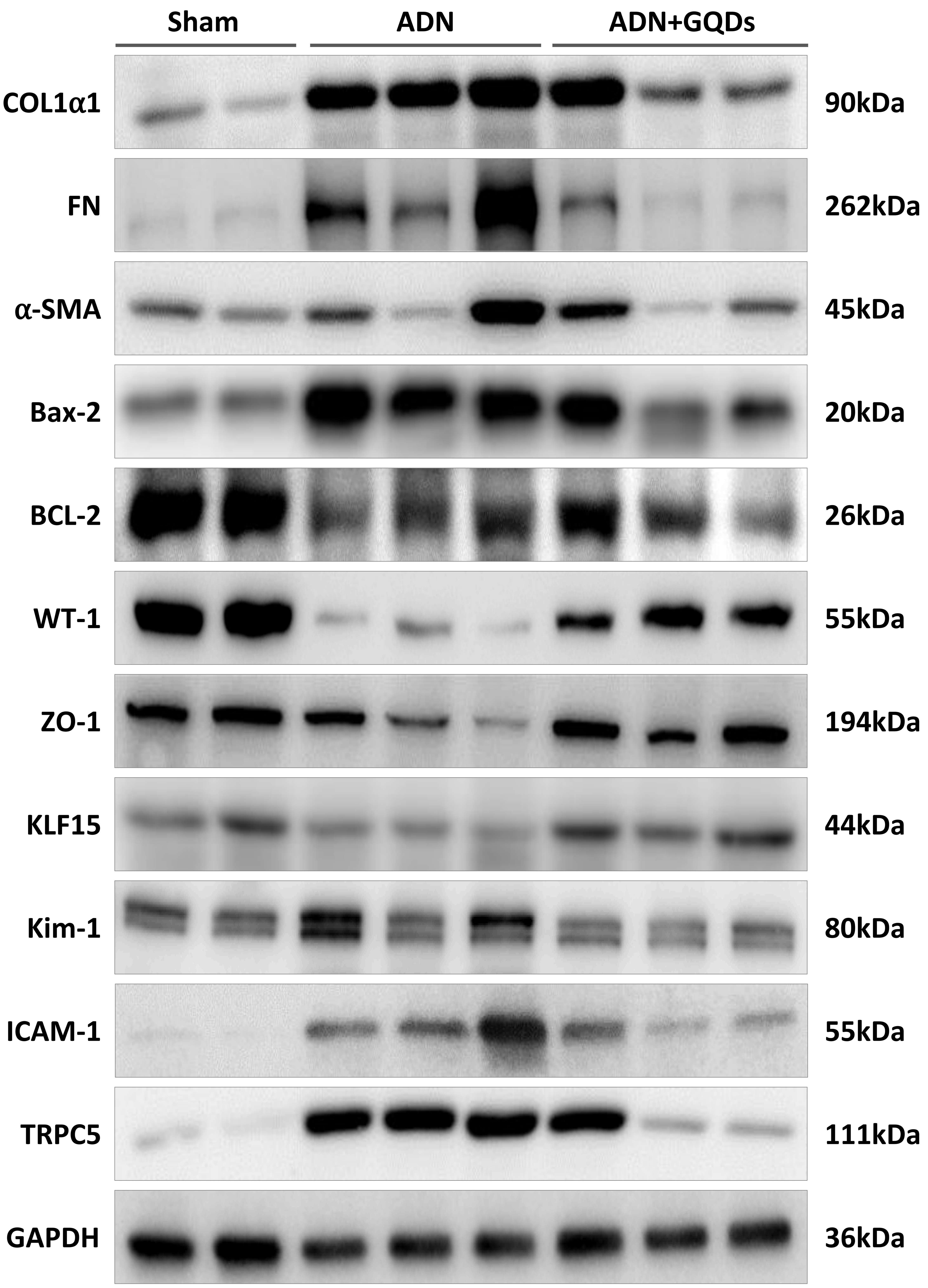

Figure S3. Western immunoblot data for total kidney in GQDs-treated ADN mice. Western blot analysis of proteins that were associated with kidney fibrosis, the apoptotic pathway, apical junction, and the podocyte cytoskeleton from kidney tissue in mice given a single intraperitoneal injection of AD (11.5 mg kg^{-1}) and GQDs (20 mg kg^{-1}) for the GQDs-treated group up to 7days. ADN, Adriamycin-induced nephropathy; GQDs, graphene quantum dots

Figure S4

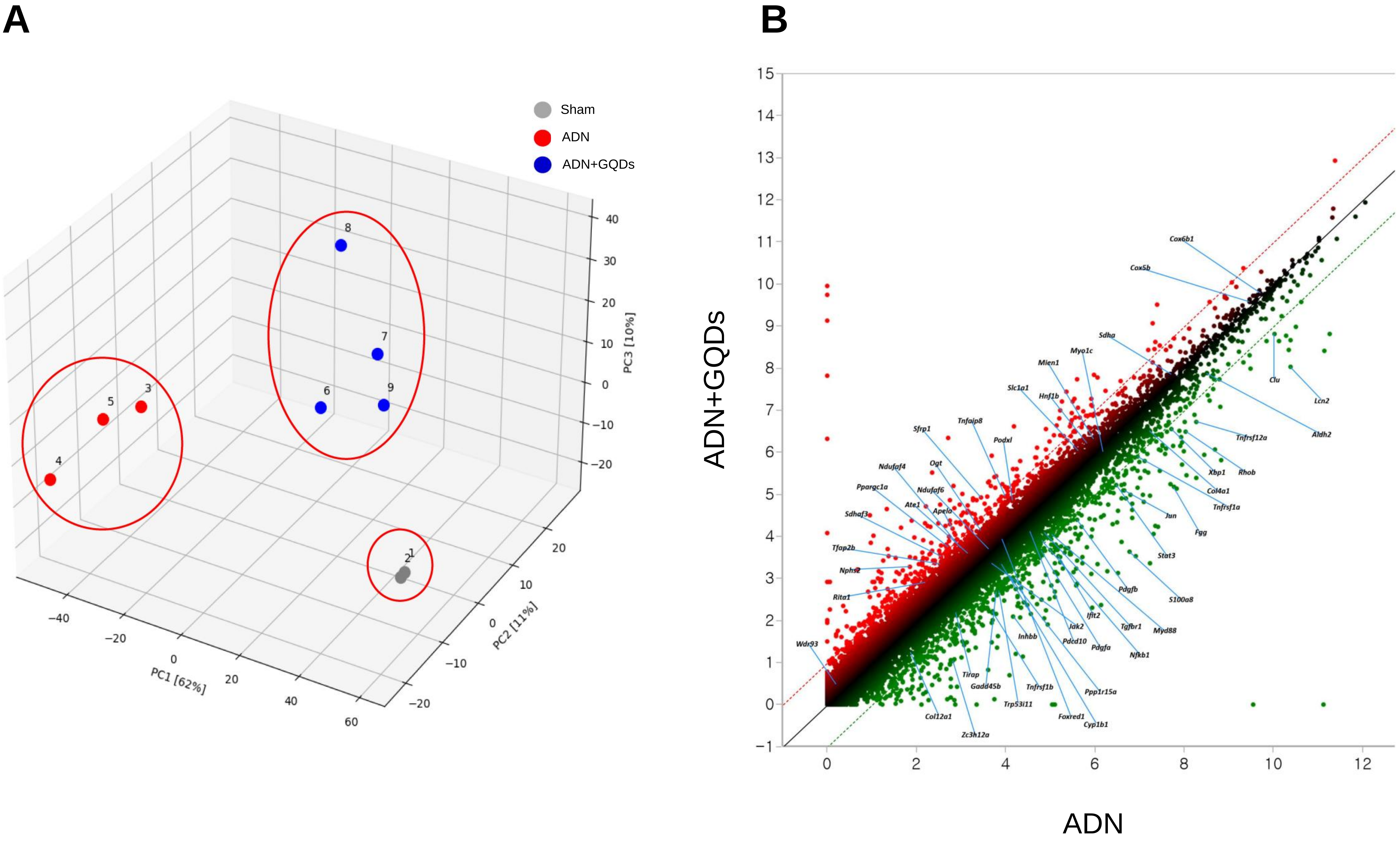

Figure S4. 3D PCA analysis and scatter plot of mRNA-seq in the ADN mouse model. (A) 3D score plot from the PCA of mRNA sequencing data reveals similarities among mice with respect to the first two principal components (B) The scatter plot illustrates DEGs in the ADN+GQDs model, with upregulated genes indicated in red and downregulated genes in green. ADN, Adriamycin-induced nephropathy; GQDs, graphene quantum dots

Figure S5

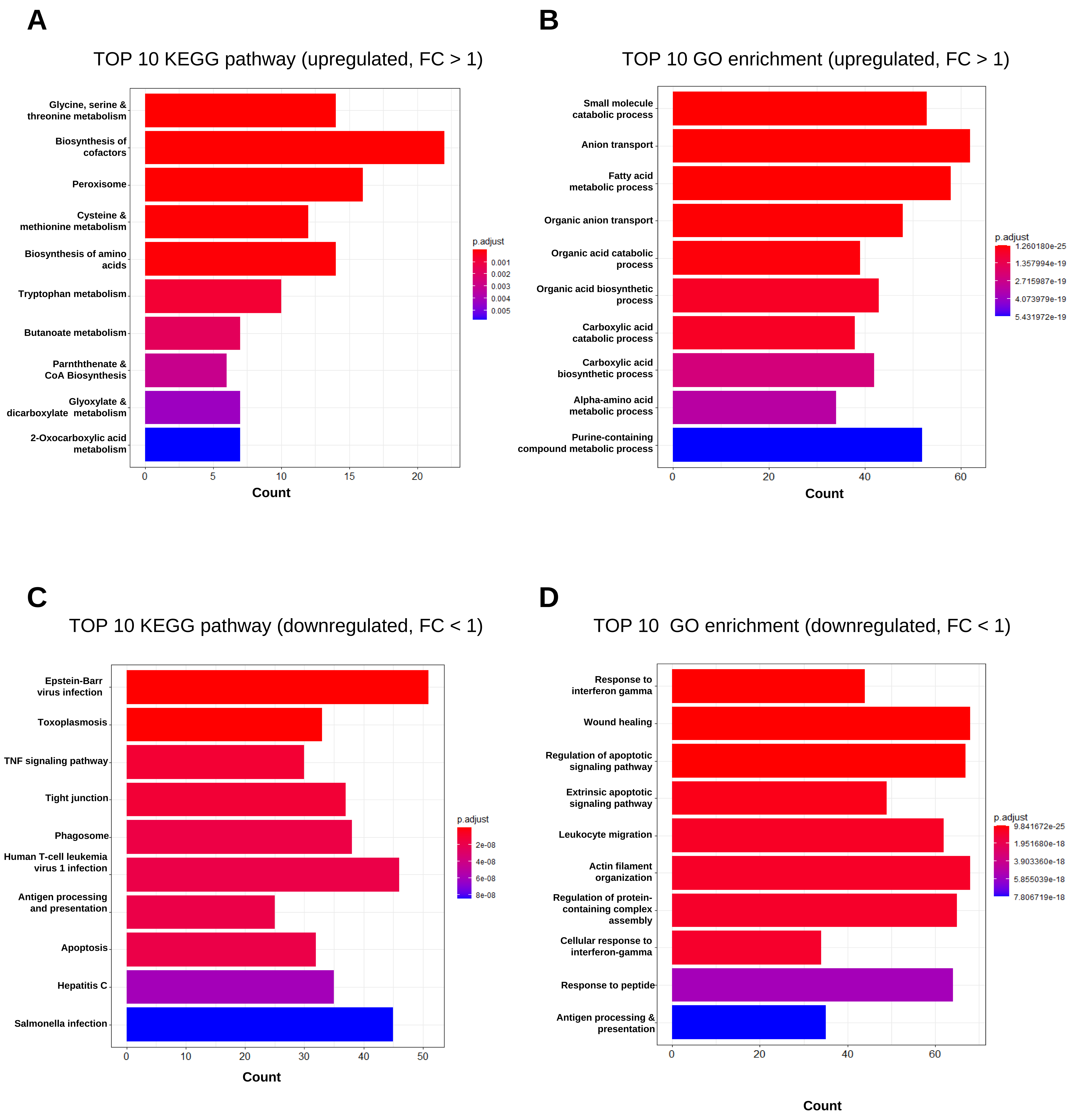

Figure S5. Top 10 KEGG and GO enrichment analysis from the ADN+GQDs mouse model. (A and B) The top 10 GO enrichment analyses of upregulated (A) and downregulated (B) DEGs were shown. (C and D) Top 10 KEGG pathways analysis DEGs were further analyzed. ADN, Adriamycin-induced nephropathy; GQDs, graphene quantum dots

Figure S6

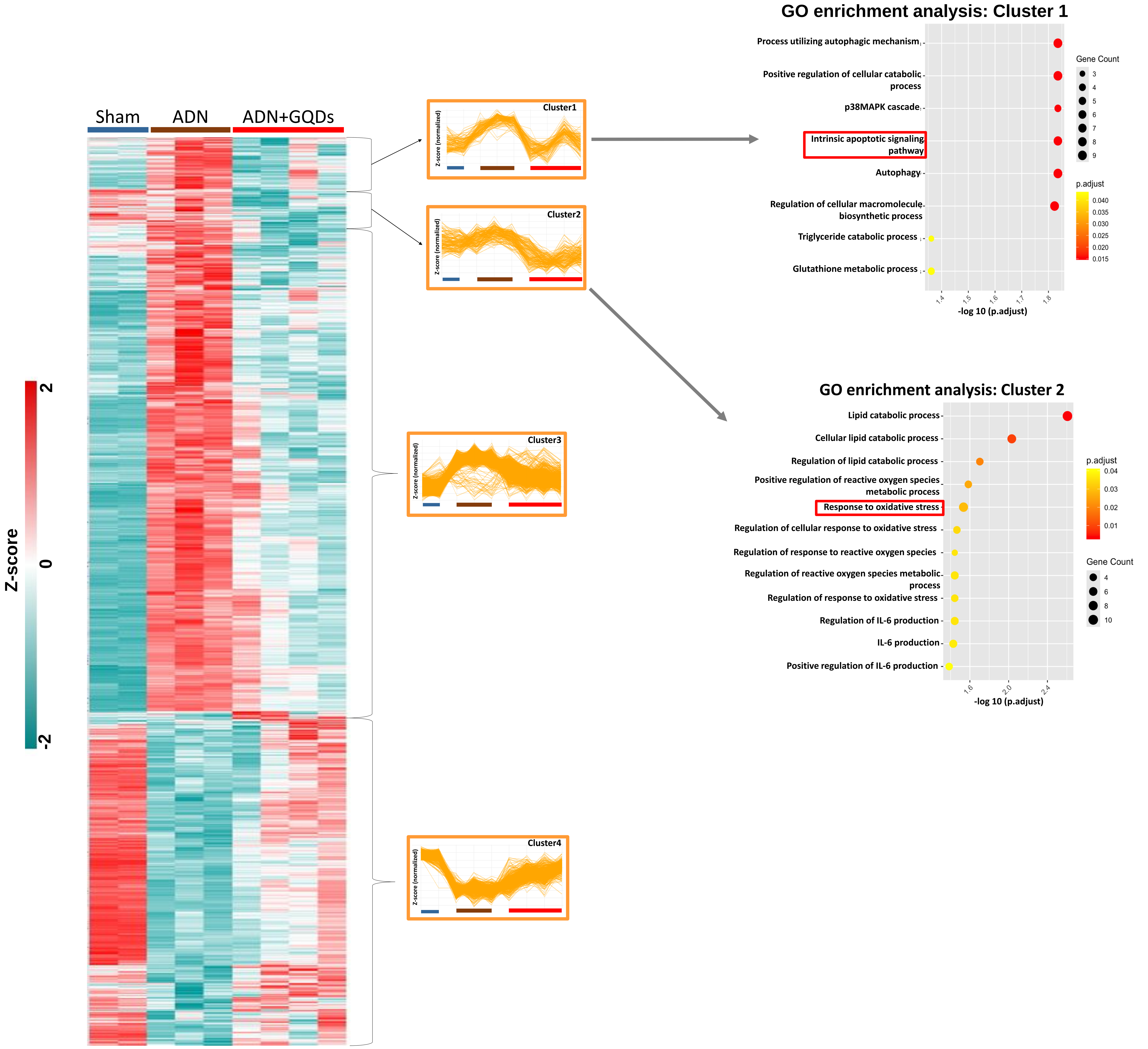

Figure S6. Clusters of mRNA-seq in the ADN+GQDs mouse model. The figure shows a comprehensive heatmap of mRNA-seq data in the ADN+GQDs model, with clusters organized based on differentially expressed genes (DEGs). Dot plots representing the GO enrichment for clusters 1 and 2 were subjected to further analysis to elucidate their biological significance. ADN, Adriamycin-induced nephropathy; GQDs, graphene quantum dots.

Figure S7

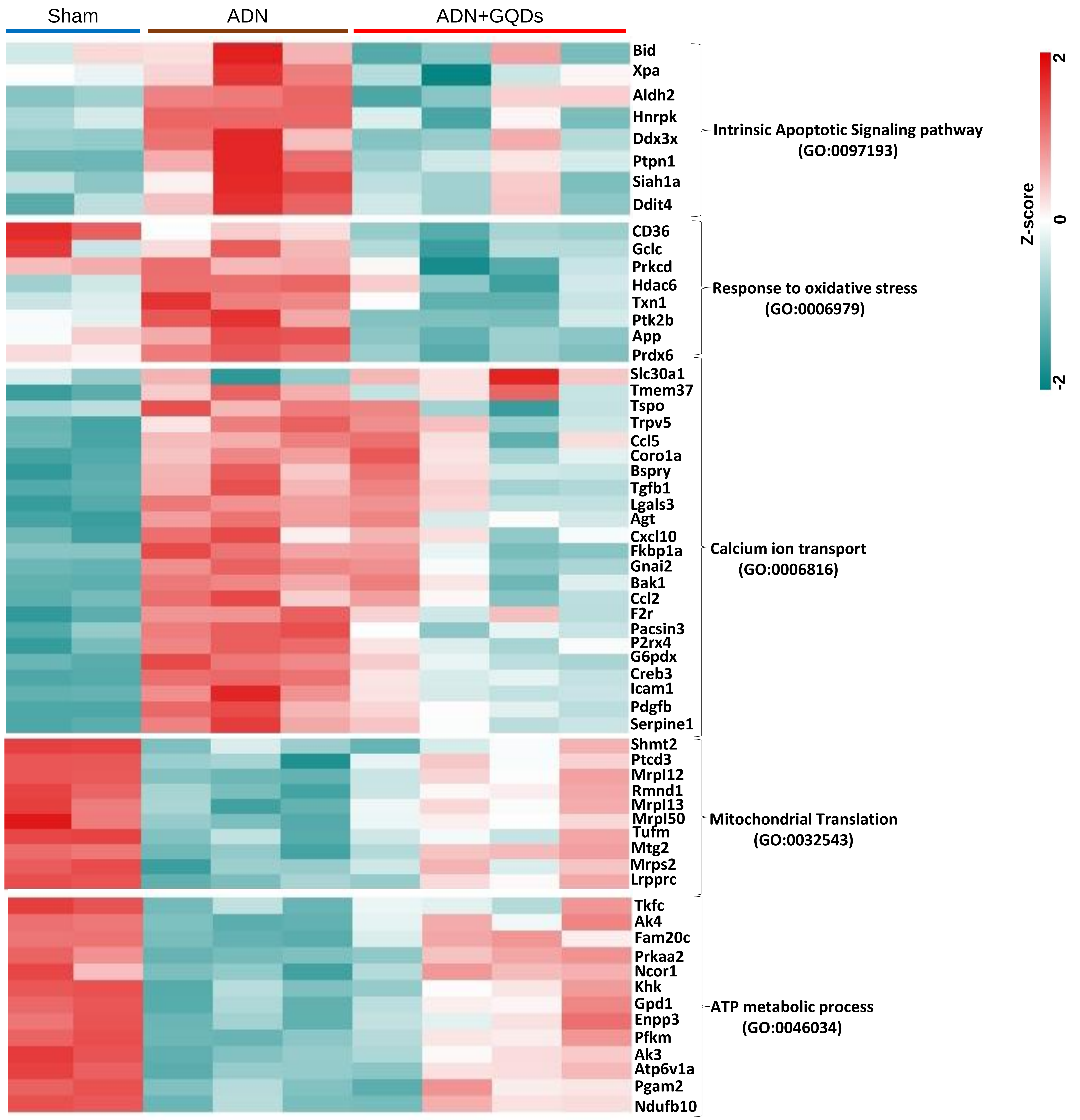

Figure S7. Analysis of gene expression clustering and associated biological process in GQDs-treated ADN mice. Heatmaps present detailing dealing gene clusters involved in the intrinsic apoptotic signaling pathway (GO:0097193), response to oxidative stress (GO:0006979), calcium ion transport (GO:0006816), mitochondrial translation (GO:0032543), and ATP metabolic process (GO:0046034). ADN, Adriamycin-induced nephropathy; GQDs, graphene quantum dots

Figure S8

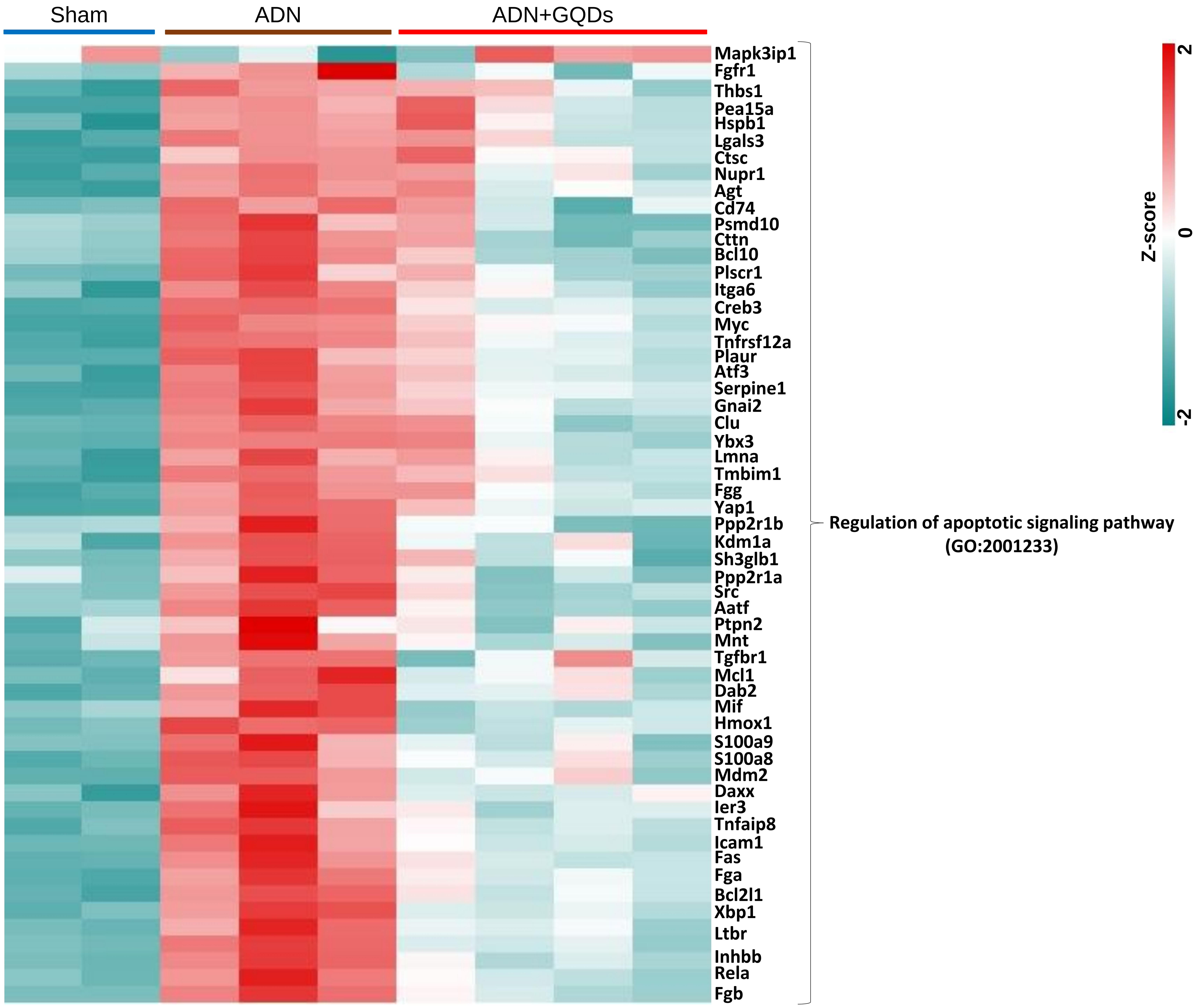

Figure S8. Heatmap of GO enrichment for apoptotic signaling pathway regulation. Heatmap represents gene clusters associated with the regulation of the apoptotic signaling pathway (GO:2001233) in GQDs-treated ADN mice

Figure S9

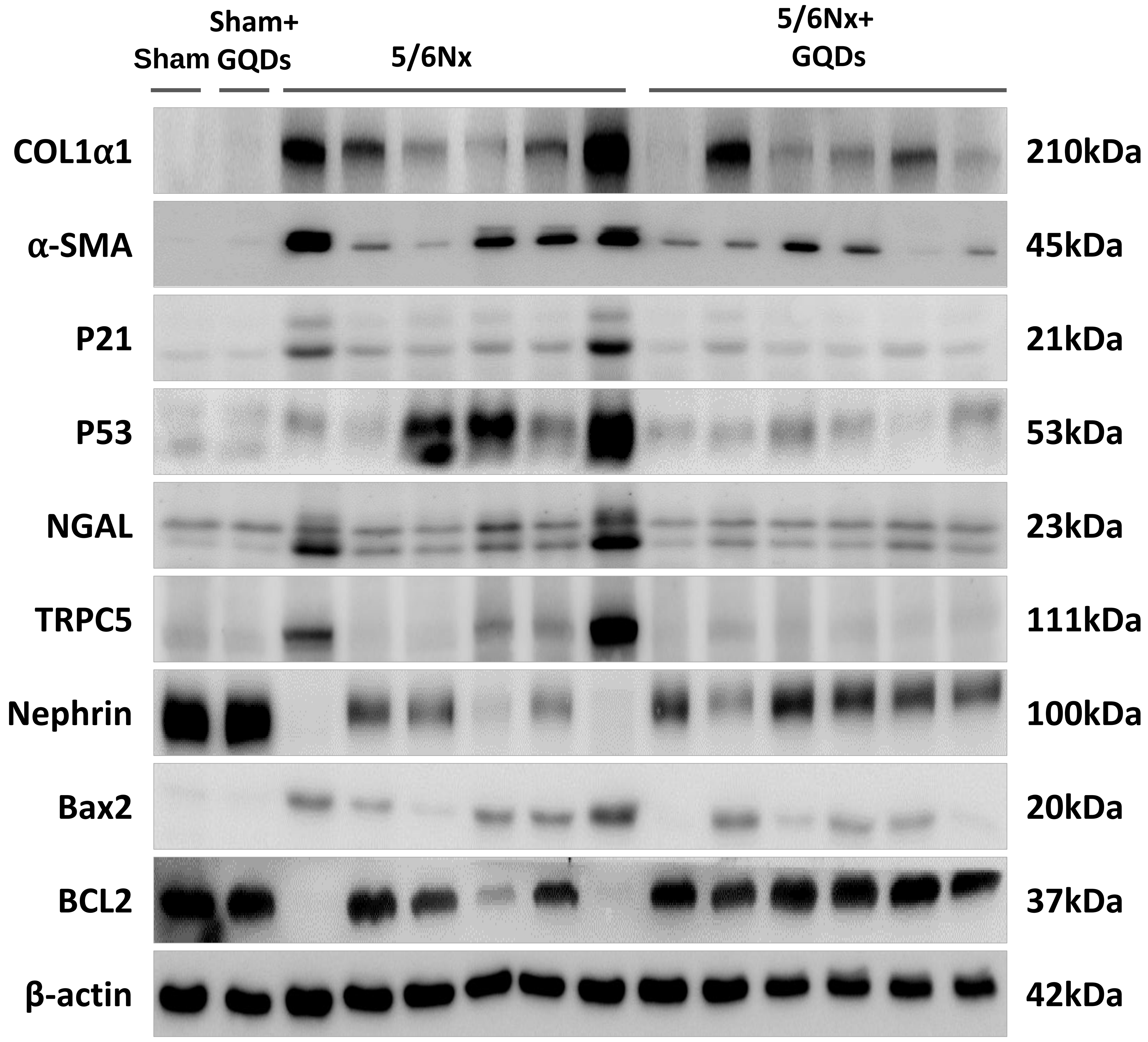

Figure S9. Western blot of total kidney in a 5/6Nx rat model. Kidney tissues from the indicated groups (sham, 5/6Nx, and 5/6Nx+GQDs) were western blotted and showed typical bands for renal fibrosis, cyclin-dependent kinases, cell viability, and podocyte biology. Animals were subjected to 5/6Nx and GQDs (4 mg kg^{-1}, I.P) administration for 8 weeks. GQDs, graphene quantum dots.

Figure S10

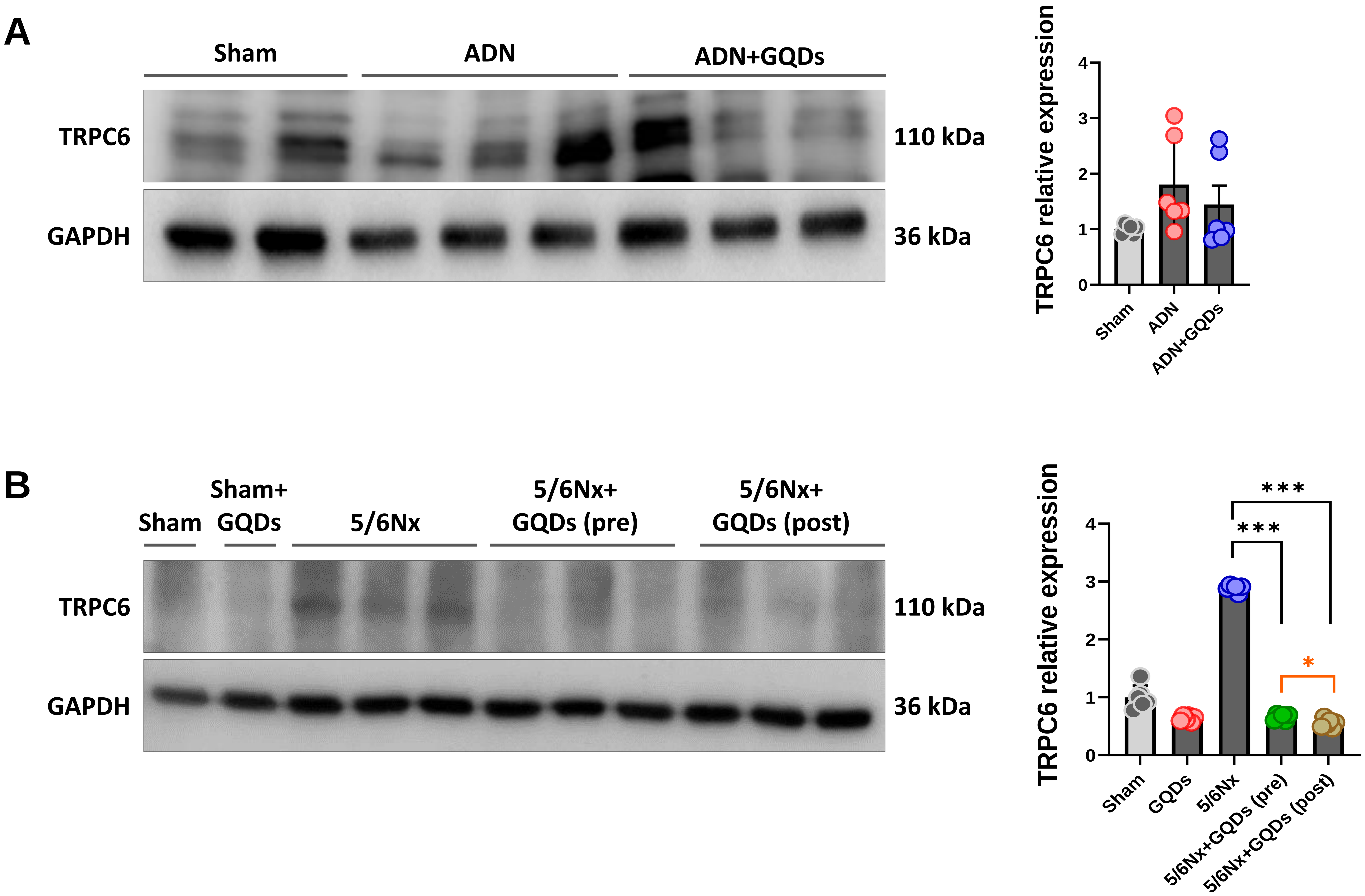

Figure S10. Western blot of TRPC6 in animal models. (A and B) The expression of TRPC6 was observed in both ADN+GQDs and 5/6Nx+GQDs animal models (n = 6 per group). ADN, Adriamycin-induced nephropathy; GQDs, graphene quantum dots

Figure S11

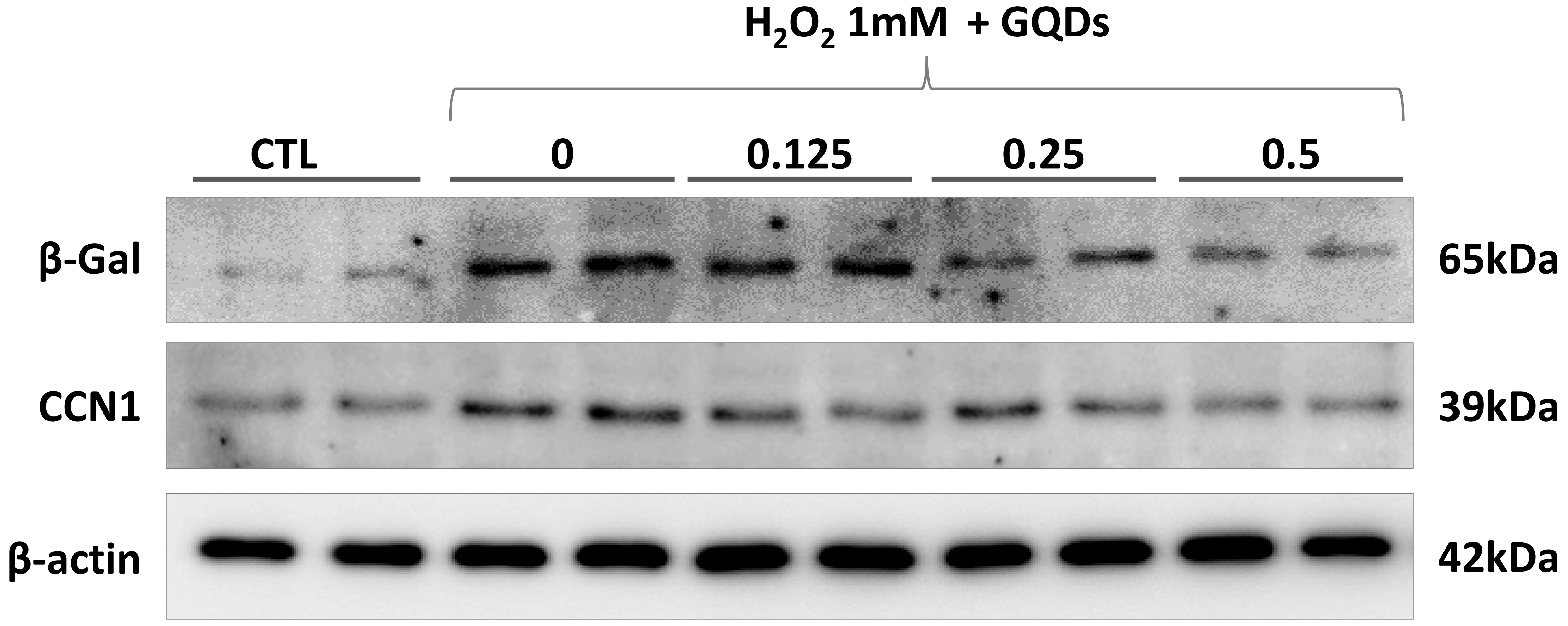

Figure S11. GQDs prevent senescence in podocytes. Representative western blotting images display the protein level of β-Gal and CCN1 following exposure to H_2O_2 (1 mM) and GQDs (0.25 and 0.5 μg mL^{-1}). GQDs, graphene quantum dots

Figure S12

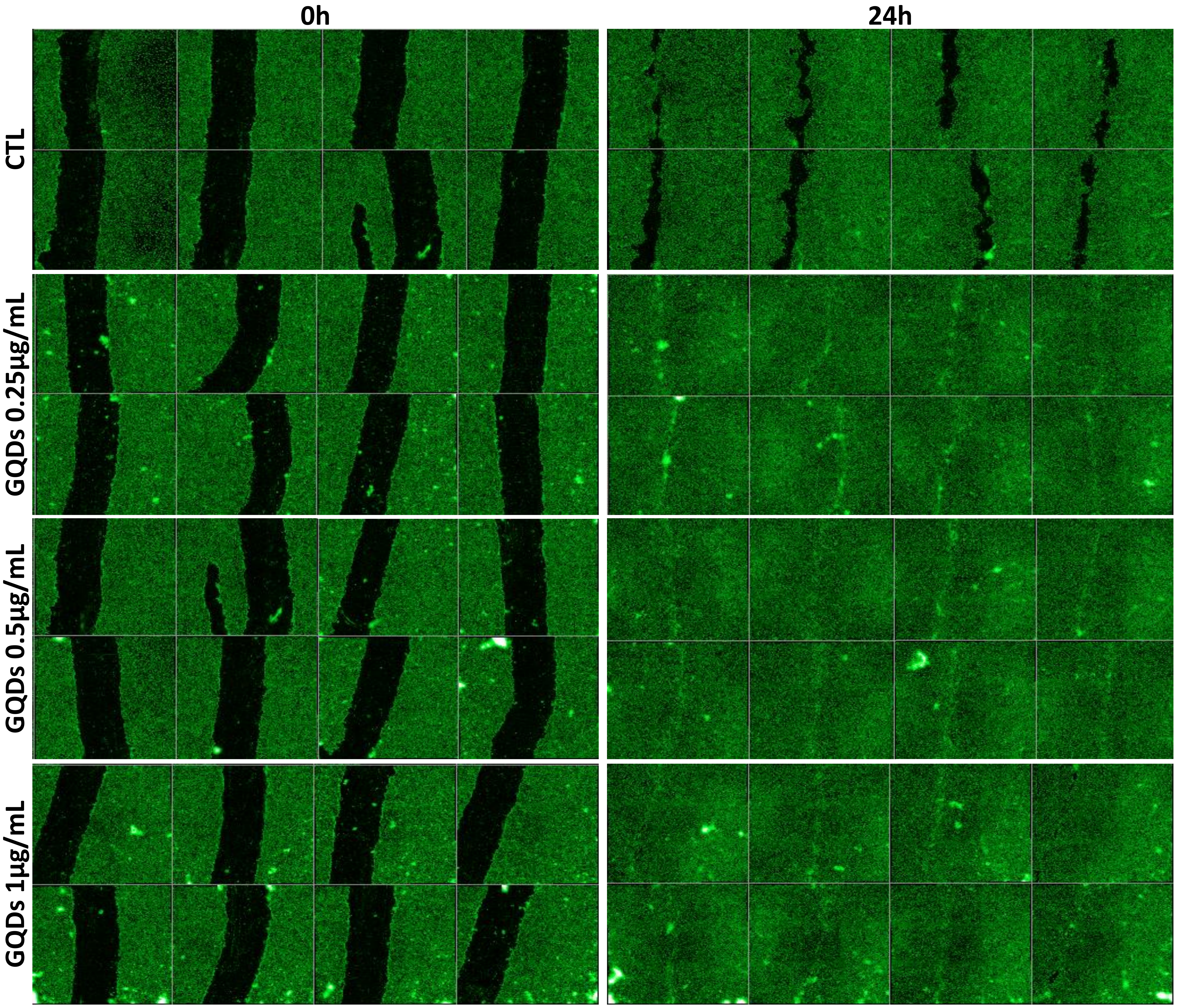

Figure S12. GQDs promote the wound-healing in podocytes. Representative images of podocyte wound healing at 0 to 24 h treated with 0.25, 0.5, and 1 μg mL^{-1}. GQDs, graphene quantum dots

Figure S13

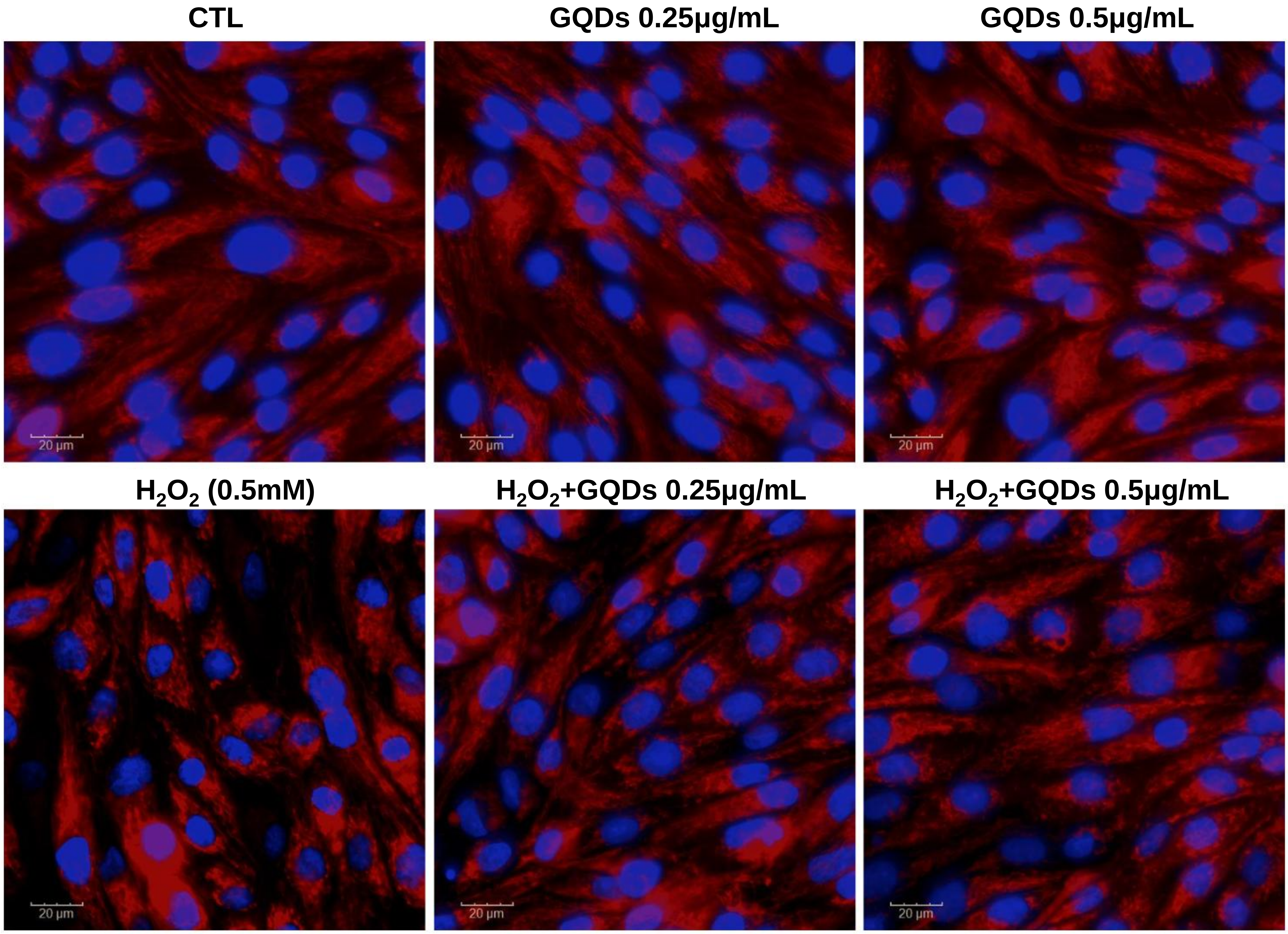

Figure S13. GQDs promote mitochondrial renewal in human podocytes. Representative fluorescence images show MitoTracker Red after incubation with H_2O_2 (1 mM) and GQDs (0.25 and 0.5 μg mL^{-1}). GQDs, graphene quantum dots

Figure S14

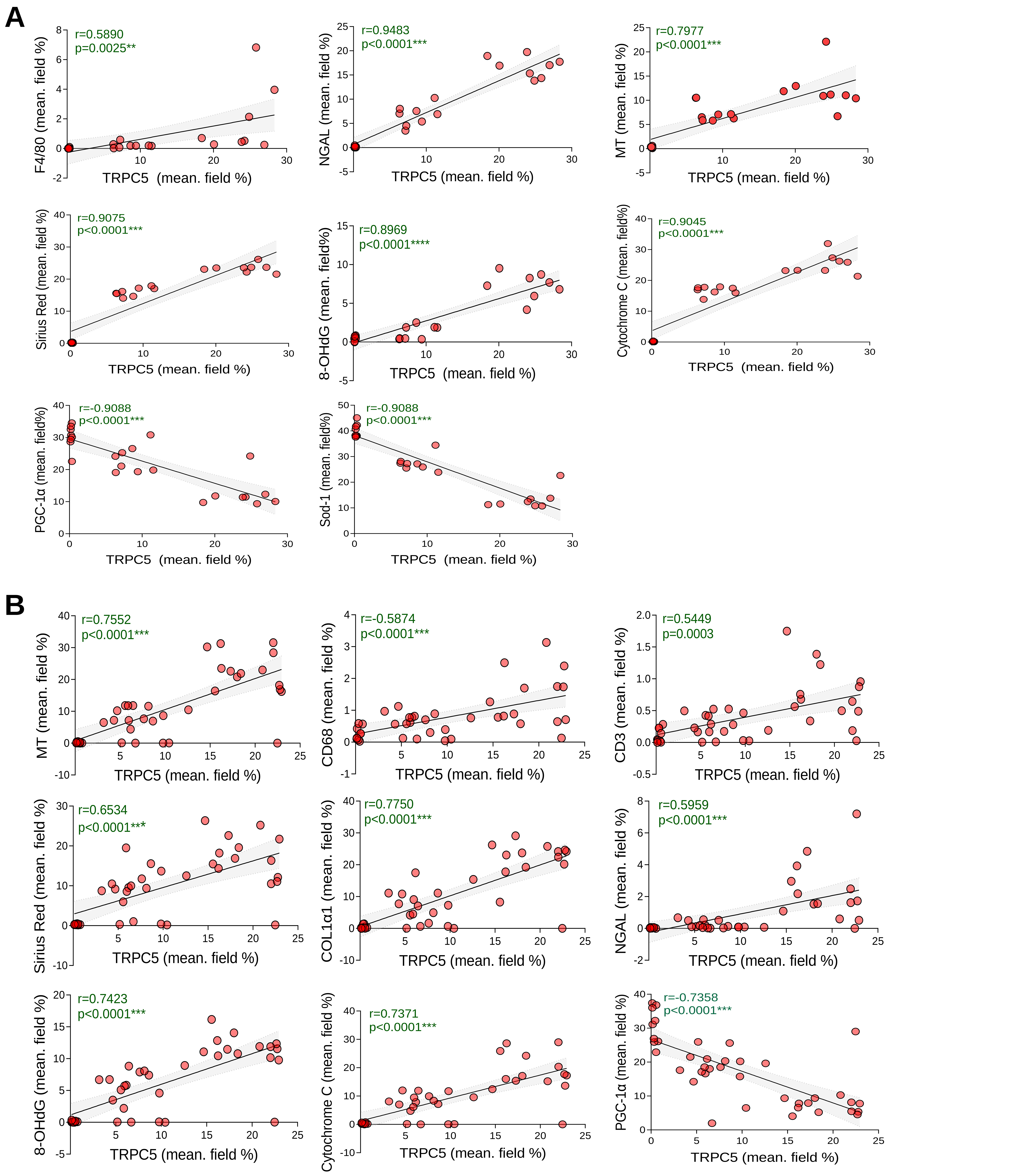

Figure S14. Relationship between the IHC results of certain targets in GQDs-treated ADN mice (A), and GQDs-treated 5/6Nx rat (B) with TRPC5. The regression lines are plotted from n = 24 (A) and n = 40 (B) per group.

Figure S15

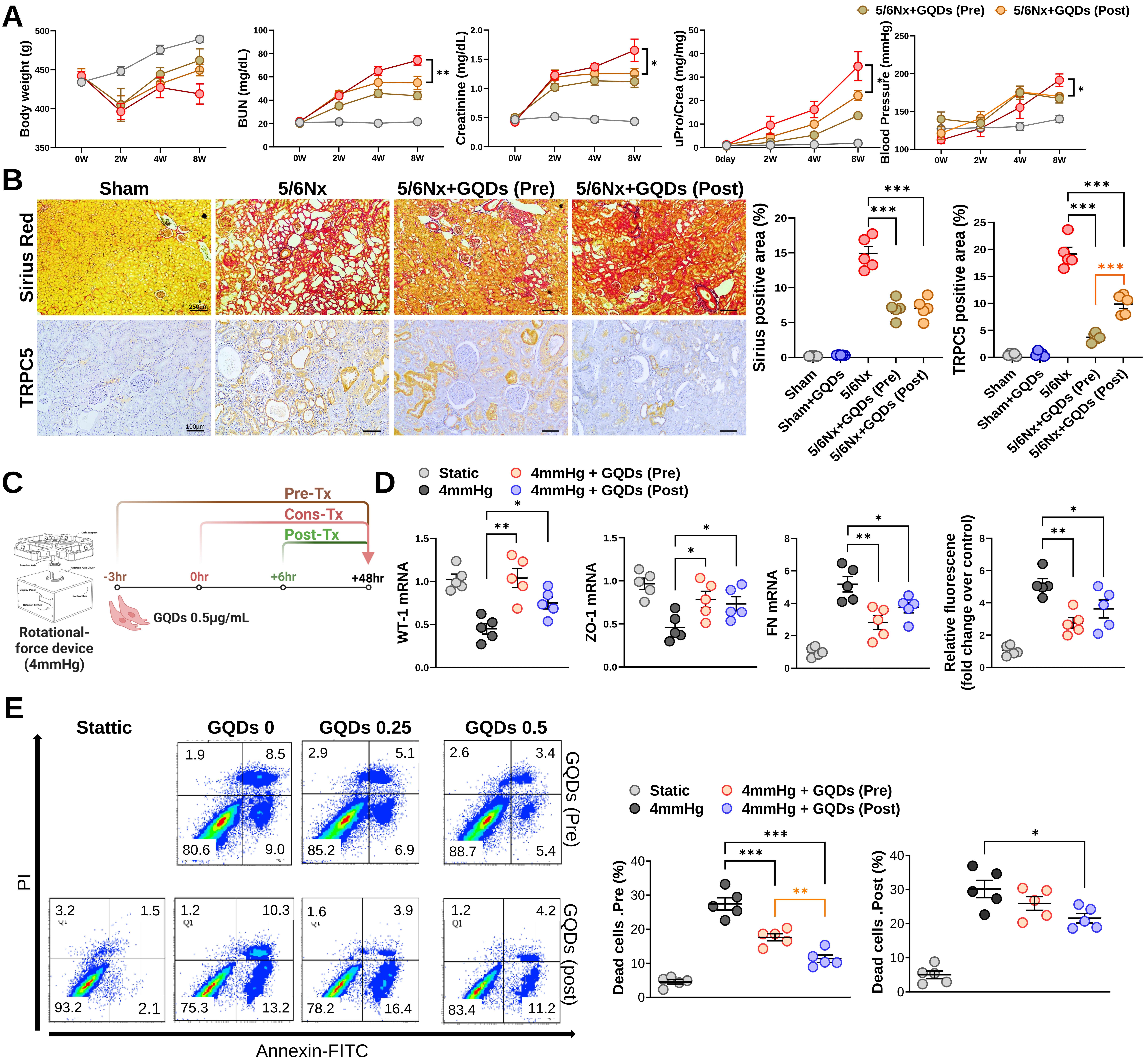

Figure S15. Pre-treatment with GQDs demonstrates greater therapeutic effectiveness in the 5/6Nx model, rescuing cell viability in a device-based hypertension condition. (A) Kidney function indicators. Sham, 5/6Nx, and 5/6Nx+GQDs (pre and post) (n = 5 per group). (B) Representative IHC images and quantitative data show the expression of Sirius Red and TRPC5 in the 5/6Nx+GQDs model (n = 5 per group). (C) A timeline of the device-based approach for hypertension experiments in human primary culture podocytes (Tx: Treatment & Cons: concomitant). GQDs (0.5 μg mL^{-1}) utilized in various time interval groups. (D) Fold changes in relative fluorescence or mRNA profiles associated with the podocyte's response to injury under rotational force stress (n = 5 per group). (E) Annexin V/propidium iodide staining shows podocyte apoptosis after 48 h of fibrosis induction via rotational force stress (4 mmHg) and treatment with GQDs (025 or 0.5 μg mL^{-1}), with quantitative data displayed over time and in a dose-dependent manner (n = 5 per group). Experiments were repeated at least three times, and the data are shown as the mean ± standard error of the mean. *$p < 0.05$, **$p < 0.01$, ***$p < 0.001$.

Figure S16

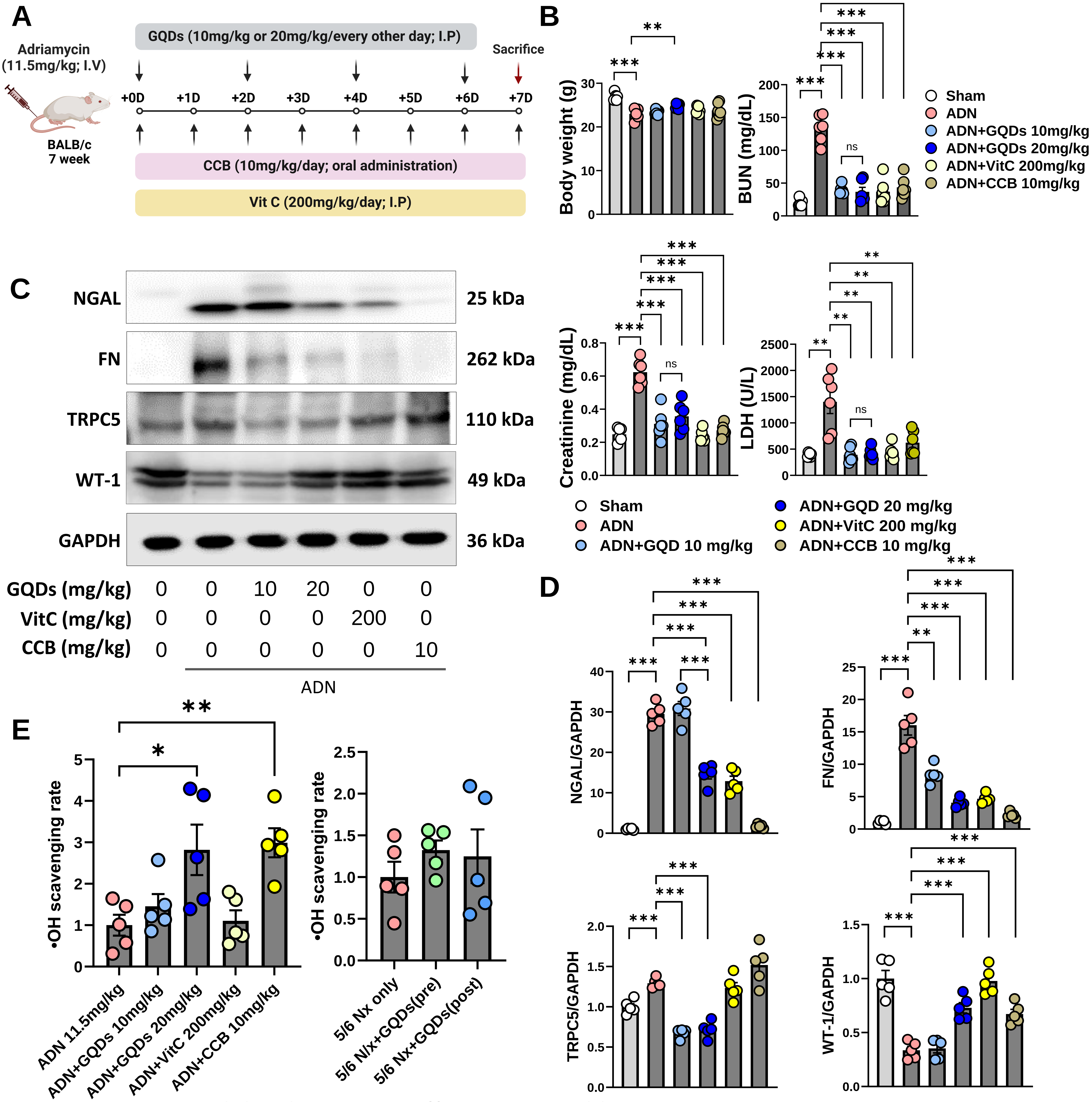

Figure S16. GQDs exhibit therapeutic effects comparable to standard agents, vitamin C and CCB. (A) Experimental schematic of BALB/c mice (7W, Male) treated with Adriamycin (11.5 mg kg^{-1}, I.V), GQDs (10 or 20 mg kg^{-1}, I.P), vitamin C (200 mg kg^{-1}, I.P), and CCB (10 mg kg^{-1}, P.O). (B) Body weight (g), BUN (mg/dL), creatinine (mg/dL), and LDH (U/L) levels across CTL and experimental mice groups (n = 6 per group). (C & D) Represent images of Western blot images (C) and their quantification (D), normalized to GAPDH levels (n = 5 per group). (E) Antioxidant capacity of GQDs in relation to vitamin C and CCB using the HORAC activity assay in plasma from ADN mice model and 5/6Nx rat model (n = 5 per group). The data are shown as the mean ± standard error of the mean. **p* < 0.05, ***p* < 0.01, ****p* < 0.001.

Figure S16 continued...

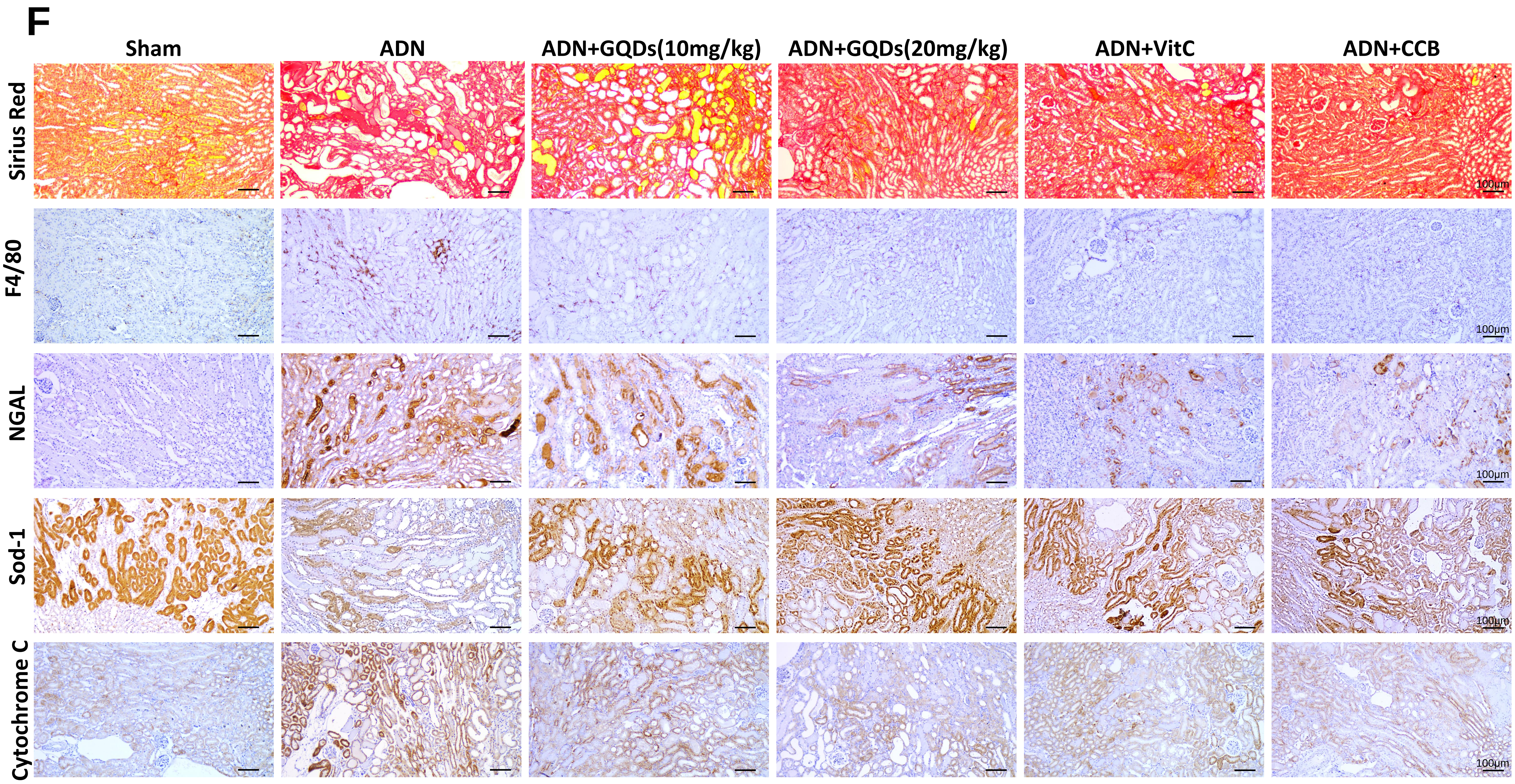

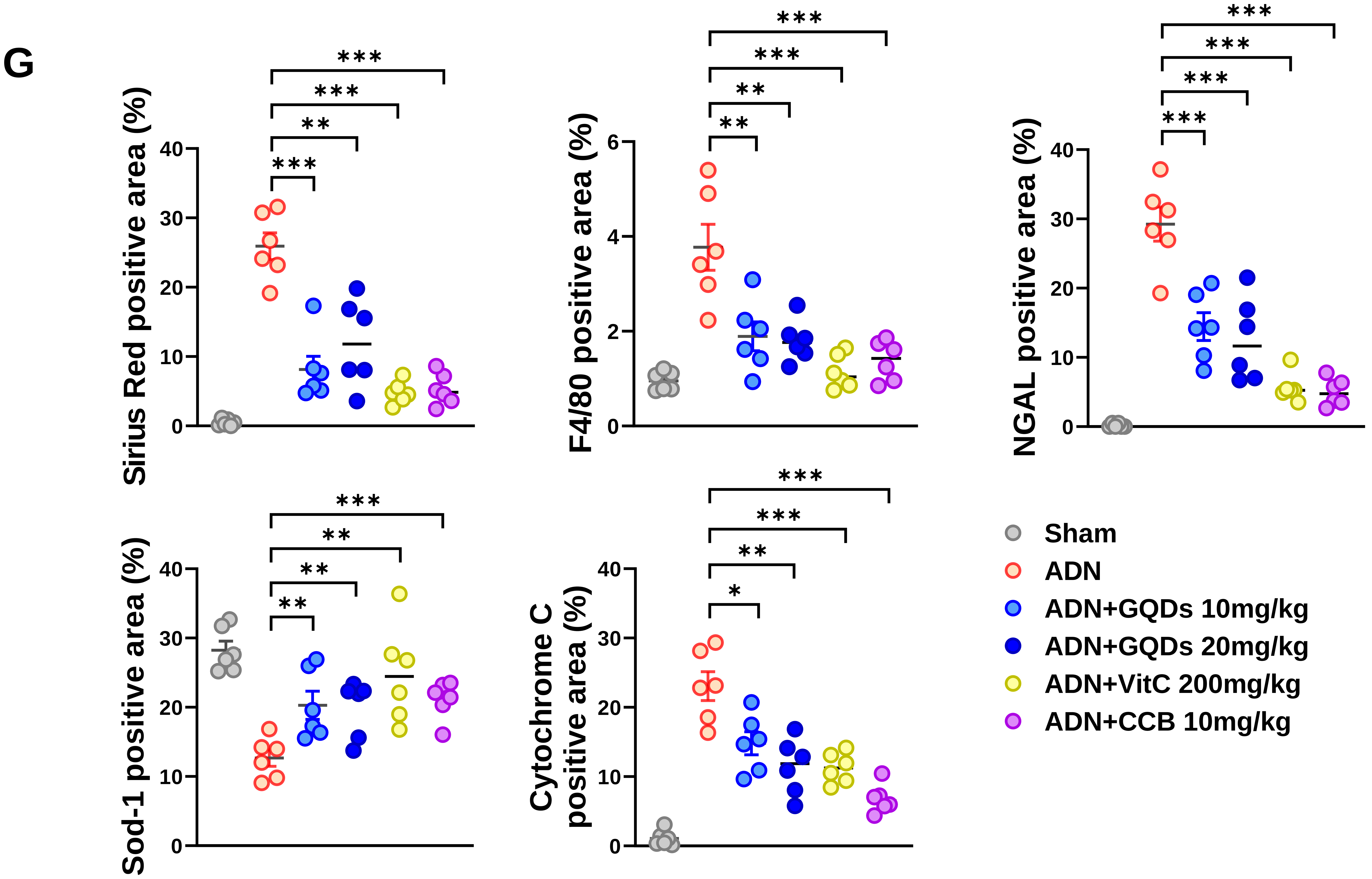

Figure S16. GQDs exhibit therapeutic effects comparable to standard agents, vitamin C and CCB. (F & G) Representative immunohistochemistry images and quantitative data of Sirius Red, F4/80, NGAL, Sod-1, and Cytochrome C (n = 6 per group). The data are shown as the mean ± standard error of the mean. **p < 0.05, **p < 0.01, ***p < 0.001*.

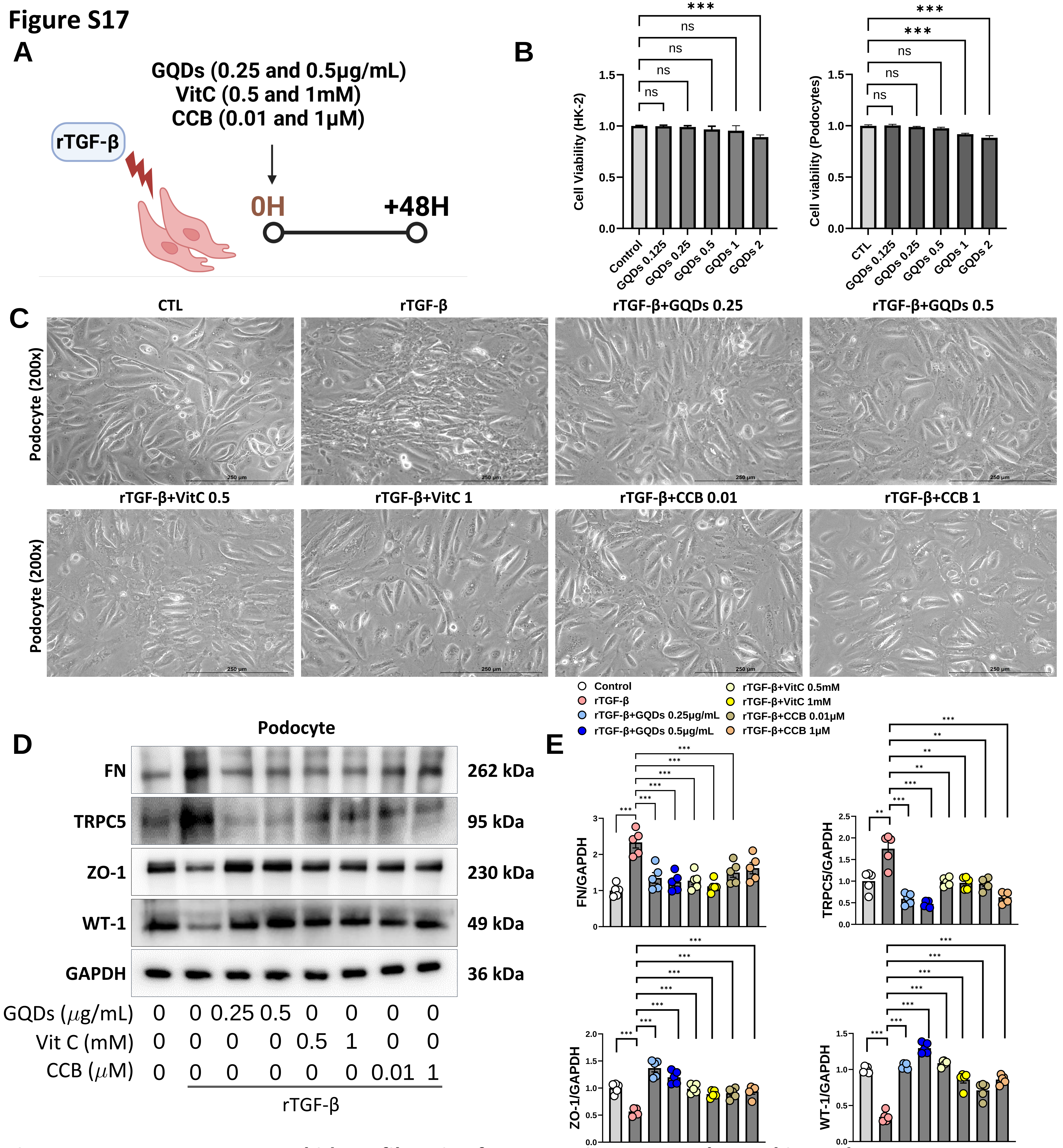

Figure S17. GQDs attenuate kidney fibrosis after targeting TRPC5 channel in podocytes. (A) Schematic representation of the of *in vitro* fibrosis model with treatments of GQDs (0.25 or 0.5 μg mL^{-1}), vitamin C (0.5 or 1 mM), and CCB (0.01 or 1 μM). (B) Cell viability assessment across different doses of GQDs (0.125, 0.25, 0.5, 1, and 2 μg mL^{-1}). (C) Representative cell images following exposure to rTGF-β and GQDs (0.25 or 0.5 μg mL^{-1}), vitamin C (0.5 or 1 mM), and CCB (0.01 or 1 μM). (D and E) Representative Western blot images (D) and corresponding quantifications (E), normalized to GAPDH (n = 5 per group). The data are shown as the mean ± standard error of the mean. *$*p < 0.05$, $**p < 0.01$, $***p < 0.001$*.

Figure S18

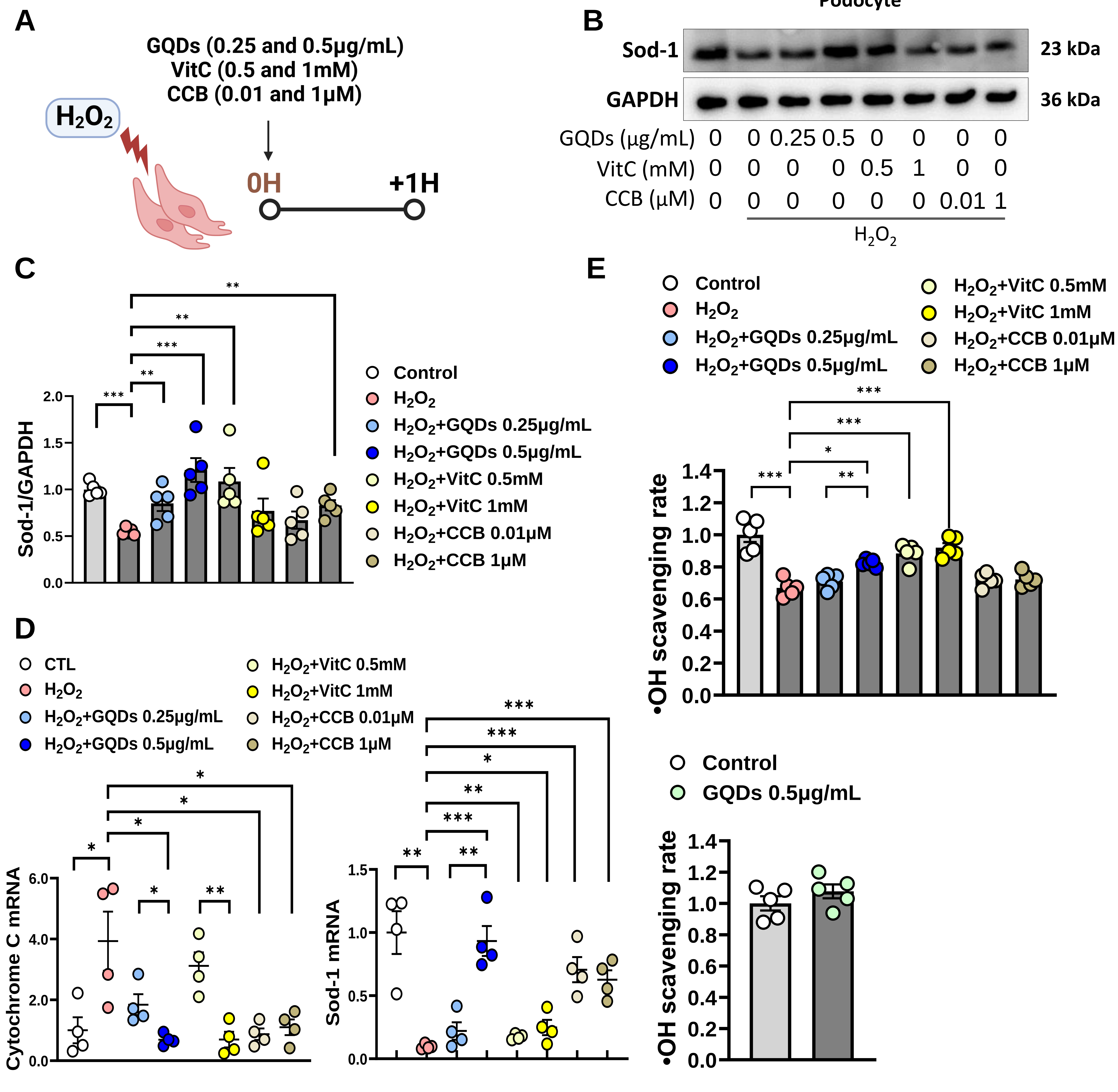

Figure S18. GQDs exhibit antioxidative effects in podocytes (A) Illustration of the *in vitro* oxidative stress model with treatments of GQDs (0.25 or 0.5 μg mL^{-1}), vitamin C (0.5 or 1 mM), and CCB (0.01 or 1 μM). H_2O_2 (1 mM) was applied for 1 h. (B and C) Representative Western blot images (B) and corresponding quantifications (C), normalized to GAPDH (n = 5 per group). (D) Fold changes in mRNA profiles associated with the podocyte's response to treatment of H_2O_2 (1 mM) with GQDs (0.25, 0.5 μg mL^{-1}), vitamin C (0.5, 1mM), or CCB (0.01, 1μM) (n = 4 per group). (E) Result of hydroxyl radical scavenging in podocytes cotreated with H_2O_2 (1 mM) and GQDs (0.25, 0.5 μg mL^{-1}), vitamin C (0.5, 1mM), and CCB (0.01, 1μM) (n = 5 per group). The data are shown as the mean ± standard error of the mean. *$*p < 0.05$, $**p < 0.01$, $***p < 0.001$*.

Table S1. Reagents for GQDs Quality Control Experiments

Product	Vendor	Cat #
PBS (Phosphate buffered saline, tablet)	Sigma-Aldrich	P4417
NaCl (Sodium Chloride, 99% (T), EP)	SAMCHUN chemcial	S0476
KCl (Potassium chloride, 99%)	SAMCHUN chemical	P0831
$MgCl_2$ (Magnesium chloride hexahydrate, 98%)	SAMCHUN chemical	M0038
$CaCl_2$ (Calcium chloride dihydrate powder, 71-77.5%, EP)	SAMCHUN chemical	C0090
$CaSO_4$ (Calcium sulfate dihydrate, ReagentPlus, >= 99%)	Sigma-Aldrich	C3771
EDTA (Ethylenediaminetetraacetic acid, disodium salt dihydrate. 99+%, ACS reagent)	ThermoFisher Scientific	40997

Table S2. Agents with Antifibrotic and Antioxidant Properties

Product	Vendor	Cat #
Amlodipine besylate	Supelco	PHR1185
Doxorubicin Hydrochloride (Adriamycin)	TCI	D4193
Vitamin C	Sigma-Aldrich	A5960

Table S3. Antibodies used for identifying human primary cells

Applications	Antibodies	Vendor	Cat.#	Fluorescence	Host	Reactivity
FACs	Podocalyxin	Fisher Scientific	50-112-26	PE	M	H
FACs	Aquaporin-1	Novus Biologicals	18012047	Alexa Fluor 488	R	H

R, Rat, M, Mouse, H, Human

Table S4. Media and Treatment Reagents for *in vitro* studies

Product	Vendor	Cat #.
DMEM/F12	Biowest	L0092
DMEM high glucose	Biowest	L0103
Hydrogen peroxide 30%	Supelco	K54376509
REGM	Lonza	CC3191
rhTGF-β1	R&D systems	240-B
rmTGF-β1	R&D systems	7666-MB

Table S5. Primary antibodies

Figure number	Applications	Antibodies	Vendor	Cat.#	Country/Cities	Host	Reactivity
1, 3, S10, S16, S17, S18	WB	GAPDH	Cell Signaling	2118S	USA, Danvers	Rabbit	H, M, R
1 , S3	WB, IF	KLF15	SantaCruz	Sc-271675	USA, Dallas	Mouse	H, M, R
1, S3, S9	WB	a-SMA	Abcam	Ab32575	UK, Cambridge	Rabbit	H, M, R
1, S3	WB	ICAM-1	Abcam	Ab171123	UK, Cambridge	Mouse	M, R
1, 3, 4, S3, S9, S16, S17	WB, IHC	TRPC5	Abcam	Ab240872	UK, Cambridge	Mouse	M, R
1, S3, S9	WB	Bax2	Cell Signaling	2772S	USA, Danvers	Rabbit	H, M, R
1, S3, S9	WB	BCL2	SantaCruz	Sc-23960	USA, Dallas	Mouse	H, M, R
S9	WB	P53	Cell Signaling	2524s	USA, Danvers	Mouse	H, M, R
S9	WB	P21	SantaCruz	Sc-6246	USA, Dallas	Mouse	H, M, R
1, 4, S3, S9	WB	COL1a1	SantaCruz	Sc-293182	USA, Dallas	Mouse	H, M, R
1, 9, S3, S16, S17	WB	FN	Abcam	Ab2413	UK, Cambridge	Rabbit	H, M
S10	WB	TRPC6	LS Bio	LS-C312900	USA, Washington	Rabbit	M, R
3, 4	IHC	8’-OHdG	Abcam	Ab48508	UK, Cambridge	Mouse	M
4	IHC	CD68	Abcam	Ab31630	UK, Cambridge	Mouse	H, M, R
6	IHC	Sod-1	Aviva system Biology	OABB00304	USA, San Diego	Rabbit	H, M, R
1, S3	WB	WT-1	Abcam	ab89901	UK, Cambridge	Rabbit	H, M
1, 3, 4, S9, S16	WB, IHC	NGAL	SantaCruz	sc515876	USA, Dallas	Mouse	M, R
1, S3	WB, IF	Zo-1	Abcam	Ab59720	UK, Cambridge	Rabbit	H, M
3, 9, S9, S11	WB	β-actin	Sigma	A1978	USA, St. Louis	Mouse	H, M, R
6	IHC	Cytochrome C	Sigma	MA5-20330	USA, St. Louis	Mouse	H, M
S9	WB	Nephrin	Invitrogen	PA5-20330	USA, Massachusetts	Rabbit	H, M, R
6	IHC	PGC-1	SantaCruz	Sc-518025	USA, Dallas	Mouse	H, M, R
4	IHC	CD3e	Abcam	Ab5690	UK, Cambridge	Rabbit	H, M, R
1, S3	WB	Kim-1 (TIMP1)	R&D systems	AF1817	USA, Minneapolis	Goat	M
3, S16, S17	WB, IF	WT-1	Abcam	Ab180840	UK, Cambridge	Rabbit	H, M
9, S17	WB	TRPC5	Alomone labs	ACC-020	Jerusalem, Israel	Rabbit	H, M, R
S11	WB	CCN1	Abcam	Ab24448	UK, Cambridge	Rabbit	H, M
S11	WB	B-galactosidase	Cell Signaling	27198	USA, Danvers	Rabbit	H, M, R
S17	WB	ZO-1	Proteintech	21773-1-AP	USA, San Diego	Rabbit	H, M, R
S18	WB	Sod-1	SantaCruz	Sc-101523	USA, Dallas	Mouse	H, M, R

H, Human; M, Mouse; R, Rat

Table S6. Staining kit and secondary antibodies for IHC experiments

Product	Vendor	Cat #.
Picro Sirius Red Stain Kit	Abcam	Ab150681
EnVision + System-HRP Labelled Polymer Anti-Rabbit	Agilent Dako	K4003
EnVision + System-HRP Labelled Polymer Anti-Mouse	Agilent Dako	K4001

Table S7. HRP-conjugated secondary antibodies for western blotting

Product	Vendor	Cat #.
Anti-mouse IgG, HRP-linked Antibody	Cell Signaling Technology	7076
Anti-rabbit IgG, HRP-linked Antibody	Cell Signaling Technology	7074

Table S8. RT-PCR kit

Product	Vendor	Cat #.
Reverse Transcription System	Promega	A3500

Table S9. Primer sequences

Genes	Species	Forward (5'-3')	Reverse (5'-3')
GAPDH	Human	TCGACAGTCAGCCGCATCT	CCGTTGACTCCGACCTTCA
FN	Human	CCACCCCCATAAGGCATAGG	GTAGGGGTCAAAGCACGAGTCATC
IL-17R	Human	AGACACTCCAGAACCAATTCC	TCTTAGAGTTGCTCTCCACCA
P21	Human	GAGGCCGGGATGAGTTGGGAGGAG	CAGCCGGCGTTTGGAGTGGTAGAA
IL-8	Human	ATGACTTCCAAGCTGGCCGTGGCT	TCTCAGCCCTCTTCAAAACTTCTC
TRPC5	Human	CCCTGAAGATTGTGGCCTATG	AACAGGGATATGAGACGCAAC
H2AX	Human	TCTGTTCTAGTGTTTGAGCCG	CACTGGGAACTGGAGGC
Zo-1	Human	GCAGCCACAACCAATTCATAG	GAAAGGTAAGGGACTGGAGATG
β-actin	Human	GGGTCAGAAGGATTCCTATGG	CTCCTTAATGTCACGCACGATTTC
WT-1	Human	GAAAATAGGGGATGGTCCAG	CAATGGATTTCCTCACCCAG
IL-6	Human	GGTACATCCTCGACGGCATCT	GTGCCTCTTTGCTGCTTTCAC
Cytochrome C	Human	AAGGGAGGCAAGCACAAGACTG	CTCCATCAGTGTATCCTCTCCC
SOD-1	Human	GTGTGGCCGATGTGTCTATT	GCGTTTCCTGTCTTTGTACTTTC
PGC-1α	Human	AGCTTTGGCTTTACGGAATACCA	CCACAGGATAAGTCACCGAGGA

Table S10. Chemicals for immunofluorescence staining

Product	Vendor	Cat #.
DAPI	Simga-Aldrich	D9542
Phalloidin Alexa Flour 488	Abcam	A12379

Table S11. Secondary antibodies for immunofluorescence staining

Figure number	Applications	Antibodies	Vendor	Cat.#	Country/Cities	Host	Reactivity
1	IF (WT-1)	Alexa Flour 488	Invitrogen	A11070	USA, Massachusetts	Goat	R
1	IF (KLF15)	Alexa Flour 555	Invitrogen	A1990314	USA, Massachusetts	Goat	M
1	IF (WT-1)	Alexa Flour 647	Invitrogen	A21242	USA, Massachusetts	Goat	M
1	IF (Zo-1)	Alexa Flour 555	Invitrogen	A21428	USA, Massachusetts	Goat	R

R, Rat, M, Mouse, H, Human

Table S12. Mitochondria tracers

Product	Vendor	Cat #.
DAPI	Invitrogen	R37605
MitoTracker Green/ Deep red	Invitrogen	M7514/ M22426
MitoSox	Invitrogen	M36008
TMRM	Invitrogen	I34361

Table S13. Antibodies for Flow Cytometry

Applications	Antibodies	Vendor	Cat.#	Flouresence	Host	Reactivity
FACs	CD3e	BD pharmingen	553067	Percp	HM	M
FACs	CD4	Invitrogen	11-0041-82	FITC	R	M
FACs	CD8e	BD pharmingen	552877	PE-Cy7	R	M
FACs	CD25	BD Horizon	562606	BV421	R	M
FACs	CD11b	Invitrogen	12-0112-83	PE	R	M
FACs	CD206	Invitrogen	17-2061-82	APC	R	M
FACs	CD44	eBioscience	12-0441-83	PE	R	H,M
FACs	GR-1	eBioscience	11-5931-82	FITC	R	M

R, Rat, M, Mouse, H, Human, HM, Hamster

Table S14. Annexin V/PI staining kit

Product	Vendor	Cat #.
FITC Annexin V Apoptosis Detection Kit	BD Biosciences	556547

Table S15. Intracellular calcium indicator kit

Product	Vendor	Cat #.
Fura 2AM	Abcam	Ab176766
Ionomycin	Sigma-Aldrich	10634

Table S16. Mitochondrial respiratory assay

Product	Vendor	Cat #.
JC-1 assay kit	Invitrogen	M34152
Seahorse XF ATP Real-Time rate assay	Agilent	103592

Table S17. ROS assay

Product	Vendor	Cat #.
Human Serum Albumin Protein (Rhodamine)	Abcam	Ab8031
MTS	DOGenBio	EZ-3000
ROS assay	Cell Biolabs	STA-342

Table S18. Assay for senescence

Product	Vendor	Cat #.
Live/Dead	Invitrogen	L3224
Senescence β-Galactosidase Staining Kit	Cell Signaling	#9860

Table S19. Assay for hydroxyl radical antioxidant capacity

Product	Vendor	Cat #.
OxiSelect^{TM} Hydroxyl Radical Antioxidant Capacity (HORAC) Activity Assay	CELL BIOLABS	STA-346

Table S20. Analysis for gene expression clustering and associated biological process in GQDs-treated ADN mice

GO terms	Target genes	Log2FC	p.Adjust
**Intrinsic apoptotic regulating pathway (GO:0097193)**	Ddx3x	-0.387	0.023
	Hnrnpk	-0.448	0.015
	Bid	-0.458	0.004
	Siah1a	-0.465	0.007
	Xpa	-0.523	0.005
	Aldh2	-0.638	0.006
	Ptpn1	-0.795	0.000
	Ddit4	-1.131	0.000
**Response to oxidative stress (GO:0006979)**	Prkcd	-0.387	0.023
	Hdac6	-0.448	0.015
	App	-0.458	0.004
	Ptk2b	-0.465	0.007
	Cd36	-0.524	0.005
	Gclc	-0.638	0.006
	Txn1	-0.795	0.000
	Prdx6	-1.131	0.000
**Calcium ion transport (GO:0006816)**	Slc30a1	0.330	0.032
	Bspry	-0.349	0.031
	Trpv5	-0.378	0.017
	Tspo	-0.384	0.015
	Coro1a	-0.401	0.026
	Tmem37	-0.438	0.012
	P2rx4	-0.480	0.003
	Fkbp1a	-0.494	0.003
	Tgfb1	-0.521	0.002
	Gnai2	-0.521	0.006
	Icam1	-0.530	0.006
	Ccl5	-0.532	0.036
	Pacsin3	-0.543	0.001
	G6pdx	-0.548	0.005
	Bak1	-0.617	0.000
	Creb3	-0.694	0.002
	Lgals3	-0.836	0.000
	F2r	-0.856	0.000
	Agt	-0.910	0.000
	Pdgfb	-1.225	0.000
	Cxcl10	-1.484	0.000
	Ccl2	-1.854	0.000
	Serpine1	-2.065	0.000
**Mitochondrial translation (GO:0032543)**	Mrpl12	0.589	0.000
	Rmnd1	0.587	0.001
	Ptcd3	0.515	0.001
	Mrpl18	0.489	0.002
	Mrpl50	0.358	0.021
	Mtg2	0.415	0.021
	Mrps2	0.223	0.037
	Shmt2	0.323	0.049
	Tufm	0.319	0.044
	Lrpprc	0.315	0.039

Table S21. Analysis for gene expression clustering and associated biological process in GQDs-treated ADN mice

GO terms	Target genes	Log2FC	p.Adjust
**ATP metabolic process (GO:0046034)**	Ak4	2.226	0.000
	Khk	0.935	0.000
	Gpd1	0.866	0.000
	Fam20c	0.695	0.000
	Enpp3	0.694	0.000
	Prkaa2	0.633	0.000
	Pfkm	0.621	0.002
	Ak3	0.561	0.007
	Pgam2	0.458	0.008
	Tfkc	0.455	0.008
	Atp6v1a	0.395	0.032
	Ncor1	0.374	0.042
	Ndufb10	0.337	0.036
**Regulation of the apoptotic signaling pathway (GO:2001233)**	Mapk8ip1	0.354	0.020
	Aatf	-0.322	0.050
	Ctsc	-0.332	0.038
	Ybx3	-0.332	0.037
	Mnt	-0.357	0.017
	Yap1	-0.379	0.014
	Ppp2r1a	-0.400	0.016
	Psmd10	-0.415	0.013
	Pea15a	-0.427	0.027
	Daxx	-0.437	0.005
	Cttn	-0.452	0.016
	Ppp2r1b	-0.460	0.014
	Sh3glb1	-0.480	0.006
	Fgfr1	-0.491	0.001
	Hspb1	-0.520	0.010
	Tmbim1	-0.521	0.049
	Gnai2	-0.530	0.006
	Thbs1	-0.533	0.001
	Kdm1a	-0.543	0.001
	Nupr1	-0.595	0.002
	Ltbr	-0.6064	0.000
	Lmna	-0.631	0.000
	Dab2	-0.646	0.003
	Mdm2	-0.665	0.000
	Mif	-0.665	0..000
	Creb3	-0.694	0.002
	Bcl10	-0.715	0.000
	Plscr1	-0.724	0.000
	Tgfbr1	-0.784	0.000
	Src	-0.785	0.000
	Itga6	-0.822	0.000
	Lgals3	-0.836	0.000
	Mcl1	-0.891	0.000
	Agt	-0.906	0.00
	Bcl2l1	-0.942	0.000
	Ptpn2	-0.961	0.000
	Pamip1	-0.967	0.000
	Fas	-0.973	0.000
	Xbp1	-1.152	0.000
	Clu	-1.202	0.000
	Myc	-1.280	0.000
	Rela	-1.341	0.000
	Cd74	-1.492	0.000

Table S22. Analysis for gene expression clustering and associated biological process in GQDs-treated ADN mice

GO terms	Target genes	Log2FC	p.Adjust
**Regulation of the apoptotic signaling pathway (GO:2001233)**	Tnfrsf12a	-1.539	0.000
	Plaur	-1.743	0.000
	Ler3	-1.780	0.000
	Atf3	-1.831	0.000
	Tnfainp3	-2.051	0.0000
	Icam1	-2.061	0.000
	Serpine1	-2.065	0.000
	Inhbb	-2.082	0.000
	Hmox1	-2.515	0.000
	Fgg	-2.599	0.000
	S100a9	-2.750	0.000
	Fga	-3.030	0.000
	S100a8	-3.151	0.000
	Fgb	-3.646	0.000
**Monoatomic ion channel activity (GO:0005216)**	Clcn3	0.395	0.011
	Clcnka	1.005	0.000
	Kcnk5	-0.718	0.000
	P2rx4	-0.479	0.003
	Itpr1	0.332	0.044
**Podocyte differentiation (GO:0072112)**	Ext1	-0.389	0.013
	Klf15	0.581	0.001
	Podxl	0.685	0.000

Table S23. Real-time measurements of OCR in podocytes

	CTL		H_2O_2		H_2O_2+GQDs 0.25		H_2O_2+GQDs 0.5	
**Parameter**	**Mean**	**SEM**	**Mean**	**SEM**	**Mean**	**SEM**	**Mean**	**SEM**
**Basal**	25.07	1.02	9.84	0.79	24.24	1.99	26.15	1.09
**Proton leak**	5.44	1.02	3.76	0.41	6.66	0.71	7.37	0.59
**Maximal respiration**	100.64	2.24	19.82	4.96	70.33	6.90	83.53	4.38
**Spare respiratory capacity**	75.56	2.14	9.98	4.51	46.09	5.17	57.38	3.67
**Non-mitochondrial oxygen consumption**	24.18	0.74	18.63	0.66	20.41	1.37	21.15	0.45
**ATP production**	19.63	0.35	6.08	0.61	17.58	1.73	18.79	0.90
**Coupling efficiency**	0.79	0.03	0.62	0.03	0.72	0.02	0.72	0.02
**Spare respiratory capacity**	4.05	0.15	1.94	0.47	2.88	0.13	3.20	0.11
